# Crystal-facet-directed all-vacuum-deposited perovskite solar cells

**DOI:** 10.1038/s41563-026-02494-w

**Published:** 2026-02-23

**Authors:** Xinyi Shen, Wing Tung Hui, Shuaifeng Hu, Fengning Yang, Junke Wang, Jin Yao, Atse Louwen, Bryan Siu Ting Tam, Lirong Rong, David P. McMeekin, Kilian Lohmann, Qimu Yuan, Matthew C. Naylor, Manuel Kober-Czerny, Seongrok Seo, Philippe Holzhey, Karl-Augustin Zaininger, M. Greyson Christoforo, Perrine Carroy, Vincent Barth, Fion Sze Yan Yeung, Nakita K. Noel, Michael Johnston, Yen-Hung Lin, Henry J. Snaith

**Affiliations:** 1 Clarendon Laboratory, Department of Physics, University of Oxford, Oxford, United Kingdom; 2 Department of Electronic and Computer Engineering, The Hong Kong University of Science and Technology, Hong Kong SAR, China; 3 State Key Laboratory of Displays and Opto-Electronics, The Hong Kong University of Science and Technology, Hong Kong SAR, China; 4 National Thin-Film Facility for Advanced Functional Materials, Department of Physics, University of Oxford, Oxford, United Kingdom; 5 Eurac Research, Bolzano, Italy; 6 Université Grenoble Alpes, CEA, Liten, Campus Ines, Le Bourget du Lac, France; 7 Present Address: RISE Research Institutes of Sweden, Umeå, Sweden; 8 Present Address: Department of Perovskite Tandem Solar Cells, Helmholtz-Zentrum Berlin für Materialien und Energie GmbH, Berlin, Germany

**Keywords:** Solar cells, Condensed-matter physics

## Abstract

Vacuum-based deposition is a scalable, solvent-free industrial method ideal for uniform coatings on complex substrates. However, all-vacuum-deposited perovskite solar cells fabricated by thermal evaporation trail solution-processed counterparts in efficiency and stability due to film quality challenges, necessitating advancement and improved understanding. Here, we report a co-evaporation route for 1.67-eV wide-bandgap perovskites by introducing a PbCl_2_ co-source to optimize film quality. We promote perovskite formation with pronounced (100) ‘face-up’ orientation and deliver a certified all-vacuum-deposited solar cell with 18.35% efficiency (19.3% in the laboratory) for 0.25-cm^2^ devices (18.5% for 1-cm^2^ cells). These cells retain 80% of peak efficiency after 1,080 h under the ISOS-L-2 protocol. Leveraging operando hyperspectral imaging, we provide spatiotemporal spectral insight into halide segregation and trap-mediated recombination, correlating microscopic luminescence features with macroscopic device performance while distinguishing radiative from non-ideal recombination channels. We further demonstrate 27.2%-efficient 1-cm^2^ evaporated perovskite-on-silicon tandem cells and outdoor stability of all-vacuum-deposited tandems in Italy, retaining ~80% initial performance after eight months.

## Abstract (web summary)

The vacuum process is scalable and solvent free, yet all-vacuum-deposited perovskite solar cells still trail solution-processed counterparts. Facet-directed co-evaporation yields (100)-oriented mixed-halide wide-bandgap films for efficient, stable single-junction cells and perovskite–silicon tandem cells.

## Main

Hybrid metal halide perovskites have redefined the limits of low-temperature-processed photovoltaics (PVs), achieving certified power conversion efficiencies of over 27% in single-junction cells and close to 35% in perovskite-on-silicon tandem cells^1,2^. Their low-temperature processability^3^ and bandgap (*E*_g_) tunability^4^ position them as ideal candidates for next-generation applications, from lightweight flexible modules^5,6^ to ultrahigh-efficiency multijunction architectures^7–10^. However, the dominant solution-based fabrication methods, which are responsible for most record-breaking devices, face scalability and manufacturing challenges, including the use of (toxic) solvents^11^, batch-to-batch variability^12^, spatial inhomogeneity^13^ and incompatibility with highly textured substrates—for instance, industry-standard pyramidal silicon^14^.

Vacuum thermal evaporation (VTE), a solvent-free, industry-proven deposition technique widely used in organic light-emitting diode (OLED) displays^15^, functional glass coatings^16^ and inorganic thin-film PVs^17^, offers a compelling pathway to overcome these limitations. Its inherent ability to conformally coat textured substrates makes it particularly suitable for perovskite-on-silicon tandems^18^. Although single-component layers are no doubt simpler, high-volume manufacturing exists for co-evaporation of multiple components—for instance, with copper–indium–gallium–selenide PVs^19^ and doped organic semiconductors in OLEDs^20^. Unlike the solution-processed counterparts that are typically formed from a mixture of the required precursors^21–23^, the VTE co-deposition of perovskite layers heavily relies on the fine control of each evaporation source^24^. Two hurdles that pose a critical challenge to forming homogeneous perovskite layers via VTE are the deposition of low-sublimation-temperature materials, such as methylammonium or formamidinium iodide (MAI or FAI)^25^, and the control of stoichiometry for target bandgaps^26^. Therefore, translating this potential into high-performance evaporated ‘baseline’ perovskite solar cells (PSCs) will be crucial for establishing industrially compatible VTE manufacturing paradigms and requires tremendous efforts on crystallization control^27^, halide segregation mitigation^28^ and grain orientation engineering to render high operational stability for perovskite absorbers^10,21^.

Here we demonstrate a multisource co-evaporation strategy that decouples stoichiometric precision from crystallization kinetics, enabling phase-stable 1.67-eV wide-bandgap (WBG) perovskites with dominant (100) ‘face-up’ orientation. By independently tuning six thermal sources, we achieve a maximum power point tracking (MPPT) efficiency (*η*_MPPT_) of 19.3% (18.35% certified, 0.25 cm^2^) and 18.5% (1 cm^2^) for ‘all-vacuum-deposited’ WBG cells. These cells retain 100% peak efficiency after 20,000 h of dark storage in nitrogen. Under the stringent ISOS-L-2 stressing protocols (ISOS, International Summit on Organic Photovoltaic Stability; full-spectrum simulated sunlight, open circuit (OC)) at 75 ± 5 °C and 65 ± 5 °C, our devices maintain 80% of peak *η*_MPPT_ after 1,080 h and 1,250 h, respectively, rivalling state-of-the-art solution-processed counterparts^29–34^. Operando hyperspectral imaging reveals suppressed compositional heterogeneity and non-radiative losses, directly linking facet-engineered microstructure to operational resilience. Critically, we integrate these advances into perovskite-on-silicon tandems that achieve substantial efficiency (27.2%, 1 cm^2^ with a solution-processed self-assembled monolayer (SAM) hole transport layer (HTL), and 24.3% with an all-vacuum-deposited device stack) and field-lifetime stability for the all-vacuum-deposited tandem, retaining ~80% of initial performance after eight months of continuous operation in Italy.

## Crystal-facet-directed co-evaporated WBG perovskites

**Fig. 1:**
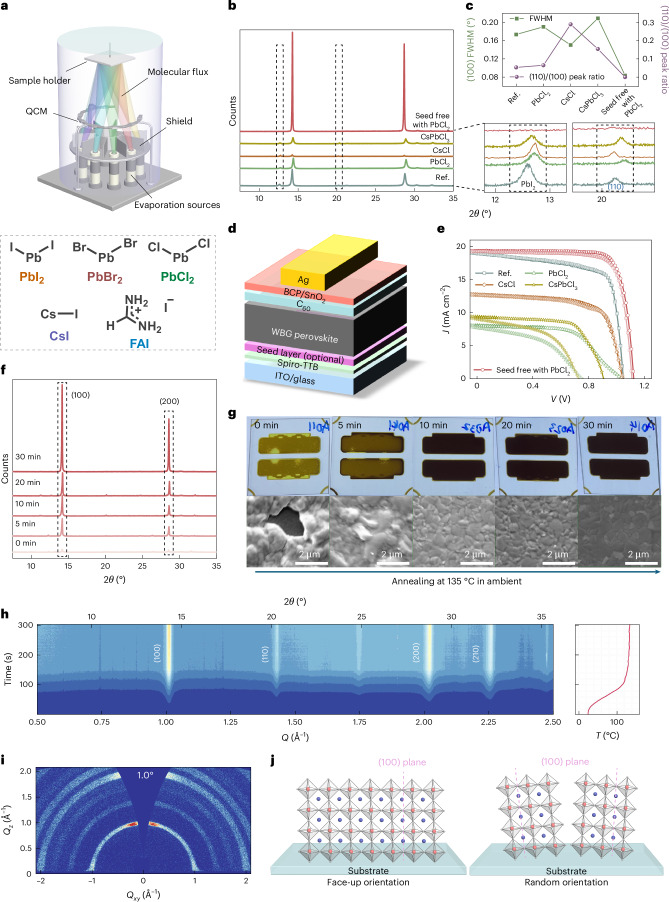
Crystal-facet-directed co-evaporated WBG perovskites. **a**, Schematic of a co-evaporation system with multiple VTE sources available for separate precursors. QCM, quartz-crystal microbalance. **b**, XRD patterns of 500-nm co-evaporated perovskite films of Cs_0.17_FA_0.83_Pb(I_0.80_Br_0.20_)_3_ (denoted as ‘Ref.’), with 10-nm seed layers of CsCl, PbCl_2_ and CsPbCl_3_ separately, to compare with a seed-free co-evaporated perovskite of Cs_0.17_FA_0.83_Pb(I_0.75_Br_0.20_Cl_0.05_)_3_ (denoted as ‘Seed free with PbCl_2_’). Certain diffraction angles are enlarged to show the PbI_2_ and (110) phases. **c**, FWHM and (110)/(100) peak ratios of the corresponding 500-nm co-evaporated perovskite films shown in **b**. **d**, Schematic of device architecture for all-vacuum-deposited WBG PSCs. The absorber layers are the corresponding 500-nm co-evaporated perovskites shown in **b**. The seed layers are only involved in the device stack if specified. Spiro-TTB, 2,2′,7,7′-tetra(*N*,*N*-di-*p*-tolyl)amino-9,9-spirobifluorene. **e** *J–V* characteristics of all-vacuum-deposited WBG PSCs using the device architecture shown in **d**. The absorber layers are the corresponding 500-nm co-evaporated perovskite films shown in **b**. **f**, XRD patterns of 500-nm seed-free, co-evaporated Cs_0.17_FA_0.83_Pb(I_0.75_Br_0.20_Cl_0.05_)_3_ perovskite films that were annealed at 135 °C in air (40–50% RH) for 0–30 min. **g**, Photos and top-view SEM images of the corresponding films shown in **f**, taken immediately after XRD measurements (~1.5 h of exposure to ambient conditions). **h**, GIWAXS pattern for the in situ annealing of an as-deposited co-evaporated FA_0.83_Cs_0.17_Pb(I_0.75_Br_0.20_Cl_0.05_)_3_ perovskite material at 135 °C in air (40–50% RH in the laboratory), which resembles the device fabrication conditions. **i**, Ex situ 2D GIWAXS intensity mapping from an incident angle of 1.0° for a post-annealed (for 30 min) co-evaporated Cs_0.17_FA_0.83_Pb(I_0.75_Br_0.20_Cl_0.05_)_3_ perovskite film of 500 nm. **j**, A schematic illustration of the face-up and random orientations of (100) crystal facets with respect to the ITO substrate.

Compared with dual-source-deposited MAPbI_3_ (*E*_g_ = 1.55 eV; ref. ^35^) or FAPbI_3_ (*E*_g_ = 1.48 eV; ref. ^36^), there are only a handful of reports on WBG perovskites fabricated via VTE^37–41^. One effective approach is to employ ‘seed’ layers to guide the crystal growth and improve perovskite film quality during VTE^37,42^. We screened seed-layer-assisted^37^ and seed-free co-evaporation routes^43^ to form ~1.67-eV perovskites suitable for perovskite-on-silicon tandems (Fig. 1a); the highest phase purity and performance were obtained for seed-free co-evaporation with a small PbCl_2_ co-source (5 mol%) (Supplementary Note 1 and Supplementary Figs. 1 and 2). We conduct X-ray diffraction (XRD) measurements on these evaporated perovskite films (Fig. 1b), in which we observe a preferential orientation along the (100) planes (2*θ* = 14.2–14.4°). Upon introducing Cl^−^ from either seed layers or a co-evaporated source, we detect a subtle shift of the (100) peak to higher reflection angles, implying Cl incorporation^44^. Furthermore, we identify PbI_2_ phase, which is photo-unstable and detrimental to device stability^45,46^, in the reference perovskite (that is, seed free with no PbCl_2_) and all the perovskites deposited on seed layers, but not in the PbCl_2_-added, seed-free co-evaporated perovskites (Fig. 1b). Moreover, the latter shows highly oriented, uniaxial domains along the (100) planes with negligible (110) peak (Fig. 1c). The full-width at half-maximum (FWHM) of the (100) peak is narrowed twofold compared with the films prepared using other protocols. To link the crystallographic properties and device performance, we fabricate all-vacuum-deposited PSCs with the architecture shown in Fig. 1d. While other devices suffer from substantial hysteresis and s-kinked behaviour, our PbCl_2_-added, seed-free co-evaporated cells deliver hysteresis-free current density*–*voltage (*J–V*) curves with the highest efficiency (Fig. 1e). Importantly, our seed-free co-evaporation enables straightforward ‘in situ’ bandgap tuning (~1.65–1.72 eV) without seed-layer-induced stoichiometric perturbations (Supplementary Fig. 3 and Supplementary Note 2)^37^.

We selected our ‘seed-layer-free’ process for further optimization and investigation. Hereafter, unless we specify otherwise, the targeted perovskite composition is FA_0.83_Cs_0.17_Pb(I_0.75_Br_0.20_Cl_0.05_)_3_. Taking advantage of humid air^44,47,48^, we adopt an ambient annealing procedure (135 °C in air, 40–50% relative humidity (RH)) and examine different post-annealing durations (0–30 min) for the perovskite films. The pre-annealed perovskite presents a (100)-dominant orientation (Fig. 1f), with no change in the orientation preference upon thermal treatment, as indicated by the relative intensities of (100) and (200) peaks, while the counts gradually increase with annealing, suggesting increased overall crystallinity. Compared with the unannealed case, 30-min annealing results in the (100) intensity increasing ~30-fold and the FWHM reducing by a factor of ~2, giving an overall integrated peak area increase of ~12.5-fold. For annealing times shorter than 10 min, we observe pinhole formation and decolouration of the perovskite films following ~1.5 h of ambient exposure, indicative of material degradation (Fig. 1g and Supplementary Fig. 4). The films with elongated annealing (that is, 20 and 30 min) show no decolouration.

To track crystallographic evolution during annealing, we perform in situ grazing-incidence wide-angle X-ray scattering (GIWAXS) measurements on as-deposited co-evaporated perovskites during slow heating to 135 °C in air (40–50% RH) (Fig. 1h). As the temperature *T* approaches 100 °C, scattering halos emerge, and upon reaching 135 °C the (100) peak intensity increases approximately fourfold. The dominant scattering in the *Q*_*z*_ direction (*Q*_*xy*_ = 0) is consistent with the final perovskites adopting a ‘face-up’ orientation, where the (100) planes lie along the out-of-plane direction, whose intensity is ~3.6 times that of the in-plane direction (Fig. 1i,j and Supplementary Figs. 5c and 6). These results confirm that extended annealing above the crystallization temperature is essential for obtaining crystallographically desirable, ambient-stable perovskite films^49^.

Relative to solution-processed analogues, our co-evaporated films show higher crystallinity and larger, flatter grains in scanning electron microscopy (SEM) images, spanning the entire perovskite film thickness (Supplementary Figs. 5–8 and Supplementary Note 3). Thus, our modified co-evaporation recipe, which differs from convention only in composition and trace PbCl_2_ addition, successfully overcomes the historic challenge that co-evaporated perovskites exhibit tiny, misorientated polycrystalline grains^39^.

## Ageing of perovskite films

**Fig. 2:**
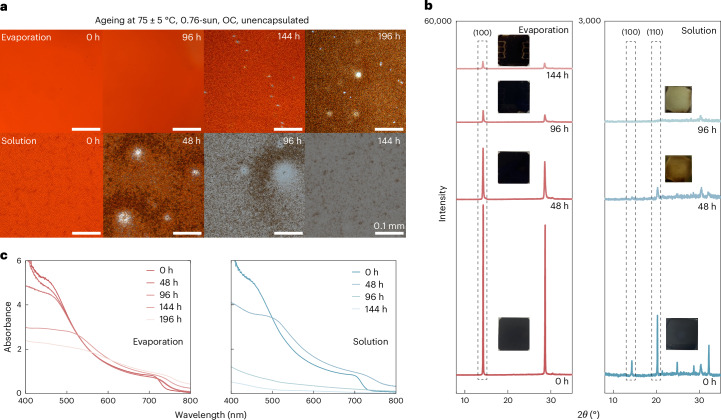
Perovskite film ageing characterization. **a**, Optical microscopic images of unencapsulated evaporated and solution-processed perovskite films using an absorber composition of FA_0.83_Cs_0.17_Pb(I_0.75_Br_0.20_Cl_0.05_)_3_, aged under the ISOS-L-2 protocol (full-spectrum simulated sunlight, 75 ± 5 °C, ambient air (50–60% RH in the laboratory)). A thin layer of poly(methyl methacrylate) was deposited on the films to protect them from dust and scratches. Photographs shown here were taken with an optical microscope with a ×20 magnification rate. Scale bar: 0.1 mm. **b**,**c**, Evolution of the XRD patterns (**b**) and the absorbance spectra (**c**) of the corresponding perovskite films shown in **a**. Inset: photographs of the perovskite films taken during ageing. The XRD patterns (**b**) in the logarithmic scale are shown in Supplementary Fig. 9 to reveal any impurity phases formed during ageing.

Given their improved crystallographic and morphological properties^21^, we investigate the stability of our evaporated perovskite films and devices. We age ‘bare’ perovskite films coated on indium tin oxide (ITO) substrates, with a thin poly(methyl methacrylate) coverage as rudimentary encapsulation, under the ISOS-L-2 procedure (75 ± 5 °C, 0.76-sun, in ambient (50–60% RH in the laboratory))^50^. Optical microscopy (Fig. 2a) reveals intense ‘surface wrinkling’ in fresh solution-processed films, a feature linked to local halide heterogeneity that accelerates light-induced degradation^51^. Such morphology is absent from evaporated films. Upon ageing, solution-processed films degrade rapidly, forming pinholes within 48 h and discolouring by 96 h, while evaporated films show no visible degradation until 196 h. XRD (Fig. 2b and Supplementary Fig. 9) confirms faster degradation of the cubic perovskite phase in solution-processed films. A PbI_2_ peak appears immediately in solution-processed films but only after 144 h for evaporated films. Conversely, aged evaporated perovskites exhibit minor additional peaks (2*θ* = 14.8° and 29.6°), probably corresponding to Br-rich perovskite phases from light- and heat-induced halide segregation^52^, which are absent from solution-processed perovskites, suggesting distinctive material degradation pathways. UV–Vis (Fig. 2c) shows a rapid absorbance drop at the band edge in solution-processed films within 48–96 h, signalling near-total loss of the photoactive phase, contrasted by a slow decline in evaporated films. These results collectively demonstrate the superior stability of evaporated perovskites, which probably stems from eliminating the use of highly coordinating solvents^53,54^ and improving the facet orientation and crystallinity (Supplementary Note 4 and Supplementary Figs. 10 and 11)^55,56^.

## Performance and operational stability of co-evaporated PSCs

Before we move on to assess the long-term stability in complete devices, we fabricate all-vacuum-deposited cells (Fig. 1d) and assess their performance. We also fabricate solution-processed PSCs of the same targeted composition on a [4-(3,6-dimethyl-9H-carbazol-9-yl)butyl]phosphonic acid (Me-4PACz) HTL with the same stacks, to compare the device performance and stability (see Supplementary Fig. 12 for the reason for the choice of HTL).

**Fig. 3:**
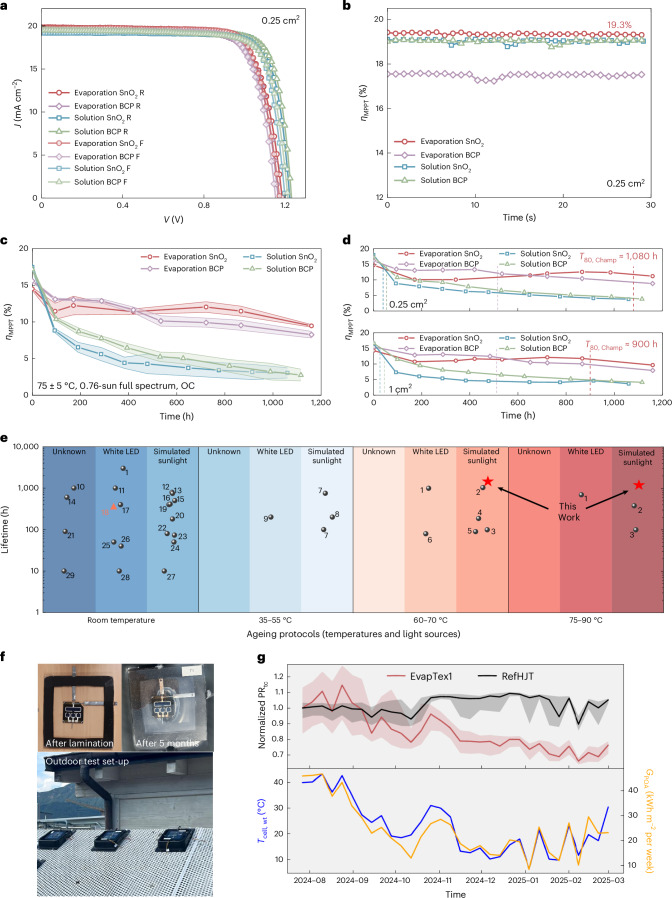
PSC characterization. **a**, *J–V* characteristics of our representative all-vacuum-deposited and solution-processed PSCs with an aperture size of 0.25 cm^2^ using an absorber composition of FA_0.83_Cs_0.17_Pb(I_0.75_Br_0.20_Cl_0.05_)_3_, with either atomic layer deposition (ALD) SnO_2_ or evaporated BCP buffer layers. R, reverse scan. F, forward scan. Anti-reflective foils were applied on the glass side of the cells to minimize the optical loss. Detailed PV metrics can be found in Supplementary Table 1. **b**, The *η*_MPPT_ of our representative evaporated or solution-processed 0.25-cm^2^ PSCs using FA_0.83_Cs_0.17_Pb(I_0.75_Br_0.20_Cl_0.05_)_3_. **c**, Evolution of the *η*_MPPT_ of encapsulated all-vacuum-deposited and solution-processed PSCs under the ISOS-L-2 protocol in ambient air (50–60% RH in the laboratory). The error bands represent the s.d. of independent cells (*n* = 5, 6, 8 and 8 for evaporation SnO_2_, evaporation BCP, solution SnO_2_ and solution BCP, respectively), and the centres represent the median values. **d**, Stability of individual champion cells from **c**. Dashed lines: time taken to drop to 80% of peak *η*_MPPT_ (*T*_80_, _Champ_). **e**, Literature stability data under different ageing protocols for passivation-free WBG PSCs with ideal bandgaps (1.65–1.72 eV) for perovskite-on-silicon tandems. Black spheres, PSCs containing solution-processed layers. Red triangle, all-vacuum-deposited PSCs. The references are listed in Supplementary Table 2. **f**, Photos of a laminated all-vacuum-deposited perovskite-on-silicon tandem before and after five months of outdoor ageing in Italy. **g**, Top panel: normalized PR_tc_ for an all-vacuum-deposited perovskite-on-silicon tandem cell (EvapTex1) and a reference HJT Si cell (RefHJT) under outdoor ageing conditions at MPPT for over eight months. The sample size is 1 for each condition. The error bands around PR_tc_ indicate the 10% to 90% percentile range weighted by power. Bottom panel: *T*_cell_,_wt_ (left-hand axis) and *G*_POA_ (right-hand axis) over the entire ageing period.

Our 0.25-cm^2^ evaporated cells with a SnO_2_ ‘buffer layer’ exhibit open-circuit voltage (*V*_OC_) up to 1.20 V (Fig. 3a,b and Supplementary Table 1), and excellent *η*_MPPT_ of 19.3% (Supplementary Fig. 13), certified as 18.35% (Supplementary Figs. 14 and 15). Our 1-cm^2^ evaporated cells (Supplementary Figs. 16 and 17 and Supplementary Table 1) demonstrate a minimum performance deficit (*η*_MPPT_ = 18.5%) compared with small-area cells, indicating high film uniformity, promising for large-area deposition. These efficiencies are among the highest reported for all-vacuum-deposited WBG PSCs (Supplementary Fig. 18), and are certified. A detailed *V*_OC_ loss analysis is given in Supplementary Note 5 and Supplementary Fig. 19. The external quantum efficiency (EQE)-derived PV bandgaps (*E*_g_^PV^) for the evaporated and solution-processed PSCs are 1.67 and 1.685 eV, respectively (Supplementary Figs. 20 and 21). Several unencapsulated evaporated cells demonstrate long ‘shelf lifetimes’, maintaining their peak performance (*η*_MPPT_ $\approx$ 18%) after ~20,000 h of storage under N_2_ (>2 yr) (Supplementary Fig. 22).

The rarely reported stability of WBG PSCs under harsh conditions, and the much shorter reported operational lifetime of all-vacuum-deposited PSCs^39,41^ relative to solution-processed counterparts, urge accelerated indoor stability measurements mimicking the real solar spectrum and elevated temperature to reliably predict field lifetime (Supplementary Note 6)^57–59^. Therefore, we conduct an ageing study on complete PSCs under the stringent ISOS-L-2 protocol combining light (full-spectrum simulated sunlight (0.76 sun) with no UV filter, Supplementary Fig. 23) and heat stressors (75 ± 5 °C) at OC (Supplementary Fig. 24). Our evaporated PSCs retain 80% of peak performance (*T*_80_) after ~840 and 330 h for the SnO_2_- and bathocuproine (BCP)-based devices, respectively, representing outstanding stability for WBG PSCs (Fig. 3c and Supplementary Figs. 25–29). Of particular note, our champion 0.25-cm^2^ evaporated cell reaches a *T*_80_ over 1,080 h (*T*_80_,_Champ_ = 1,080 h) with no change in visual appearance (Fig. 3d and Supplementary Fig. 24b). Similar stability (*T*_80,Champ_ = 900 h) is realized on a 1-cm^2^ champion cell. In contrast, the solution-processed cells only achieve *T*_80_ < 80 h, similar to what we previously observed^60^. For a fair comparison of the intrinsic stability of the co-evaporated and solution-processed cells, we introduced no additional passivation or additives in our solution-processed cells^60,61^. Comparing with the literature stability data for WBG PSCs (*E*_g_ = 1.65–1.72 eV, Fig. 3e), our all-vacuum-deposited WBG PSCs achieve operational stability on par with the state-of-the-art solution-processed PSCs^29–34,62^, setting a new benchmark for high-performance, stable, all-vacuum-deposited WBG PSCs.

We further verify the homogeneous and conformal deposition of our co-evaporated perovskites on industrial standard Czochralski silicon heterojunction (HJT) cells with micrometre-scale texture (Supplementary Figs. 30–32), achieving 24.3% *η*_MPPT_ for a 1-cm^2^ all-vacuum-deposited tandem, among the highest reported for all-vacuum-deposited stacks. By incorporating a solution-processed SAM HTL and passivation that we recently reported^7^, we further demonstrate a 1-cm^2^ tandem with 27.2% efficiency and 1.87 V *V*_OC_, representing state-of-the-art efficiency for evaporated perovskite-on-silicon tandems. We proceeded to conduct an outdoor test on laminated all-vacuum-deposited tandems (Methods) in Bolzano, Italy (Fig. 3f). During the first 3 months from August to October, where the sunlight and ambient temperature are the highest of the ageing period, the evaporated tandem shows stability on par with a reference commercial HJT Si cell, with a negligible loss in the temperature-corrected performance ratio (PR_tc_) (Fig. 3g)^63^. The PR_tc_ shows how well the device performs compared with standard testing conditions, by comparing actual outdoor yield with the reference yield expected from its rated power, solar irradiance and cell temperature. After eight months of operation, the evaporated tandem still maintains ~80% of its initial PR_tc_, suggesting that substantial stability of our single-junction all-vacuum-deposited WBG PSC is readily transferable to perovskite-on-silicon tandems. We also plot the variation in daily average cell temperature weighted by power (*T*_cell,wt_, left-hand axis) and the daily sum of global plane-of-array irradiance (*G*_POA_, right-hand axis) over the ageing period (Fig. 3g). While both the reference HJT Si cell and co-evaporated tandem show improved performance (PR_tc_) on brighter days (higher *G*_POA_), the tandem device exhibits a distinct seasonal decline in PR_tc_ that tracks the overall decrease in *G*_POA_. Intriguingly, the tandem’s sensitivity to irradiance increases with ageing, a phenomenon we attribute to progressively longer daily ‘light-soaking’ periods required to reach full performance. This correlation and its underlying origin, which lie beyond the scope of this study, will be investigated in more detail in follow-up work.

## Unveiling degradation via operando hyperspectral microscopy

**Fig. 4:**
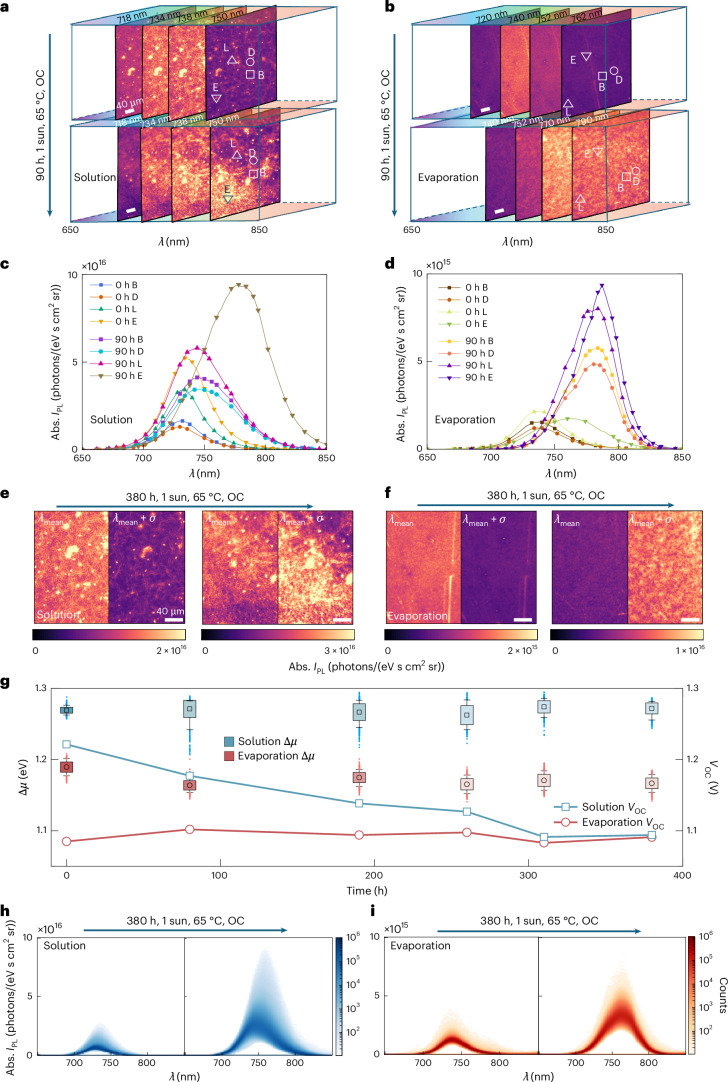
Operando hyperspectral imaging analysis on aged cells. **a**,**b**, The hyperspectral data cubes at different selected wavelengths for solution-processed (**a**) and co-evaporated (**b**) PSCs before and after 90 h of ageing under the ISOS-L-2 protocol (OC, 65 ± 5 °C, 1-sun full-spectrum illumination). Regions with different PL features on the mappings are marked with various symbols: square, bright wrinkle area (B); circle, dark wrinkle area (D); triangle and inverted triangle, bright spots with extra redshifted PL spectra (L and E). Scale bar: 40 µm. **c**,**d**, PL spectra of the corresponding marked regions in solution-processed (**c**) and co-evaporated (**d**) cells as shown in **a**,**b**, respectively. **e**,**f**, Hyperspectral data cube analysis to reflect the severity of redshifts in *λ*_mean_ for solution-processed (**e**) and co-evaporated (**f**) PSCs before and after 380 h of ageing under the ISOS-L-2 protocol. The *λ*_mean_ and redshift for solution-processed and co-evaporated cells are 734, 16 nm and 740, 30 nm, respectively. Scale bar: 40 µm. **g**, The evolution of mean Δ*µ* (left-hand axis) and real device *V*_OC_ (right-hand axis) for solution-processed and co-evaporated PSCs over 380 h ageing under the ISOS-L-2 protocol. The central square or circle in a box plot shows the mean of 1048,576 points over the entire Δ*µ* mapping area (1024 × 1024 pixels, Supplementary Fig. 38), indicating the central tendency of the data. The box length reflects data spread, while the whiskers mark the s.d. **h**,**i**, Density plots as a function of *λ* and absolute *I*_PL_ for both solution-processed (**h**) and co-evaporated cells (**i**) before and after 380 h of ageing under the ISOS-L-2 protocol.

To gain deeper insight into the factors driving degradation in WBG PSCs, we develop an operando hyperspectral imaging technique to monitor their absolutely calibrated photoluminescence (PL) properties after subjecting the cells to accelerated ageing under the ISOS-L-2 protocol (OC, 65 ± 5 °C, 1-sun full-spectrum illumination, 80–90% RH in the laboratory). Our set-up (Supplementary Fig. 33) emulates the illumination experienced by the full WBG device stack (Fig. 1d) using a 532-nm laser with an absorbed photon flux in the perovskite absorber equivalent to 171 mW cm^−2^ AM1.5G irradiance (1.71 suns). Here we compare our co-evaporated single-junction PSCs with solution-processed PSCs fabricated in the same way as aforementioned^60^. Supplementary Fig. 34 shows absolute PL intensity (*I*_PL_) taken for solution-processed and co-evaporated WBG cells with different objective magnifications at two selected wavelengths, 734 nm and 740 nm, respectively, corresponding to their mean peak emission wavelengths (*λ*_mean_), as identified in the hyperspectral data cube (Supplementary Videos 1–4). The *I*_PL_ map of solution-processed cells reveals a network of micrometre-scale ‘wrinkles’ dotted with discrete bright spots (Fig. 4a,c). Spectral slices identify three emissive populations: (1) bright wrinkles (square), which emit more strongly at ~739 nm, (2) dark wrinkles (circle), which are dimmer and blueshift to 726 nm, and (3) bright spots on the bright wrinkles (triangle and inverted triangle), which show the highest intensity and a further redshift to 740 nm. In contrast, co-evaporated perovskites display no wrinkle topography or bright spots, but their uniform *I*_PL_ is about an order of magnitude lower in intensity (Fig. 4b,d).

We use the quasi-Fermi level splitting (Δ*µ*) as a proxy for *I*_PL_ heterogeneity, since it reflects carrier populations, scales logarithmically with radiative versus non-radiative recombination and sets the upper limit of *V*_OC_ in a solar cell^64^. To extract Δ*µ* from the hyperspectral data cube, we employ a semi-analytical relation reported by Nayak et al.^64^, which couples the *I*_PL_ map with the *E*_g_^PV^ (Supplementary Note 7 and Supplementary Figs. 35–37). Fourier-transform photocurrent spectroscopy (FTPS) shows that Urbach energies stay at ~16–17 meV for 160 min of illumination (Supplementary Figs. 36 and 37). Consistently, the PL *λ*_mean_ shifts by less than ±5 nm (Fig. 4e,f), equivalent to a <10-meV change in *E*_g_^PV^ (Supplementary Fig. 20). Thus, the early-ageing PL redshifts we observed are attributable to genuine bandgap changes (for example, halide segregation) rather than tail-state emission. The mean Δ*µ* values for co-evaporated and solution-processed perovskites remain relatively steady, from 1.189 ± 0.005 and 1.269 ± 0.007 eV to 1.167 ± 0.003 and 1.272 ± 0.008 eV, respectively, upon ageing (Fig. 4g and Supplementary Fig. 38, errors derived from s.d.). The relatively small losses in Δ*µ* match the *V*_OC_ of the aged co-evaporated cell, but not the solution-processed one, since the latter appears to have a substantial deterioration in *V*_OC_, yet the Δ*µ* increased over the same ageing period (Supplementary Figs. 39 and 40).

To probe the Δ*µ*–*V*_OC_ mismatch, we analyse hyperspectral PL cubes (Fig. 4e,f). *σ* represents the s.d. of the spectral distribution at 0 h and serves as a fixed reference to gauge how much the spectral peak at later time points has redshifted relative to the initial *λ*_mean_, using *λ*_mean_ + *σ* as the threshold. In solution-processed perovskites, the wrinkle contrast gradually fades (Fig. 4e and Supplementary Fig. 41), as halide segregation propagates from bright wrinkles into darker regions, reshaping the local *I*_PL_ landscape. By contrast, the *λ*_mean_ in co-evaporated perovskites redshifts from ∼740 nm to ∼770 nm during ageing (Fig. 4f), consistent with iodide-rich phase segregation^65,66^. Yet short-circuit (SC) current density (*J*_SC_) remains nearly constant (Supplementary Fig. 40c), implying that the new I-rich domains do not inhibit carriers from reaching the contacts (Supplementary Fig. 42), consistent with previous spectroscopic studies on solution-processed films^13^.

To visualize the evolution of PL heterogeneity, we plot two-dimensional (2D) density heat-maps of absolute *I*_PL_ versus *λ* (Fig. 4h,i). The solution-processed cells develop a broad, bimodal distribution: a high-density band appears near 750 nm, and a secondary tail extends to ~770 nm with elevated *I*_PL_, signalling increasingly heterogeneous composition or defect-rich regions (Fig. 4h)^13,67,68^. In contrast, in co-evaporated cells, the emission remains relatively narrowly clustered, but does shift to longer wavelengths and with higher intensity, presenting iodine enrichment in a relatively homogeneous manner (Fig. 4i). Spectral dispersion in the solution-processed cells coincides with ~13% declines in device *V*_OC_ and *J*_SC_ and a ~7% drop in fill factor (Supplementary Figs. 39 and 40), indicating a trend consistent with incomplete carrier extraction under operating conditions, even where local Δ*μ* remains high. We further age and characterize co-evaporated PSCs without PbCl_2_ using operando hyperspectral imaging (Supplementary Figs. 43 and 44). They exhibit poorer initial PL uniformity, lower absolute *I*_PL_, indicating higher trap density, and more severe halide segregation during ageing as compared with the PbCl_2_-modified counterpart, coinciding with rapid performance loss (Supplementary Fig. 45). We thus postulate that adding a certain amount of PbCl_2_ facilitates the formation of highly crystalline and oriented grain structure in co-evaporated perovskites, whilst enhancing the PL homogeneity and resistance to halide segregation, leading to improved long-term operational stability (Supplementary Note 8)^69^.

Despite the pronounced PL heterogeneity, that is, bright wrinkles and redshifts from ~734 nm to ~750 nm (Fig. 4a,c,e), the solution-processed cell shows only a minor decline in average Δ*µ* (Fig. 4g). This mismatch implies that radiative metrics can stay high even when bulk transport, local phases or further changes to the charge transport layer contacts impede charge extraction under real operating conditions^61,70^. Locally elevated Δ*µ* may persist where carriers still recombine radiatively or contacts remain adequate, whereas the device-level *V*_OC_ may degrade because of energy-level shifts at the contacts, reduced selectivity to electrons or holes at the respective contacts or ‘parasitic recombination pathways’ current flow, as evidenced by the shunting behaviour and hysteresis developed in the measured device *J–V* curves of the aged solution-processed cells (Supplementary Fig. 39).

**Fig. 5:**
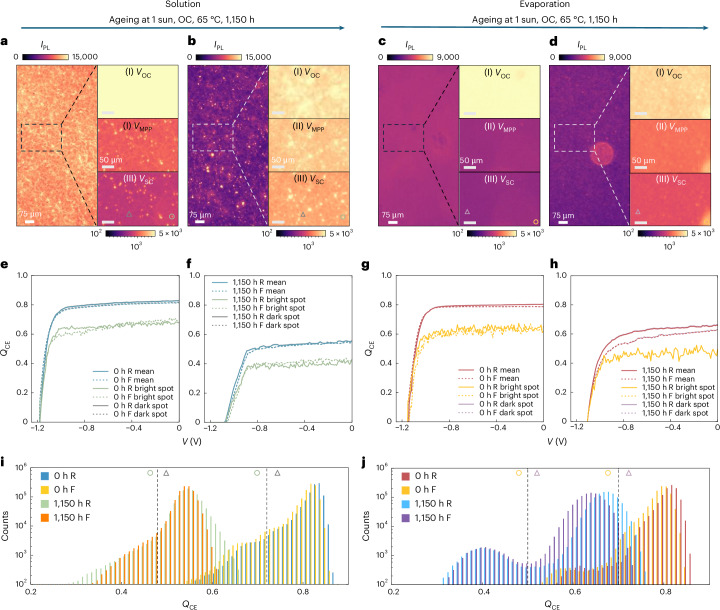
Charge-extraction pseudo-current–voltage characteristics of aged cells. **a**,**b**, Broadband PL maps measured at *V*_OC_ (left) for a region on a solution-processed PSC with SnO_2_ before (**a**) and after 1,150 h of ageing at the ISOS-L-2 protocol (OC, 65 ± 5 °C, 1-sun full spectrum) (**b**). A region is marked with a dashed square and plotted in a log scale to reveal the heterogeneous features at low *I*_PL_ at *V*_OC_ (I), *V*_MPP_ (II) and *V*_SC_ (III) (right). Green circle, bright spot with high *I*_PL_; grey triangle, dark spot with low *I*_PL_. **c**,**d**, Broadband PL maps measured following the same protocol as **a**,**b**, for a region on an all-vacuum-deposited PSC with SnO_2_ before (**c**) and after 1,150 h of ageing (**d**). Yellow circle, bright spot with high *I*_PL_; purple triangle, dark spot with low *I*_PL_. **e**,**f**, Ex-*JV* curves of the corresponding solution-processed cell in **a**,**b**. The area-average and dark-spot- and bright-spot-derived ex-*JV* curves are presented separately, where the area-average and dark-spot-derived ex-*JV* curves partly overlap with each other. The *Q*_CE_ is calculated using segmented ideality factors *n*_OC_ and *n*_SC_, which are assumed to be 1.16 and 1.26 for 0 h (**e**) and 1.62 and 1.42 for 1,150 h (**f**). **g**,**h**, Ex-*JV* curves of the corresponding all-vacuum-deposited cell in **c**,**d**. *Q*_CE_ is calculated using *n*_OC_ and *n*_SC_, which are assumed to be 1.04 and 1.11 for 0 h (**g**) and 1.011 and 1.012 for 1,150 h (**h**), respectively. **i**,**j**, Statistical distribution of the *Q*_CE_ over the same region on the corresponding solution-processed cell shown in **a**,**b** (**i**) and all-vacuum-deposited cell shown in **c**,**d** (**j**), before and after ageing. A dashed line and symbols are used to indicate the criteria for distinguishing the dark (grey triangle for solution-processed cell; purple triangle for evaporated cell) and bright spots (green circle for solution-processed cell; yellow circle for evaporated cell) on the PL maps at *V*_SC_.

To test whether the charge-extraction bottlenecks implied by the hyperspectral study translate into measurable performance losses, we recorded broadband *I*_PL_ under a series of bias voltages and normalized each signal to its OC value. Assuming that the fractional residual *I*_PL_(*V*) arises from charge recombination, then one minus this value should be proportional to the fraction of carriers extracted to the external circuit. Hence, this yields the charge-extraction quality (*Q*_CE_) and the corresponding extraction pseudo-*JV* (ex-*JV*) curves (Fig. 5a–h and Supplementary Note 9)^68^. Most ex-*JV* implementations assume a diode ideality factor (*n*) equal to unity^61,68,71^, but interface- and trap-assisted recombination can raise *n* above unity, especially near SC^72^. We adopt *n* = 1 as a first approximation (which assumes bimolecular (radiative) recombination, Supplementary Fig. 46) and then re-compute *Q*_CE_ with *n* = 1.4 and 2.0 to capture interface-driven or trap-mediated losses (Supplementary Figs. 47 and 48)^68,72^. This expanded analysis better reflects the true charge-extraction limits across operational biases and carrier densities, improving upon the simplified *n* = 1 baseline. Using a single *n* to construct ex-*JV* curves overestimates the drop in the measured device *J*_SC_ upon ageing (Supplementary Figs. 46–51); we thus introduce the bias-dependent ideality-factor (*n*(*V*)) treatment and segmented *n*(*V*) fitting to construct quantitative ex-*JV* curves, with details in Supplementary Note 9 and Supplementary Figs. 46–53^72–76^.

On the basis of previous works determining the ideality factors for perovskites of similar compositions^73–76^, we select the *n* at OC, *n*_OC_ = 1.04, 1.16, and at SC, *n*_SC_ = 1.11, 1.26 for fresh evaporated and solution-processed cells respectively, as a starting point, followed by screening of various combinations of *n* to fit the distinctive degradation behaviours of these cells. For solution-processed cells, we find that setting *n*_OC_ = 1.62 and *n*_SC_ = 1.42 at 1,150 h, and *n*_OC_ = 1.5, *n*_SC_ = 1.42 at 3,000 h, result in better fitted ex-*JV* curves with small differences (<4%) in extraction-derived *J*_SC_ (ex-*J*_SC_) and real *J*_SC_. Notably, if we just use *n* = 1, this discrepancy is >30% (Fig. 5e,f and Supplementary Fig. 50). The increases in *n*_OC_ and *n*_SC_ upon ageing are probably due to an increased fraction of Shockley–Reed–Hall recombination. This may partially arise from deteriorating interface as evidenced by the substantial drop in device *V*_OC_. In addition, the fast decay in *J*_SC_ indicates deterioration of the optoelectronic properties for the solar cell, which can also contribute to higher *n*. Similarly, using *n*_OC_ = 1.011, *n*_SC_ = 1.012 at 1,150 h and *n*_OC_ = 1.12, *n*_SC_ = 1.19 at 3,000 h substantially reduces the discrepancies between ex-*J*_SC_ and real *J*_SC_ for the evaporated cell to <4%, which is five times smaller than the mismatch (~20%) when *n* = 1 is used over all bias conditions (Fig. 5g,h and Supplementary Fig. 52). Interestingly, for evaporated cells *n*_OC_ is always smaller than *n*_SC_ throughout the ageing period, but for aged solution-processed cells (≥1,150 h) *n*_OC_ is greater than *n*_SC_. Such a difference may arise from the presence of severe interfacial loss or ion screening effect that screens internal potential at OC in the solution-processed cells, contributing to large *n*_OC_ (Supplementary Note 9)^70,77–79^.

In the fresh solution-processed cell, PL maps at *V*_OC_ already show bright and dark domains, signalling spatial heterogeneity (Fig. 5a). When a bias voltage is applied (e.g., maximum power point voltage (*V*_MPP_) and SC voltage (*V*_SC_)), isolated bright spots appear, corresponding to regions where charges are not efficiently extracted. Ageing enlarges and multiplies these spots and introduces ‘extra-bright’ sites that remain intense at all biases (Fig. 5b), indicating poor *Q*_CE_. Their spread lowers the area-averaged *Q*_CE_ by ~28% (Fig. 5b,e,f) and aligns with the sharp drop in *J*_SC_ and device performance (Supplementary Figs. 49 and 50). We infer that these pre-existing bright domains act as defect seeds; under light and heat stress they accelerate degradation of neighbouring regions, driving the overall *Q*_CE_ distribution to lower values and broader spread (Fig. 5i). After 3,000 h, bright spots cover nearly the entire map, coinciding with further *Q*_CE_ and PV performance losses (Supplementary Figs. 54a,c and 50).

On the other hand, the evaporated cell demonstrates more homogeneous PL distribution maps as well as consistently low *I*_PL_ at *V*_MPP_ and *V*_SC_ upon ageing (Fig. 5c,d), corresponding to a substantially slower drop (<14%) in the area-average *Q*_CE_ at SC (Fig. 5g,h), consistent with the more stable device performance (*T*_80_ of *η*_MPPT_ > 1,250 h, Supplementary Fig. 49). Interestingly, we observe a second population peak with lower *Q*_CE_ in the fresh and aged evaporated cell (Fig. 5j), which arises from a ‘defective’ area with slightly lower initial *I*_PL_ at OC and degrades faster than the other regions (Fig. 5c,d). However, the device performance demonstrates a strong tolerance to this defective area because most of the mapping areas retain high *Q*_CE_. The origin of this defective area is unclear, but a possible interpretation would be charge-extraction loss due to non-uniform charge transport layers.

In addition to a less considerable shift towards lower *Q*_CE_ in the co-evaporated perovskites, the population distribution roughly maintains its featured ‘two-peak’ shape after ageing (Fig. 5j). By contrast, the population distribution for solution-processed perovskites substantially deviates from its initial shape upon ageing (Fig. 5i). The current density losses that we observe in our solution-processed devices most likely originate from a drop in the *Q*_CE_, which we postulate to be induced by the photogeneration and spatial redistribution of mobile ionic species^70,80^. Our crystal-facet-directed co-evaporated perovskites effectively mitigate charge-extraction loss, thus highlighting the significance of forming highly oriented, highly crystalline, phase-pure perovskites for stable WBG PSCs and perovskite-on-silicon tandems.

## Discussion

Our findings demonstrate that our recipe for growing WBG (1.67-eV) perovskites via co-evaporation results in films with large grains of high crystallinity with ‘face-up’ orientation, high phase purity and homogeneity. The high material quality leads to good material and device stability under stringent heat and light stressors. Notably, our evaporated PSCs achieve excellent long-term stability on par with state-of-the-art solution-processed PSCs, which contain stabilizing additives. We further provide spectral insight into halide segregation and trap-mediated recombination, correlating microscopic luminescence features with macroscopic device performance, revealing that sustaining high charge carrier extraction efficiency is the root cause for the enhanced stability of our evaporated PSCs. Furthermore, we demonstrate all-vacuum-deposited perovskite-on-silicon tandems with robust operational longevity under outdoor conditions, highlighting the great promise of realizing efficient, durable PSCs via solvent-free fabrication protocols.

## Methods

### Precursor material preparation

ITO-coated glass substrates (15 Ω cm^−2^, Biotain), lead(II) iodide (PbI_2_, 99.999%, trace metal basis, Tokyo Chemical Industries), lead(II) bromide (PbBr_2_, 99.999%, Alfa-Aesar), lead(II) chloride (PbCl_2_, 99.999%, trace metal basis, Sigma Aldrich), FAI (>99.999%, Dynamo), caesium iodide (CsI, 99.9%, metal basis, Alfa-Aesar), Me-4PACz (>99.0%, Tokyo Chemical Industry), (2-(3,6-dimethoxy-9H-carbazol-9-yl)ethyl)phosphonic acid (MeO-2PACz, >99.0%, Tokyo Chemical Industry), (4-(3,6-diiodo-9H-carbazol-9-yl)butyl)phosphonic acid (I-4PACz, >99.0%, Lumtec), aluminium oxide nanoparticles (Al_2_O_3_, <50-nm particle size, 20 wt% in isopropanol, Sigma Aldrich), fullerene-C_60_ (C_60_, 99.5%, Sigma Aldrich), BCP (98%, Alfa-Aesar), Spiro-TTB (>99%, Sigma Aldrich), silver pellets (Ag, 99.999%, Kurt J. Lesker Company), gold pellets (Ag, 99.999%, Kurt J. Lesker Company), chromium bar (Cr, 99.999%, Kurt J. Lesker Company).

Ethanol (anhydrous, ≥99.9%, VWR), 2-propanol (IPA, anhydrous, 99.5%, Sigma Aldrich), *N*,*N*-dimethylformamide (DMF, anhydrous, 98%, Sigma Aldrich), dimethyl sulfoxide (DMSO, anhydrous, 98%, Sigma Aldrich), anisole (anhydrous, 99.7%, Sigma Aldrich), chlorobenzene (CB, anhydrous, 99.5%, Sigma Aldrich).

In this work, all the materials were used as received without further purification and weighed in a nitrogen-filled glovebox without exposure to light. The perovskite precursor solutions were stirred overnight in a nitrogen-filled glovebox and used without any further treatment.

### Film deposition and device fabrication

ITO–glass substrates were cleaned by sonication in Decon90/water (2 vol.%), scrubbed and rinsed. They were then sequentially sonicated in deionized water, acetone and IPA (15 min each), dried with N_2_ and treated with UV–ozone for 15–30 min before being transferred into a N_2_ glovebox.

#### Hole transport layers

For co-evaporated perovskites, a 5-nm Spiro-TTB HTL was thermally evaporated at 0.1 Å s^−^^1^ (chamber pressure ~1 × 10^−7^ mbar). For solution-processed perovskites, two HTLs were prepared: Spiro-TTB (5 mg ml^−^^1^ in CB) was spin-coated at 5,000 r.p.m. for 30 s and annealed at 120 °C for 10 min. Me-4PACz (0.3 mg ml^−^^1^ in EtOH) was spin-coated at 3,000 r.p.m. for 30 s and annealed at 100 °C for 10 min. Both solution-derived HTLs were hydrophobic. Therefore, an Al_2_O_3_ nanoparticle wetting layer (1:150 vol.% in IPA) was spin-coated at 2,000 r.p.m. for 20 s on HTLs before perovskite deposition.

#### Co-evaporated FA_0.83_Cs_0.17_Pb(I_*x*_Br_*y*_Cl_1__−*x*−*y*_)_3_ perovskites

To deposit the reference WBG perovskite FA_0.83_Cs_0.__17_Pb(I_0.80_Br_0.20_)_3_, five precursor sources (2× FAI, PbI_2_, CsI and PbBr_2_) were co-evaporated in a perovskite deposition chamber (details shown below) that is integrated as part of the National Thin-Film Cluster Facility at Oxford, under high vacuum (<1 × 10^−^^6^ mbar), using quartz-crystal microbalances for precise rate control. The deposition rates were set to 0.2 Å s^−^^1^ (FAI, 165 °C), 0.397 Å s^−^^1^ (PbI_2_, 310 °C), 0.074 Å s^−^^1^ (CsI, 435 °C) and 0.125 Å s^−^^1^ (PbBr_2_, 285 °C) to achieve the target stoichiometry, yielding a final thickness of 500 nm. In some preliminary experiments, 10-nm seed layers of PbCl_2_, CsCl or CsPbCl_3_ were deposited before the perovskite.

For the facet-engineered, seed-free growth, a six-source co-evaporation approach was adopted. Here, 5 mol% of PbI_2_ was replaced with PbCl_2_, leading to a nominal composition of FA_0__.__83_Cs_0__.__17_Pb(I_0__.__75_Br_0__.__20_Cl_0.05_)_3_, with PbI_2_ and PbCl_2_ deposited at 0.354 Å s^−^^1^ (300 °C) and 0.027 Å s^−^^1^ (335 °C), respectively. The bandgap was tuned between 1.65 and 1.72 eV by varying the PbBr_2_ fraction (*x* = 0.17–0.28) while keeping other rates unchanged. Before each run, crucibles were replenished to maintain consistent charge levels, and all depositions were performed on unheated substrates (~27 °C). Unless otherwise noted, as-deposited films were subsequently annealed at 135 °C for 30 min in ambient air (40–50% RH).

#### Solution-processed FA_0.83_Cs_0.17_Pb(I_0.75_Br_0.20_Cl_0.05_)_3_ perovskites

Precursor solutions (1.4 M) were prepared in a DMF:DMSO mixture (4:1 v/v) from stoichiometric amounts of PbI_2_, PbBr_2_, FAI, CsI and PbCl_2_ in a N_2_-filled glovebox. After full dissolution, 170 µl of the solution was dynamically dispensed onto a substrate spinning at 1,000 r.p.m. The spin-coating programme was accelerated to 5,000 r.p.m. over 5 s, held for 35 s and quenched with 325 µl of anisole 5 s before the end. The as-cast films were transferred to ambient air (40–50% RH) and annealed at 135 °C for 30 min. Once cooled, the substrates were returned to the glovebox for subsequent processing steps.

#### Electron-transport layers (ETLs)

C_60_ (20-nm) and BCP (5-nm) layers were deposited on top of the perovskite films via thermal evaporation under vacuum (~1 × 10^−7^ mbar) with active-area masks applied. For devices with SnO_2_ buffer layers, 20 nm of SnO_2_ was grown in the ALD chamber using tetrakis(dimethylamido)tin(IV) (TDMASn) and deionized water precursors with 140 pulses. The reactor temperature during pulsing was stabilized at 100 °C while TDMASn was kept at 75 °C.

#### Metal contacts

Silver (100 nm) was thermally evaporated through shadow masks (active area defined as 0.25 or 1 cm^2^) at an initial rate of 0.2 Å s^−1^ (ramped up to 1 Å s^−1^ in 20 min) under high vacuum (~10^−7^ mbar) in a thermal evaporator (Nano36, Kurt J. Lesker). The devices for stability tests used 3.5-nm Cr and 100-nm Au gold electrodes instead of silver.

#### Perovskite deposition chamber

The perovskite thin-film layer is fabricated in an Angstrom Engineering deposition chamber under high vacuum, achieved through a two-step process combining an Ebara EV-A10-2 dry pump and a CTI Cryo Torr 8F cryogenic pump. Chamber pressure is monitored in real time using an INFICON Gemini MPG500 gauge, and the system is vented with high-purity nitrogen (99.999%).

Inside the chamber, eight identical thermal evaporation sources (Luxel RADAK II type) are arranged to minimize cross-contamination, each equipped with a 10-cm^3^ alumina crucible and individually shielded. Source shutters are actuated by oil-free compressed dry air and controlled via software. Evaporation rates from each source are precisely measured using water-cooled quartz-crystal microbalances that are also shielded to prevent signal interference.

A rotatable substrate stage (0–30 r.p.m.) enables combinatorial deposition through a programmable shutter, allowing gradients in thickness and composition for high-throughput screening. The stage can be resistively heated to 400 °C with ±2.5 °C stability. System integrity is monitored using a Stanford Research Systems RGA-300 residual gas analyser, which detects impurities and interlocks the gate valve to prevent contamination of connected cluster chambers.

#### Sputter system

The sputtering system (Angstrom Engineering) is integrated into the cluster tool, enabling automated, mask-based deposition sequences for full PV device fabrication. Capable of coating substrates up to M2 size with 96.6% uniformity, the chamber is evacuated by an Ebara backing pump and a Pfeiffer turbo pump to a base pressure of 1 × 10^−8^ mbar. Process gases (Ar, N_2_, O_2_, CF₄ and H_2_) are supplied from BOC or a hydrogen generator and regulated through mass-flow controllers via the AERES software.

An indium zinc oxide (IZO, (In_2_O_3_)_90_(ZnO)_10_) target bonded to a copper backplate was used as a critical window/interlayer for tandem devices. The target was mounted at a tilt angle of 32° and a target-to-substrate distance of 25 mm to optimize uniformity. Before deposition, the target was plasma-cleaned for 180 s with the substrate shutter closed. IZO layers (100 nm) were sputtered at room temperature using a gas mixture of Ar (18.1 sccm) and O_2_ (0.3 sccm) under a process pressure of 3 × 10^−^^3^ mbar, with the substrate rotating at 10 r.p.m. Deposition parameters were 410 V, 289 mA and 118.4 W, yielding a rate of 1.14 Å s^−^^1^ over 175 s. After deposition, the substrate was transferred under high vacuum back to the load lock.

#### Evaporated perovskite-on-silicon tandem solar cell

Evaporated perovskite-on-silicon tandem solar cells were fabricated on industry-standard M2-sized n-type Czochralski silicon wafers. The silicon-bottom subcells were prepared using HJT technology: wafers were KOH-textured to form random pyramids (1–5 μm), cleaned with an ozone- and HF-based process and passivated with a final HF dip. Intrinsic and doped hydrogenated amorphous silicon (a-Si:H) layers were deposited by plasma-enhanced chemical vapour deposition. For the rear contact, 70 nm of ITO (97/3 wt% In_2_O_3_/SnO_2_) was sputtered; a 12-nm ITO layer was deposited on the front side to serve as the recombination junction. After laser dicing into 3 × 3 cm^2^ substrates, the wafers were annealed at 180 °C for 20 min in ambient air to heal sputter-induced damage.

Before perovskite deposition, substrates were treated with UV–ozone for 15 min. For devices with an evaporated HTL, 5 nm of Spiro-TTB was thermally evaporated. For tandems employing a solution-processed SAM HTL, a 1:1 mixture of MeO-2PACz and I-4PACz (each 0.25 mg ml^−^^1^ in IPA) was spin-coated at 4,000 r.p.m. and annealed at 110 °C. The 1.67-eV WBG perovskite absorber (FA_0.__83_Cs_0.__17_Pb(I_0.__75_Br_0.__20_Cl_0.__05_)_3_) was deposited via the six-source co-evaporation method described earlier, with the thickness increased to 800 nm to enhance light harvesting. After deposition, the stack was annealed at 135 °C for 30 min in ambient air. For SAM HTL devices, a post-deposition passivation layer (0.5 mg ethane-1,2-diammonium iodide (EDAI_2_)+ 0.25 mg phenethylammonium chloride (PEACl) in 1 ml of 3:1 IPA:CB) was applied^81–83^, followed by spin-coating and annealing at 100 °C for 3 min.

The electron-transport stack consists of 15 nm of thermally evaporated C_60_, 20 nm of ALD SnO_2_ and 70 nm of sputtered IZO as the semi-transparent top contact. Front metal contacts were formed by evaporating 250 nm of Ag through an active-area mask, followed by 700 nm of Ag fingers. The rear contact was completed with 200 nm of evaporated Ag. The device active area is 1 cm^2^.

### Film and device characterization

#### Ultraviolet–visible (UV–Vis) absorption spectroscopy

Absorbance spectra were measured with a Varian Cary 300 Bio UV–Vis spectrophotometer with a 50 × 50 mm^2^ reflective neutral-density filter with an optical density of 3.0 (made out of UV-fused silica). Tauc plot analysis was used to estimate the material bandgap assuming a direct bandgap.

#### X-ray diffraction

The one-dimensional XRD patterns were obtained with a Panalytical X’Pert Pro X-ray diffractometer with a Cu Kα_1_ (1.54060-Å) source.

#### Ex situ grazing-incidence wide-angle X-ray scattering

Ex situ GIWAXS measurements were performed on a Rigaku SmartLab diffractometer equipped with a HyPix-3000 2D detector and a rotating Cu Kα source (8.048 keV). The sample-to-detector distance was 65 mm, and incidence angles of 0.5° and 1° were used. The X-ray beam was shaped using a parallel-beam optic, a 0.5° in-plane collimator and a 0.1-mm incident slit. Each scan was acquired for 60 min. Detector images were integrated into *Q*-space and processed into azimuthally averaged one-dimensional profiles using custom scripts based on the pyFAI and pygix libraries. Variable-angle GIWAXS scans (*αᵢ* = 0.5°–1°) were acquired for 60 min each using parallel-beam optics, a 0.5° in-plane collimator, a 0.1-mm incident slit and a 10-mm length-limiting slit. Data were processed as described above.

#### In situ grazing-incidence wide-angle X-ray scattering

In situ GIWAXS measurements were performed at beamline I07 of the Diamond Light Source. Perovskite films were annealed on a temperature-controlled hot plate under a nitrogen atmosphere. A 10-keV synchrotron beam struck the sample at a grazing incidence angle of 0.5°, and scattering patterns were recorded with a Pilatus 2M detector (calibrated using silver behenate). Solution-processed films were spin-coated on site within a fume hood, while evaporated films were prepared in Oxford and transferred via a vacuum-sealed tube. All data (experiment SI39532) were integrated into *Q*-space and azimuthally processed using pyFAI and pygix.

#### Scanning electron microscopy

An FEI Quanta 600 FEG environmental scanning electron microscope was employed to investigate perovskite layer morphology. Accelerating voltages between 4 and 15 kV were employed for various analyses.

#### Nuclear magnetic resonance spectroscopy

A two-channel Bruker AVANCE III HD NanoBay 400-MHz instrument running TOPSPIN 3 equipped with a 5-mm *z*-gradient broadband/fluorine observation probe is used. The signal from deuterated ethanol solvent is used for reference.

#### Photoluminescence quantum efficiency (PLQE) measurement

PLQE measurements were acquired using a custom-built PLQE set-up in an integrating sphere. Samples were photoexcited using a 450-nm laser (laser power = 0.665 mW). The PL was collected using a high-resolution monochromator and hybrid photomultiplier detector assembly (PMA Hybrid 40, PicoQuant GmbH). The PLQE was extracted from the photon energy (*hf*) and photon numbers of the excitation and emission obtained from numerical integration using Python.

#### Time-of-flight secondary ion mass spectrometry (ToF-SIMS)

Perovskite samples for ToF-SIMS measurements were prepared following the same procedure as used for solar cells. Measurements were conducted using a hybrid SIMS instrument (IONTOF M6), which was equipped with a bismuth primary ion source and an O_2_^+^ sputter source to probe the positive atomic and/or fragment ions. The ToF-SIMS data were acquired over an area of 150 × 150 μm^2^ using a 30-keV Bi^+^ primary ion beam. This was followed by a sputtering process, with each cycle lasting for 3 s. During each sputtering cycle, a 400 × 400 μm^2^ area of the sample was bombarded with 1-keV O_2_^+^ ion beams in an interlaced mode. The sample holder, containing the samples, was sealed in a container filled with argon gas and was transferred into the instrument immediately before testing.

#### Capacitance–voltage (*C*–*V*) profiling

The *C–V* characteristics were taken at 20 kHz with AC perturbation of 20 mV using an LCR meter (E4980A, Keysight). The scan frequency was determined from the plateau region of the capacitance–frequency profile at zero bias. The cells were kept in the dark at room temperature to reach equilibrium. The Mott–Schottky plot was generated from the *C–V* profile data by calculating (*A*/*C*)^2^, where *A* is the cell unit area (0.25 cm^2^) and *C* is the capacitance. The electrically charged defect density profile *N*(*x*) = −2[d*C*(*x*)^−2^/d*V*]^−1^/*qε*_0_*ε*_r_*A*^2^, where *q* is the elementary charge, *ε*_0_ is the vacuum permittivity and *ε*_r_ is the relative permittivity of the material, can be calculated as a function of profiling distance *x* = *ε*_0_*ε*_r_*A*/*C* from the Mott–Schottky plot.

#### Characterization of solar cells

*J–V* and MPPT data were acquired in ambient air using a Keithley 2400 series source meter under simulated AM1.5G illumination (WAVELABS SINUS-220 simulator) and in the dark. The active area was defined by a black-anodized metal aperture (0.25 or 1.00 cm^2^) in a light-tight holder. Each device was characterized sequentially: first, *V*_OC_ was recorded after 6 s of steady-state illumination; then, reverse (OC to SC) and forward (SC to forward-bias) *J–V* scans were performed at 245 mV s^−^^1^; this was followed by 30 s of active MPPT using a gradient-descent algorithm to extract the stabilized power output (*η*_MPPT_); finally, *J*_SC_ was measured after 3 s under steady-state short-circuit conditions. The simulator intensity was calibrated before each measurement session using a KG3-filtered Si reference diode (Fraunhofer ISE) to match its certified 1-sun *J*_SC_. Additionally, the internal spectrometer of the solar simulator provided a real-time spectral check; the ratio of this internal reading to the value recorded during calibration yielded an equivalent irradiance factor (here 0.985–1.005 suns), which was applied to correct the reported power conversion efficiencies.

#### EQE measurement

EQE was measured using a custom system built around a Bruker Vertex 80v Fourier-transform interferometer. A 250-W quartz–tungsten halogen lamp provided the illumination, which was monochromated, mechanically chopped at 280 Hz and focused onto the masked device (0.25 or 1 cm^2^). The resulting AC photocurrent was converted to a voltage across a 50-Ω series resistor and recorded with a lock-in amplifier. The EQE spectrum was obtained by dividing the device’s photocurrent response by that of a calibrated silicon reference cell (Thorlabs FDS100-CAL).

#### Optical microscopy

Optical microscope images were taken on a Nikon Eclipse LV100ND microscope with Nikon TU Plan Fluor lenses (10×/0.30 A, 20×/0.45 A, 50×/0.60 B, 100×/0.90A). The images were taken with an attached Nikon digital camera D6.10.

#### Photo- and thermal stability test

PSCs designated for stability testing were first sputter-coated with a 300-nm SiO*ₓ* layer through an active-area mask to shield the device from the UV-curable encapsulant. Encapsulation was performed in a nitrogen-filled glovebox using a cover glass (28.0 × 21.5 × 1.1 mm^3^) and UV-activated adhesive (Eversolar AB-341). The adhesive was uniformly applied across the substrate and cured under UV light for 3 min. For accelerated ageing, encapsulated devices were placed in an Atlas SUNTEST CPS+ chamber equipped with a 1,500-W air-cooled xenon lamp, which provided full-spectrum AM1.5G-equivalent illumination at an irradiance of approximately 76.5 mW cm^−^^2^. No UV filter was employed (Supplementary Fig. 23a). All ageing tests were conducted under OC conditions, in accordance with the ISOS-L-2 protocol.

To monitor the actual device temperature during ageing, we used a black standard thermocouple supplied with the chamber (setpoint 85 °C) alongside custom ‘mock-device’ thermocouples. The latter were bonded to black-anodized aluminium foil attached to ITO-coated glass slides, replicating the optical and thermal properties of the test solar cells. These mock devices, positioned identically to the test cells, recorded a stabilized temperature of 75 ± 5 °C. We hence moderate our estimation of our cell temperature in the ageing boxes to be 75 ± 5 °C. We note that in our previous publications where we stated 85 °C ageing the temperature was recorded on the Atlas-supplied black temperature standard, and hence likely to be 75 ± 5 °C, rather than 85 °C. The chamber was air-cooled, and the laboratory ambient RH (at ~21 °C) varied between 50% and 60% throughout the tests. Periodically, devices were removed for *J–V* characterization using the standard measurement protocol described previously.

For stand-alone perovskite thin-film stability tests, a protective poly(methyl methacrylate) layer (10 mg ml^−^^1^) was spin-coated onto the films to prevent physical damage, and to act as a rudimentary encapsulation.

#### Outdoor stability test

The perovskite-on-silicon tandem cells were prepared at Oxford and sent to CEA, France, for lamination. The laminated cells were then sent to a test facility that was deployed at Eurac Research in Bolzano, Italy (coordinates: 46° 28′ 31.7″ N 11° 19′ 49.7″ E) that allows for the measurement of perovskite-on-silicon tandem devices. The samples were installed on a frame with a tilt of 30° and an orientation of 190° from the north. The set-up was designed to measure electrical parameters of up to 24 small-scale encapsulated PV cells, with voltages up to 2.0 V and currents up to 100 mA at resolutions of 1 mV and 2 mA respectively (Fig. 3f). It monitors current, voltage and power at the MPPT using micro-MPPTs from Ljubljana University at a time resolution of 1 min. PT100 sensors concurrently measure cell temperature at the outer backside. All data were collected using a Python script. In addition to the device measurements, we collected plane-of-array irradiance data using a pyranometer and two crystalline-silicon-based reference cells, and weather data including ambient temperature, RH and precipitation intensity and type.

#### Hyperspectral operando microscopy characterization

Hyperspectral microscopy was performed using a Photon etc. IMA-VIS inverted system with 5× (Olympus MPLFLN 5×) and 20× (Olympus LCPLFLN 20× LCD) semi-apochromat objectives. Samples were transferred from a nitrogen glovebox to a motorized stage for in situ PL mapping during ageing. A 532-nm continuous-wave laser was used for optical excitation. For the WBG sample used in characterization, the equivalent 1 sun is equal to 59 mW cm^−2^. For hyperspectral image acquisition, a laser power equivalent to 1.71 sun was used. For ex-*JV*, a laser power equivalent to 1 sun was used so that the measured *J*_SC_ under the microscope matched the *J*_SC_ of the device measured under our standard solar simulator. Illumination and detection were conducted through the same objective using a beam-splitter, enabling PL collection between 547 and 1,000 nm.

For spectral mapping, emitted light was dispersed by a volume Bragg grating with a spectral step of 2 nm over the range 650–850 nm; broadband mapping was acquired without the grating. A cooled Hamamatsu ORCA-Flash4.0 V3 camera (2,048 × 2,048 pixels, maintained at ~10 °C) recorded the signal. Absolute photon counts were obtained via a two-step calibration procedure described previously^68^. The technique was applied to evaporated and solution-processed perovskite films with different electron-transport layers, and during accelerated ageing experiments.

#### FTPS measurement

FTPS was performed using a Bruker Vertex 80v interferometer, an AM1.5-filtered xenon lamp and a trans-impedance amplifier. Unencapsulated PSCs (0.25 cm^2^) were held at OC in ambient air (~45% RH) and intermittently illuminated at 1-sun intensity. To acquire each EQE spectrum, the lamp power was briefly reduced to 0.1 sun to accommodate the amplifier’s gain limits. A long-pass filter (715 nm for evaporated devices, 780 nm for solution-processed ones) blocked above-bandgap light, extending the subgap sensitivity by four orders of magnitude. All spectra were calibrated against a certified silicon reference cell.

To extract the Urbach energy (*E*_U_), each EQE spectrum was fitted in the Urbach-tail spectral region, near the absorption edge, with the equation

1 $$\mathrm{EQE}=\exp \left(\frac{{hv}-{E}_{0}}{{E}_{{\rm{U}}}}\right)$$

where *E*_0_ is a constant.

#### Accelerated ageing for the sample used in hyperspectral microscopy characterization

For the hyperspectral microscopy ageing study, samples were aged under the ISOS-L-2 protocol: 65 ± 5 °C, 1-sun AM1.5G illumination (no UV filter, Supplementary Fig. 23b), OC bias and 80–90% RH. The tests were conducted in a Sunirad A-22 chamber (Lumartix SA) equipped with a plasma lamp. Encapsulated samples were placed face down (glass side up) and masked to expose only the 0.25- or 1-cm^2^ active area. During ageing, samples remained inside the chamber and were periodically removed for *J–V* characterization and hyperspectral microscopy.

#### Charge-extraction pseudo-*JV*

During ageing, PL mappings were acquired at 0, 1,150 and 3,000 h. For each measurement, the sample was mounted with its electrode facing upward and contacted via a probe connected to a Keithley 2400. A ×5 objective uniformly illuminated the entire 0.25-cm^2^ active area, and broadband PL was collected without spectral filtering. Mappings were recorded every second while the bias was stepped at 0.01 V s^−^^1^, collecting 140 consecutive maps in both forward and reverse scans from *V*_OC_ to 0 V (*V*_SC_). *Q*_CE_ was calculated per pixel (Supplementary Note 9). From these maps, area-averaged external *J–V* curves and spatially resolved *Q*_CE_ at *V*_OC_, *V*_MPP_ and *V*_SC_ were extracted. Bright and dark regions were distinguished by applying a threshold filter at *V*_SC_.

### Reporting summary

Further information on research design is available in the Nature Portfolio Reporting Summary linked to this article.

## Online content

Any methods, additional references, Nature Portfolio reporting summaries, source data, extended data, supplementary information, acknowledgements, peer review information; details of author contributions and competing interests; and statements of data and code availability are available at 10.1038/s41563-026-02494-w.

## Supplementary information

Supplementary Information Supplementary Tables 1 and 2, Notes 1–10 and Figs. 1–54.

Reporting Summary

Supplementary Video 1 Hyperspectral data cube of an all-vacuum-deposited PSC before ageing under ISOS-L-2 protocol.

Supplementary Video 2 Hyperspectral data cube of an all-vacuum-deposited PSC after 380 h of ageing under ISOS-L-2 protocol.

Supplementary Video 3 Hyperspectral data cube of a solution-processed PSC before ageing under ISOS-L-2 protocol.

Supplementary Video 4 Hyperspectral data cube of a solution-processed PSC after 380 h of ageing under ISOS-L-2 protocol.

## Data Availability

The complete data sets for all the data presented in this article and Supplementary Information, including the original measurement data, are available from https://ora.ox.ac.uk/. Further information is also available from the corresponding authors on request.
