# Supplementary material for: Crystal-facet-directed all-vacuum-deposited perovskite solar cells
Source: Nat Mater. 2026 Feb 23;25(6):999–1010. doi: 10.1038/s41563-026-02494-w (PMC13236600; doi:10.1038/s41563-026-02494-w)
Supplement: Supplementary file 1 — Supplementary Tables 1 and 2, Notes 1–10 and Figs. 1–54. [file 41563_2026_2494_MOESM1_ESM.pdf]

nature materials

Supplementary information https://doi.org/10.1038/s41563-026-02494-w

Crystal-facet-directed all-vacuum-deposited perovskite solar cells

In the format provided by the authors and unedited

Table of Contents **Page**

Table of Contents	Page
Supplementary Tables 1–2	2
Supplementary Notes 1–10	5
Supplementary Figures 1–54	21
References	75

Supplementary Table 1. PV metrics of the representative evaporated or solution-processed perovskite solar cells shown in **Fig. 3a** and **Supplementary Fig. 16**. Reverse: scan direction from reverse bias to short circuit. Forward: scan direction from short circuit to reverse bias. Steady-State: stabilised values obtained when the cells were held at V_{OC}, J_{SC}, and MPPT for 6 s, 6 s, and 30 s, respectively. The absolute values of V_{OC} are included in the table for better presentation.

0.25 cm² cells

Devices	Scan Direction	V_{OC} (V)	J_{SC} (mA cm^{-2})	FF (%)	PCE (%)
**Evaporation BCP**	Reverse	1.17	19.4	77.3	17.5
	Forward	1.15	19.4	78.3	17.5
	Steady-State	1.16	19.4	77.8	17.5
**Evaporation SnO_2**	Reverse	1.19	19.8	76.5	18.0
	Forward	1.17	19.8	78.8	18.3
	Steady-State	1.18	19.9	77.1	18.1
**Solution BCP**	Reverse	1.23	19.6	80.2	19.3
	Forward	1.21	19.5	78.2	18.5
	Steady-State	1.23	19.5	79.6	19.1
**Solution SnO_2**	Reverse	1.22	19.2	82.0	19.2
	Forward	1.20	19.2	81.5	18.8
	Steady-State	1.22	19.2	81.1	19.0

1 cm² cells

Devices	Scan Direction	V_{OC} (V)	J_{SC} (mA cm^{-2})	FF (%)	PCE (%)
**Evaporation BCP**	Reverse	1.16	19.6	74.5	16.9
	Forward	1.15	19.5	75.8	17.1
	Steady-State	1.16	19.5	74.7	16.9
**Evaporation SnO_2**	Reverse	1.17	20.5	76.0	18.2
	Forward	1.16	20.5	77.6	18.1
	Steady-State	1.16	20.5	76.1	18.1
**Solution BCP**	Reverse	1.23	19.4	79.2	18.9
	Forward	1.22	19.4	78.8	18.6
	Steady-State	1.23	19.4	78.8	18.8
**Solution SnO_2**	Reverse	1.23	19.2	79.6	18.9
	Forward	1.22	19.2	77.2	18.1
	Steady-State	1.23	19.3	78.4	18.6

Supplementary Table. 2. Literature stability data and ageing protocols on WBG perovskite solar cells with ideal bandgaps (1.65–1.72 eV) for perovskite-on-silicon tandem application. Data summarized in the table are from the passivation-free reference cells published in literature to compare with our passivation-free co-evaporated cells.

**Bandgap (eV)**	**Light source**	**Temperature (°C)**	**Stability**	**Reference**
**1.67**	**simulated sunlight (AM1.5G)**	**75 ± 5**	**OC, 0.76-sun, T_{80}=1080 h**	**This work**
**1.67**	**simulated sunlight (AM1.5G)**	**65 ± 5**	**OC, 1-sun, T_{80}=1250 h**	**This work**
1.69	white LED	85	MPPT at 1-sun, T_{90}=700 h	[1]
1.68	simulated sunlight	75 ± 5	OC, 0.76-sun, T_{80}=380 h	[2]
1.68	simulated sunlight	75 ± 5	OC, 0.76-sun, T_{80}=100 h	[3]
1.69	white LED	65	MPPT at 1-sun, T_{99}=1000 h	[1]
1.68	simulated sunlight	65	OC, 0.76-sun, T_{80}=1040 h	[2]
1.68	simulated sunlight	65	MPPT at 1-sun, T_{85}=187 h	[4]
1.68	simulated sunlight	65	OC, 1-sun, T_{80}=100 h	[5]
1.68	simulated sunlight	65	OC, 0.76-sun, T_{80}=90 h	[3]
1.66	white LED	65	MPPT at 1-sun in N_2, T_{80}=80 h	[6]
1.69	simulated sunlight	55	MPPT at 1-sun in N_2, T_{80}=100 h	[7]
1.65	simulated sunlight	50	MPPT at 1-sun, T_{80}=200 h	[8]
1.65	white LED	40	MPPT at 1-sun, T_{80}=200 h	[9]
1.69	simulated sunlight	35	MPPT at 1-sun in N_2, T_{80}=750 h	[7]
1.69	white LED	25	MPPT at 1-sun, T_{99}=3000 h	[1]
1.67	unknown	25	MPPT at 1-sun, T_{90}=1000 h	[10]
1.68	white LED	25	MPPT at 1-sun in N_2, T_{82}=1000 h	[11]
1.69	simulated sunlight	25	MPPT at 1-sun in N_2, T_{80}=790 h	[12]
1.67	simulated sunlight	25	MPPT at 1-sun in N_2, T_{80}=746 h	[13]
1.68	unknown	25	MPPT at 1-sun, T_{90}=600 h	[14]
1.69	simulated sunlight	25	MPPT at 1-sun, T_{80}=500 h	[15]
1.66	simulated sunlight	25	MPPT at 1-sun, T_{80}=400 h	[16]
1.68	white LED	25	MPPT at 1-sun, T_{65}=400 h	[17]

1.70	white LED	25	MPPT at 1-sun in N_2, T_{80}=340 h	18*
1.67	simulated sunlight	25	MPPT at 1-sun in N_2, T_{57}=410 h	19
1.68	simulated sunlight	25	OC, 1-sun, in N_2, T_{90}=180 h	20
1.685	unknown	25	MPPT at 1-sun, T_{85}=90 h	21
1.65	simulated sunlight	25	MPPT at 1-sun, T_{80}=80 h	22
1.67	simulated sunlight	25	MPPT at 1-sun, T_{90}=73.5 h	23
1.68	simulated sunlight	25	MPPT at 1-sun, T_{80}=50 h	24
1.68	white LED	25	MPPT at 1-sun, T_{80}=50 h	25
1.65	white LED	25	MPPT at 1-sun, T_{80}=40 h	26
1.67	simulated sunlight	25	MPPT at 1-sun, T_{90}=10 h	27
1.68	white LED	25	MPPT at 1-sun, T_{80}=10 h	28
1.68	unknown	25	MPPT at 1-sun, T_{80}=10 h	29

Notes:

*The WBG perovskite solar cell was fabricated with an all vacuum-deposited device stack.

MPPT: Maximum power point tracking

OC: Open-circuit condition

Supplementary Note 1: Optimisation of co-evaporation recipe

One effective approach is to employ "seed" layers to guide the crystal growth and improve perovskite film quality during VTE.[12,30] In particular, depositing a thin layer of CsCl improves crystallisation kinetics, reduces unwanted polymorphs, and enhances the final grain orientation.[12] Our early approach attempts to replicate the use of seed layers in our co-evaporation process to form ~1.67-eV WBG perovskites suitable for perovskite-on-silicon tandems.[12] Using our multiple-source co-evaporation system (**Fig. 1a**), we first deposit 10-nm of CsCl, $PbCl_2$, or $CsPbCl_3$ as seed layers separately to examine how it affects the subsequent growth of a 500-nm WBG perovskite, targeting the composition of $FA_{0.83}Cs_{0.17}Pb(I_{0.80}Br_{0.20})_3$ (**Methods**). In addition, we also explored co-depositing 6 VTE sources (2 × FAI, CsI, PbI_2, $PbBr_2$, and $PbCl_2$) directly onto the substrates (i.e., seed-free co-evaporation) to form a perovskite of $FA_{0.83}Cs_{0.17}Pb(I_{0.75}Br_{0.20}Cl_{0.05})_3$. Lohmann et al. demonstrated that co-evaporated $FA_{1-y}Cs_yPb(I_{1-x}Cl_x)_3$ (E_g = ~1.55 eV) prepared by substituting 20 mol% PbI_2 with $PbCl_2$ reduced defect density and improved optoelectronic properties,[31] albeit generating $CsPbCl_3$ impurities at > 20 mol% $PbCl_2$ ratio, compromising material stability.[31,32] Considering perovskites with high Br ratios exhibit stronger ability to incorporate Cl contents into lattice,[32,33] we co-evaporate only 5 mol% $PbCl_2$ into our seed-free deposition, leading to the best device performance and phase purity (**Supplementary Figs. 1**, **2**).

Supplementary Note 2: Compositional heterogeneity in seed-layer assisted perovskite evaporation

Previous studies have shown that the photo-unstable PbI_2 phase often arises from compositional inhomogeneity, compromising device stability.[34,35] Various impurity phases (e.g. PbI_2) have also been observed in a recent work studying seed-layer-assisted VTE of WBG perovskites,[12] where a perovskite with non-stoichiometric composition (e.g. $(FA_{0.94}Cs_{0.20})Pb(I_{0.94}Br_{0.11})_3$) was required to be deposited onto the seed layers to approach the targeted bandgap.[12] This is predominantly because the excess metal/organic cations and halides introduced by the seed layer can migrate into the final material to influence the bandgaps.[12] Consequently, the bandgap of the perovskite deposited on a seed layer cannot be straightforwardly controlled in an "in situ" fashion, thereby introducing an additional source of compositional inhomogeneity. Similarly, non-stoichiometric precursor ratios were generally required for preparing WBG perovskites with targeted bandgaps via evaporating molten alloy sources or the evaporation-solution hybrid deposition.[36,37] By contrast, our seed-free co-evaporation with $PbCl_2$ enables a fine-tuning of the material bandgaps from ~1.65 to 1.72 eV, with a step size ~0.01 eV (**Supplementary Fig. 3**).

Supplementary Note 3: Crystallographic and morphological differences between co-evaporated and solution-processed perovskites

One of the historic challenges with co-evaporated perovskites, in comparison to solution-processed films, is that they often have very small mis-orientated polycrystalline grains.[38] In order to compare our vapour deposited films, to archetypical solution-processed perovskite films, we have fabricated and characterised "anti-solvent quenched" spin-coated perovskite films, processed with a dimethylformamide/dimethyl sulfoxide (DMF/DMSO) solution, with the same nominal composition as our films here, 5 mol% of $PbCl_2$ was added into the perovskite precursor solution. For the as-cast solution-processed perovskite films we observe many more scattering peaks in the 2θ XRD pattern, more of an isotropic ring in the GIWAX patterns and a reduction in overall integrated scattering intensity by ~ 3 times of the evaporated perovskite films. This indicates more isotropic crystallographic orientation and lower overall crystallinity, which does not improve by thermal treatment (**Supplementary Figs. 5, 6** and **Fig. 1j**). When comparing the top view SEM images of co-evaporated to solution-processed films, we observe larger and what appear to be flatter grains from the co-evaporated films. When comparing the cross-sectional SEM images of these films, we observe large continuous grains traversing the entire thickness of the films for our co-evaporated perovskites, whereas the solution-processed films are composed of smaller grains, with multiple grain boundaries throughout the film thickness (**Supplementary Figs. 7, 8**). This demonstrates that our co-evaporation recipe, which only differs from convention by the compositional choice and a small inclusion of $PbCl_2$, overcomes these historical challenges. Notably, as we also show in the SEM image of a co-evaporated film of similar composition, deposited without $PbCl_2$ inclusion in **Supplementary Fig 7a**, it is the presence of $PbCl_2$ which is responsible for the improved grain morphology.

Supplementary Note 4: Origin of improved material stability of evaporated perovskites

A possible origin for the poor stability of solution-processed perovskite films can be entrapped highly coordinating solvent residues, such as DMF and DMSO. They have been shown detrimental to perovskite stability under light and heat,[39,40] which becomes a more severe issue in large-area fabrication.[41] We find signals corresponding to these solvent residues in ^{1}H liquid nuclear magnetic resonance (NMR) spectra of our solution-processed perovskites (**Supplementary Fig. 10**), which could partly contribute to their instability.

To reveal the impact of improved facet orientation on the stability of our evaporated perovskites, we also aged bare (upon ITO substrates, with PMMA top coating) evaporated perovskite films without $PbCl_2$ using the aforementioned ageing and characterisation protocols. We observe the formation of pinholes and decolouration of perovskite films start after 48 hours of ageing in the evaporated perovskites with no $PbCl_2$ (**Supplementary Fig. 11a**). The drop in the XRD peak intensities and absorption band edges of the photoactive perovskite phases is slightly slower than the solution-processed perovskites, but significantly faster compared to our optimised co-evaporated perovskites with $PbCl_2$ (**Supplementary Figs. 11b–d**). Although eliminating degradation factors introduced by residual solvents, the evaporated perovskite films without $PbCl_2$ still demonstrate poorer stability compared to our optimised co-evaporated perovskite films. Hence, the good material stability may also be a consequence of the face-up orientation of (100) facets with high crystallinity. The (100) facet has been reported to be the most stable facet in perovskite crystals, resulting from more complete atomic layers with charge neutrality ideal for the formation of defect-free surface, as compared to other facets.[42] Hence, having such facets exposed to the external environment should be beneficial for the long-term stability of materials and devices under extreme conditions.[43] In addition, Liu and McMeekin et al. have recently demonstrated that solution-processed WBG perovskites with face-up oriented (100) facets are more stable than the randomly oriented (110) and (100) facets.[2,44] It is worth noting that the surface properties (e.g., morphology,[43,45] defect density,[45,46] defect types[45,47]) of perovskite thin films are strongly influenced by the exposed facet orientation. Zheng and co-workers reported that (100) facet orientation could facilitate charge carrier transport across the absorber material and enhance device performance.[48] Such well-defined facet orientation in our optimised evaporated perovskites demonstrates that facet engineering is also possible with vapour-deposited films.[43]

Supplementary Note 5: Open-circuit voltage loss analysis

To quantify the V_{OC} losses in our devices, we carried out photoluminescence quantum efficiency (PLQE) measurements on glass substrates to assess recombination in the isolated perovskite absorber, and a series of perovskite "half-stacks" on ITO and textured FTO substrates, respectively, to estimate the degree of interfacial recombination at each charge transport layer/perovskite interface (**Supplementary Fig. 19a**). From the PLQE results, we also calculate the quasi-Femi level splitting (QFLS) of the same samples and the associated non-radiative energy losses with respect to the theoretical limit of the perovskite absorber (**Supplementary Figs. 19b** and **c**).

The non-passivated neat perovskite film on glass achieves a QFLS of 1.30 eV, with only ~110 meV non-radiative losses compared to the radiative limit, implying the co-evaporated perovskites have good film quality and optoelectronic properties. For the perovskite films coated upon the HTL (Spiro-TTB) coated ITO substrates (i.e. glass/ITO/HTL/perovskite) that we use in the devices, the PLQE and QFLS are significantly reduced because of strong non-radiative recombination contributing to around 33 meV further loss. When these HTL/Perovskite "half-stacks" are further coated with C_{60} (i.e. glass/ITO/HTL/perovskite/ETL), we determine a further loss in QFLS of 30 meV. When we fabricate similar films on the rougher FTO substrates we observe further QFLS losses of around 50 meV. This further loss on FTO may arise due to increased surface area of the textured structure leading to more severe surface recombination, but it may also be due to the perovskite film growing slightly differently upon the different substrates.[49]

Furthermore, we also find a mismatch between the internal QFLS of the complete device stack and the external V_{OC} (~30 meV) of our optimized devices on ITO, which is consistent with previous work using WBG perovskites and the same ETL.[32,50] This phenomenon has been associated with energetic misalignment between WBG perovskites and C_{60}.[51] Therefore, the development of transport layers with better energy level alignment can be a promising future direction to fully exploit the V_{OC} potential of WBG perovskites and tandem devices.

To reduce the non-radiative recombination and interfacial losses at the perovskite/C_{60} top interface, effective passivation on perovskites can be a viable direction to explore. More specifically, the well-defined facet orientation in our optimized evaporated perovskites is suitable for facet-specific passivation and modifications to further suppress the V_{OC} deficit and boost the device efficiency, whilst the study of facet engineering on solution-processed perovskite films is largely hindered by their complex nature and rough surface morphology.[43] Our recent work has demonstrated that a mixture of $EDAI_2$ and PEAI molecules has more favourable binding with the (100) facet than the (110) facet, enabling exceptional passivation effects on 1.8 eV solution processed perovskites.[52] These organic ammonium salts are compatible with thermal evaporation. Hence, investigating the evaporation of these

family of materials containing the ammonium functional groups and their passivation abilities on facet orientated co-evaporated perovskites will be an interesting future direction.

Since Spiro-TTB also contributes to a large drop in the QFLS in the glass/ITO/Spiro-TTB/co-evaporated perovskite half stack, selection of alternative HTL with better energy level alignment with 1.67 eV perovskites and more effective hole extraction is also a promising direction to explore. Our tandem devices with different HTLs (**Supplementary Figs. 31 & 32**) already imply the potential significance of this approach. By replacing evaporated Spiro-TTB with solution-processed SAMs which are known to reduce interfacial recombination and improve charge extraction,[53] the V_{OC} of our tandem cell can be increased by ~120 mV (from 1.75 V to 1.87 V), highlighting the huge V_{OC} loss at the perovskite/HTL bottom interface. Therefore, another promising future direction is the development of SAM molecules that are compatible with thermal evaporation and optimization of their mixing ratios, processing conditions and film thicknesses, to realize a compact HTL layer with excellent hole extraction and well-aligned energy levels with our perovskites. By synergistically adopting these two approaches, we believe that all vacuum-deposited WBG PSCs with higher V_{OC} and PCE will be achieved.

Supplementary Note 6: Significance of accelerated indoor ageing on evaporated perovskite solar cells

Combined light and heat stressors are the most challenging conditions for PSCs to operate in outdoor conditions,[54,55] especially for the WBG perovskites and their multi-junction cells. The operational stability of WBG PSCs under harsh conditions is unfortunately not-often reported. In most cases, device stability has been tested under heat or light separately rather than combined, which is much milder than the ISOS-L-2 conditions.[54–57] Moreover, the reported operational lifetime of evaporated PSCs to date, has been much shorter relative to the solution-processed counterparts.[36,38] To test the operational stability of our co-evaporated WBG PSCs, we age encapsulated cells under the ISOS-L-2 protocol: full-spectrum simulated sunlight (0.76 sun), 75 ± 5 °C, OC (**Supplementary Fig. 24**). The devices are taken out of the ageing chamber, allowed to cool to room temperature and measure at various time intervals in ambient air (50~60% RH in lab). Light intensity, spectrum, and temperature significantly impact cell stability.[58] To reliably predict field lifetime, indoor stability measurements should mimic the real solar spectrum.[58] Therefore, we perform all stability tests using full-spectrum simulated sunlight without a UV filter (**Supplementary Fig. 23**).

Supplementary Note 7. Operando hyperspectral imaging analysis

We use an IMA hyperspectral imaging system from Photon etc. (**Methods**) to record two-dimensional spatial maps (x, y) of emission spectra (**Supplementary Fig. 33**). For each pixel in a predefined field of view, the system collects a full PL spectrum $I_{PL}(E)$ over an energy range typically spanning 1.4–2.0 eV for metal-halide perovskites (**Fig. 4a-b**), though the range may be adjusted on the expected bandgap if the composition of a perovskite is known. The raw dataset can be visualised as a three-dimensional "cube," where two axes correspond to the spatial dimension (x, y) and the third axis is photon energy E. In practice:

$$I_{PL}(x, y, E) \text{ for } x = 1, \cdots, X;\ y = 1, \cdots, Y;\ E \in [E_{min}, E_{max}], \tag{1}$$

where X and Y represent the number of pixels in each spatial dimension, and E_{min} and E_{max} define the spectral range. For a semiconductor with finite quasi-Fermi level splitting ($\Delta\mu$), the emitted photon flux can be approximated by a generalized Planck emission formula,[59] If one neglects photon reabsorption within the film, one common form is:

$$I_{PL}(E) \propto \rho(E)\alpha(E)f_{BE}(E, \Delta\mu), \tag{2}$$

where:

- $\rho(E)$ is the photonic density of states, often taken as $\rho(E)$.
- $\alpha(E)$ is the absorption coefficient, which includes both above-bandgap absorption, i.e., $\alpha_0\sqrt{E - E_g}$ for a direct bandgap material, and sub-gap tail absorption.
- $f_{BE}(E, \Delta\mu)$ is the Bose–Einstein occupation factor, frequently approximated by a Boltzmann factor, i.e., $exp[(E - \Delta\mu)/(k_B T)]$ for energies near the band edge.

To account for an exponential band tail (Urbach tail) and a finite film thickness d, we may use:

$$\alpha(E) \approx \alpha_0\left(E - E_g\right)^{1/2} + A_{tail}exp\left(\frac{E_g - E}{E_U}\right), \tag{3}$$

with E_U denoting the Urbach energy. By fitting measured PL spectra $I_{PL}(E)$ to this model, we can extract the best-fit bandgap (E_g), E_U, and $\Delta\mu$. For instance, a least-squares approach can be used, in which the measured PL is compared pixel-by-pixel (or for an average surveyed region) to Supplementary Eq. 2 incorporating the absorption model of Supplementary Eq. 3. Systematic uncertainties in film thickness or local carrier density can make the exact fit challenging, reinforcing the need for reliable references from device EQE or reflection/transmission spectra.

Although one could, in principle, perform a full spectral fit at each pixel (or for selected regions) to construct $\Delta\mu$, this approach faces several practical challenges:

1. ***Separating sub-bandgap and band-edge emission:*** If the perovskite film exhibits multiple emissive phases or tail states, the fitting routine must properly account for these sub-gap contributions (**Supplementary Fig. 35**). Failure to do so can lead to mismatches between the extracted "E_g" and actual halide composition.
2. ***Complex reabsorption or photon recycling:*** Even if reabsorption is nominally small, thicker films or local thickness variations can distort the measured PL spectrum, hence complicating the direct application of Supplementary Eq. (2).
3. ***Computation intensity and uncertainties:*** Conducting a sophisticated multi-parameter fit (E_g, E_U, $\Delta\mu$, …) for tens or hundreds of thousands of pixels in a single hyperspectral data cube can be computationally expensive and more prone to fitting ambiguities.

For these reasons, a simple parameter like an I_{PL}-weighted E_g that was proposed in some reports.[60] However, such an approach does not separate band-edge emission from any sub-gap or multi-phase contributions (**Supplementary Fig. 35**). Therefore, large mismatches can arise between the I_{PL}-weighted E_g and the actual stoichiometric composition (e.g., Br:Pb ratio) if lower-energy defects or phases contribute even moderately to the PL intensity.[60] In situations where local composition fluctuations (e.g., halide segregation in mixed-halide perovskites) cause multiple emissive features, direct weighting by I_{PL} can skew the bandgap estimate toward the lower-energy emission peak. Similarly, partial phase segregation can lead to a red-shift in the time-averaged PL. Detailed spectral fitting can resolve these contributions at the expense of computational complexity.

Given that our tested samples are complete PV devices (not just bare films), and we ultimately care about the device-relevant bandgap and quasi-Fermi splitting, we adopt a semi-empirical equation of the form:[61]

$$qV_{\mathrm{OC}} = 0.941E_g^{PV} - (0.181 + 0.0257|\ln(\eta_{PL})|), \tag{4}$$

where q, η_{PL}, and E_g^{PV} are the elementary charge, photoluminescence quantum yield, and PV bandgap extracted from EQE, respectively. This formula is grounded in prior works on radiative-limit analyses of perovskites and other photovoltaic materials and typically captures both the bandgap and non-radiative losses at open-circuit.[61] The factor 0.941 accounts for the typical absorption edge offset in real PV materials. The coefficient 0.0257 in $|\ln(\eta_{PL})|$ approximately corresponds to k_BT/q, where k_B and T are the Boltzmann constant and temperature (i.e., combined as thermal energy), respectively, at room temperature, linking the radiative–non-radiative balance to a standard thermodynamic factor. This expression, validated in the literature, provides a practical handle on how internal luminescence

efficiency (η_{PL}) translates to an open-circuit voltage penalty. As a result, we bypass the need for pixel-by-pixel spectral fits to obtain $\Delta\mu$ from the local PL emission shape with the following three advantages:

1. ***Full device context:*** Since the PL was measured on an operational stack rather than a bare film, the EQE-derived E_g^{PV} is more relevant to actual device operation.
2. ***Avoiding sub-gap artifacts:*** By using η_{PL}, sensitivity to partial sub-gap emissions that might distort a direct spectral fit is reduced.
3. ***Computational simplicity:*** The local $\Delta\mu$ can be quickly mapped by measuring η_{PL} at each pixel and plugging it into the semi-empirical expression, rather than performing multi-parameter spectral fits (**Supplementary Fig. 38**).

Nonetheless, when specific microscopic features (like grain boundaries, wrinkles, or secondary phases) warrant a more rigorous spectral deconvolution, it remains possible to do partial or region-of-interest fits. In that sense, the semi-empirical method greatly accelerates broad spatial surveys but can be augmented by targeted, high-resolution modelling if necessary.

A key concern is that sub-gap tail states can broaden and red-shift the PL peak, decoupling it from the E_g^{PV} and complicating their combined use in Supplementary Eq. 4. To quantify the extent of these tail states, we measured sub-gap absorption with Fourier-transform photocurrent spectroscopy (FTPS) on both solution-processed and evaporated WBG devices (**Supplementary Figs. 36, 37**). We measure consistently low Urbach energies of ~16–17 meV that stay unchanged during 160 min of continuous illumination, confirming negligible sub-gap tails. Such narrow tails yield correspondingly well-defined PL features in the hyperspectral maps (**Figs. 4a–d**). Furthermore, as shown in **Figs. 4e, f**, the λ_{mean} varies by less than ±5 nm, equivalent to a <10 meV change in E_g^{PV} (**Supplementary Fig. 20**).

Supplementary Note 8: Role of $PbCl_2$ in suppressing halide segregation in co-evaporated perovskites

To elucidate whether the presence of $PbCl_2$ during the growth contributes to suppressing halide segregation in our co-evaporated PSCs, we have measured operando hyperspectral imaging for co-evaporated perovskites without 5 mol% $PbCl_2$ (**Supplementary Figs. 43, 44**). The fresh cells, without $PbCl_2$, demonstrate poorer PL uniformity as evidenced by the high density of bright and dark spots in the hyperspectral data cube and a bimodal distribution in the density plot (**Supplementary Figs. 43a, 44a**). This indicates a less "compositionally-homogeneous" film from the start. In addition, the PL peak is broader, and the absolute I_{PL} is one order of magnitude lower than the co-evaporated perovskites with $PbCl_2$ (**Fig. 4d, Supplementary Fig. 43b**), suggesting higher trap densities in the initial mixed phase.[50,62] This can possibly be attributed to its poor crystallinity as shown in **Fig. 1c** and **Supplementary Fig. 5a**. Upon ageing under light at elevated temperature, the co-evaporated perovskites without $PbCl_2$ experience a larger redshift in λ_{mean} by nearly 60 nm (from ~730 nm to ~790 nm), as compared to the $PbCl_2$-modified counterpart, alongside with a significantly enhanced absolute I_{PL} by 100 times, indicating more severe halide segregation and funnelling of carriers into the iodide-rich domains, which then undergo faster radiative recombination within the lower-gap domains (**Supplementary Figs. 43, 44**). Hence, the presence of $PbCl_2$ during growth, appears to play a direct role in suppressing halide segregation. More importantly, the localized heterogeneous phases (i.e. bright and dark spots) in the co-evaporated perovskites without $PbCl_2$ rapidly grow in size and density in a similar manner to what we observe in the solution-processed perovskites (**Supplementary Fig. 43a, Figs. 4a, e**), leading to rapid performance losses during prolonged aging as more carrier trapping at localized defect regions (**Supplementary Fig. 45**). Overall, we postulate that the addition of a certain amount of $PbCl_2$ facilitates the formation of highly crystalline and oriented grain structure in co-evaporated perovskites, whilst enhancing the PL homogeneity and resistance to halide segregation, eventually leading to long-term operational stability in WBG PSCs. However, we cannot rule out an active role of the presence of Cl ions in enhancing halide homogeneity and suppressing halide segregation, yet the measurable improvement in crystallinity alone may explain these results. A recent work on co-evaporated WBG perovskites also highlights the close relevance between crystal quality/orientation and the device lifetime under light and heat,[1] consistent with our results.

Supplementary Note 9. Charge-extraction pseudo-current-voltage characteristics

A charge-extraction pseudo-JV (ex-JV) is constructed by measuring bias-dependent PL on a fully fabricated solar cell and interpreting how local luminescence changes with voltage as a proxy for current-voltage behaviour. Similar approaches have been undertaken in the past,[60,63,64] but here we expand upon those methodologies, by explicitly including a bias dependent ideality factor and using absolute PL efficiency. We first start from the diode equation,[65] which can be expressed as

$$J(V) = J_0\left(e^{\frac{qV}{nk_BT}} - 1\right) - J_{ph}, \tag{5}$$

where:

- $J(V)$ is the net current density at an applied voltage V,
- J_0 is the reverse saturation current density (associated with recombination in the dark),
- J_{ph} is the photogenerated current density (under illumination),
- n is the diode ideality factor (describing the dominant recombination mechanism).[66]

In the radiative limit, n = 1, but in real devices, non-radiative recombination, such as trap-assisted or interfacial pathways, can raise n towards 2 or higher. This deviation plays a critical role in how carrier extraction and recombination are associated with applied voltage. Therefore, ignoring a non-unity n might lead to misinterpretations of how PL evolves with voltage, especially away from open-circuit, as other factors start taking effect and do not allow radiative recombination to be fully counted for the evaluation of extraction. We note that the value of n is also not necessarily constant as a function of applied bias, and hence may vary considerably when operating a PV cell near V_{OC} versus J_{SC}, with conditions including:

- ***Close to V_{OC}:*** High carrier densities favour bimolecular or interface recombination (n ≈ 1 or 1.2-1.5).
- ***Near J_{SC}:*** Trap-assisted processes (Shockley-Read-Hall recombination) often dominate (n ≈ 2).
- ***Ions/transport layers:*** Ionic migration or transport layer inhomogeneities can also shift the local recombination landscape, causing n to vary with bias or time.

Moreover, the voltage dependence of recombination can be framed in terms of the quasi-Fermi level splitting (or chemical potential, $\Delta\mu$), which is the internal photovoltage of the device. This splitting dictates the respective recombination currents for a PV cell operating at short-circuit and open-circuit conditions. Specifically, we can express the recombination balance at these two points as:

$$J_{SC} = J_G - J_0 e^{\frac{\Delta\mu_{SC}}{nk_BT}}, \quad (6)$$

$$0 = J_G - J_0 e^{\frac{\Delta\mu_{OC}}{nk_BT}}, \quad (7)$$

where:

- J_G is the total generation current density (typically close to J_{ph} under steady-state illumination),
- $\Delta\mu_{SC}$ and $\Delta\mu_{OC}$ denote the quasi-Fermi level splitting at the short-circuit and open-circuit conditions, respectively.

To assess how efficiently photogenerated carriers are extracted as current under short-circuit, i.e., $J(V)$, we compare the luminescence, which reflects internal recombination, under the short-circuit ($PLQY_{SC}$), i.e., $J(V = 0) = J_{SC}$, and to the luminescence under open-circuit conditions ($PLQY_{OC}$), i.e., $J(V_{OC}) = 0$, regimes (**Fig. 5a–d**).[64] This comparison enables us to define a "charge extraction quality" metric, Q_{CE} = J_{SC}/J_G, based on the ratio of the PL quantum yield (*PLQY*) at different biases and further derive using Supplementary Eqs. 6 and 7 as

$$Q_{CE} = 1 - \left(\frac{PLQY_{SC}^{1/n_{SC}}}{PLQY_{OC}^{1/n_{OC}}}\right). \quad (8)$$

This formula effectively accounts for the difference in quasi-Fermi splits between short-circuit and open-circuit, scaled by an ideality factor that captures non-ideal recombination paths. We have used this Q_{CE} in the past, however, in previous work we kept *n*, constant between OC and SC. Others have used a similar equation, without the consideration of *n*, but referred to this as the "charge collection efficiency".[60] This latter approach would be valid for well-behaved PV devices with an ideality factor of 1 independent of bias conditions. For a range of different perovskite solar cells, we previously found correlation, but non-quantitative agreement between the charge collection quality metric and the charge extraction efficiency (as determined by real J_{SC} measurements).[63,64] As we show below, these differences can be well accounted for by allowing a variable *n* (**Supplementary Figs. 50–52**). As reasoned above, a variable *n* is more physical for perovskite solar cells.[67] For any given applied bias (*V*) between short-circuit and open-circuit, we can generalise Q_{CE}, as follows:

$$Q_{CE} = 1 - \left(\frac{PLQY(V)^{1/n(V)}}{PLQY_{OC}^{1/n_{OC}}}\right), \quad (9)$$

Supplementary Eqs. 8 and 9 capture how both radiative and non-radiative recombination evolve as a function of applied bias. In an ideal scenario with $n = 1$ throughout and purely radiative recombination, the PL at $V \sim 0$ (short-circuit) would be fully quenched, yielding $Q_{CE} = 1$. In practice, non-idealities cause incomplete quenching (i.e., finite PL at short-circuit) and bias-dependent trap activity, resulting in lower Q_{CE}, i.e., less than unity. Additionally, the exponent $1/n(V)$ ensures that bias-dependent recombination pathways, hence changes in quasi-Fermi level splitting and interface/bulk recombination, are properly accounted for when interpreting the extent of PL quenching. By performing these bias-dependent PL measurements spatially, one can construct an ex-*JV* map over the full or partial device area, highlighting regions with poor charge extraction, such as minimal PL quenching at SC, and, thus, revealing underlying morphological or compositional heterogeneities. This approach is particularly useful for identifying local defects, cracks, or partial delamination under stress.

Using a single ideality factor (n = 1, 1.4, or 2) to construct ex-*JV* curves (**Supplementary Figs. 46–48**) shows a steady decline in Q_{CE} during 3,000 hours of ISOS-L-2 ageing. Although this trend mirrors the overall performance loss, it overestimates the drop in the measured device J_{SC} (**Supplementary Figs. 49**), especially at higher n values (**Supplementary Figs. 50a–c**, **51a–c**). In reality, n can vary with bias or over time due to ion migration, transport layer heterogeneity, and trap-mediated/interfacial-driven recombination varying depending upon the carrier density and chemical potential within the device.[65,67] To capture the possible bias dependence of n, we segment the *J–V* curves into regions from OC to MPP, and from MPP to SC, and apply different ideality factors to the different sections (**Supplementary Fig. 51**).

Based on previous works determining the ideality factors for perovskites of similar compositions,[67–70] we select the ideality factor at OC, n_{OC} = 1.04, 1.16, and at SC, n_{SC} = 1.11, 1.26 for fresh evaporated and solution-processed cells respectively, as a starting point, followed by screening of various combinations of n to fit the distinctive degradation behaviours of these cells. Furthermore, we note that the overall density of electrically active defects, including trap states, are lower for the evaporated cells as compared with the solution-processed cells, as determined via capacitance-voltage (*C-V*) profiling (**Supplementary Fig. 53**).[71–73] This implies that we expect to determine an n closer to 1 for the evaporated cells. The fittings for n at other bias conditions ($n(V)$) between V_{OC} and SC voltage (V_{SC}) are separated into two parts: the first part is from V_{OC} to near maximum-power-point voltage (V_{MPP}), and the remaining section until V_{SC} is considered as the second part. We fit the first part with a series of n with a desired evenly distributed step size, either increase or decrease from n_{OC} to n_{SC}. The step size and total number of n used are determined manually to give the best coincidence of the ex-*JV* with the real *J–V* curves. For the second part, setting $n(V) = n_{SC}$ leads to good fittings with the real *J–V* curves (**Supplementary Figs. 50–52**). We estimated 100% internal quantum efficiency (IQE) from the detailed balance limit for 1.685-eV and 1.67-eV absorbers under AM1.5G, giving J_{SC} of ~ 22.5 and ~ 23 mA

cm^{-2}, respectively.[74] These values are used as fixed $Q_{CE} = 1$ references in the ex-*JV* analysis for solution-processed cells and evaporated cells.

Supplementary Note 10: Practical scalability and industrial feasibility of co-evaporation

In addition to high efficiency and operational stability, high throughput is also a preliminary requirement for commercial technology. In this work, we used six thermal evaporation sources and relatively slow deposition rates (total rate ~ 1 A/s) for our perovskite deposition to maintain the good film quality, which translates to 2.5 hours of duration for depositing 800 nm of perovskites for making tandem cells. However, such a long manufacturing process is far below the standard for high-throughput production.[75] Ideally, industrially viable deposition rates for perovskite absorber layers should be equal to or above 1000 nm/min.[75] Examples of successful and scalable thin-film manufacturing can be found in the established thin-film industry (e.g., cadmium telluride (CdTe)[76] or copper indium gallium selenide (CIGS) PV,[77] and organic light-emitting diodes (OLED)[78]), which is exclusively based on vapor-based techniques. Therefore, exploring strategies to deposit perovskite films at high deposition rates (> 17 nm/s) without sacrificing the optoelectronic properties requires great attention from the communities to bridge the gap between lab-scale research and industry production. We believe the fundamental material understanding we have gained in this work and the lessons we could effectively learn from the successful deployment of OLED will substantially advance thermally evaporated perovskites towards large-scale production.

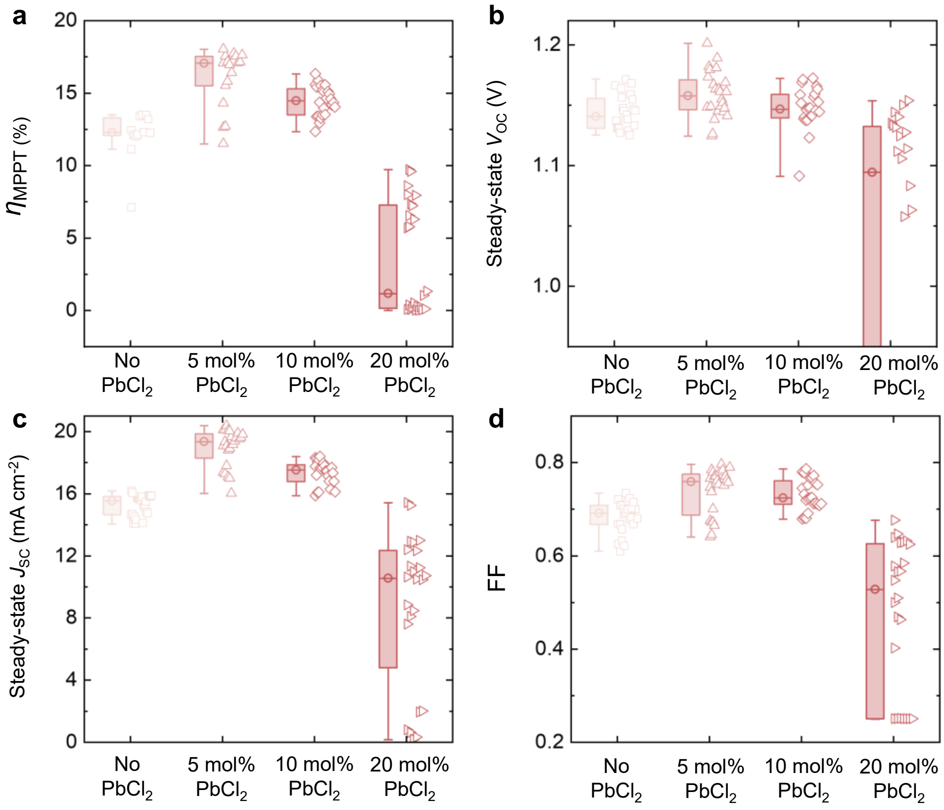

Supplementary Fig. 1. a–d, PV metrics of seed-free co-evaporated perovskite solar cells of $FA_{0.83}Cs_{0.17}Pb(I_{0.80-x}Br_{0.20}Cl_x)_3$ with various $PbCl_2$ concentrations (x = 0, 5, 10, and 20 mol%). The middle line in a box plot shows the median of 24 cells for each $PbCl_2$ molar concentration, indicating the central tendency of the data. The box length reflects data spread, while the 'whiskers' mark the maximum and minimum values of the normal range. The PV parameters include η_{MPPT} (a), steady-state V_{OC} (b), steady-state J_{SC} (c), and FF (d). Co-evaporation with a low concentration of $PbCl_2$ (5 mol%) improves the PV parameters. With further increase in $PbCl_2$ concentration (10 mol%), the device performance drops slightly, whilst too much $PbCl_2$ (≥ 20 mol%) results in significant performance losses. This trend is consistent with the appearance of PbI_2 impurity phase at low $PbCl_2$ concentrations (10 mol%), followed by the formation of undesired hexagonal δ-$FAPbI_3$ (2θ = 11.7° and 15.5°) and δ-$CsPbI_3$ (2θ = 24.1°) phases when over 20 mol% of $PbCl_2$ is co-evaporated as we determine via X-ray diffraction (XRD, **Supplementary Fig. 2a**). In the latter material, we also observe bright spheres on the surface and at the bottom interface as we determine via scanning electron microscopy (SEM, **Supplementary Figs. 2b, c**), which are consistent with PbX_2 crystals having formed in these films. We thus attribute the drops in V_{OC}, J_{SC} and FF to the trap-assisted recombination and reduced charge extraction caused by PbI_2 insulating phases and photo-inactive δ-$FAPbI_3$ and δ-$CsPbI_3$ phases.[79,80] Hence, 5 mol% $PbCl_2$ was determined as the optimal condition in further device fabrications due to improved phase purity and high device performance.

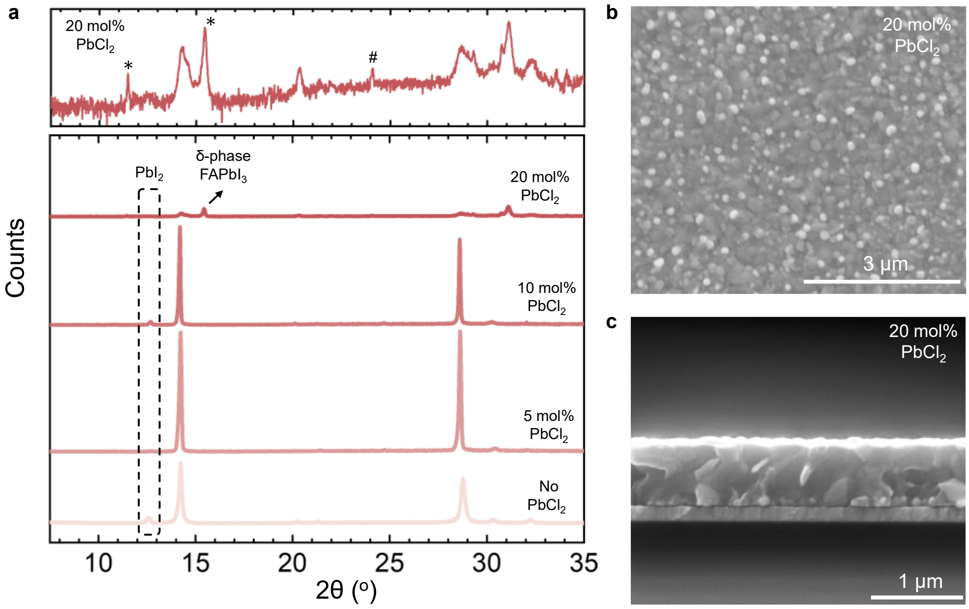

Supplementary Fig. 2. a, XRD patterns of seed-free co-evaporated perovskite films of $FA_{0.83}Cs_{0.17}Pb(I_{0.80-x}Br_{0.20}Cl_x)_3$ with various $PbCl_2$ concentrations (x = 0, 5, 10, and 20 mol%). The XRD of perovskites with 20 mol% $PbCl_2$ is plotted separately in log scale to reveal the minor impurity phases: δ-$CsPbI_3$ (#); δ-$FAPbI_3$ (*). One sample was measured for each condition. **b–c,** Top-view (b) and cross-sectional (c) SEM images of co-evaporated perovskite film with 20 mol% $PbCl_2$.

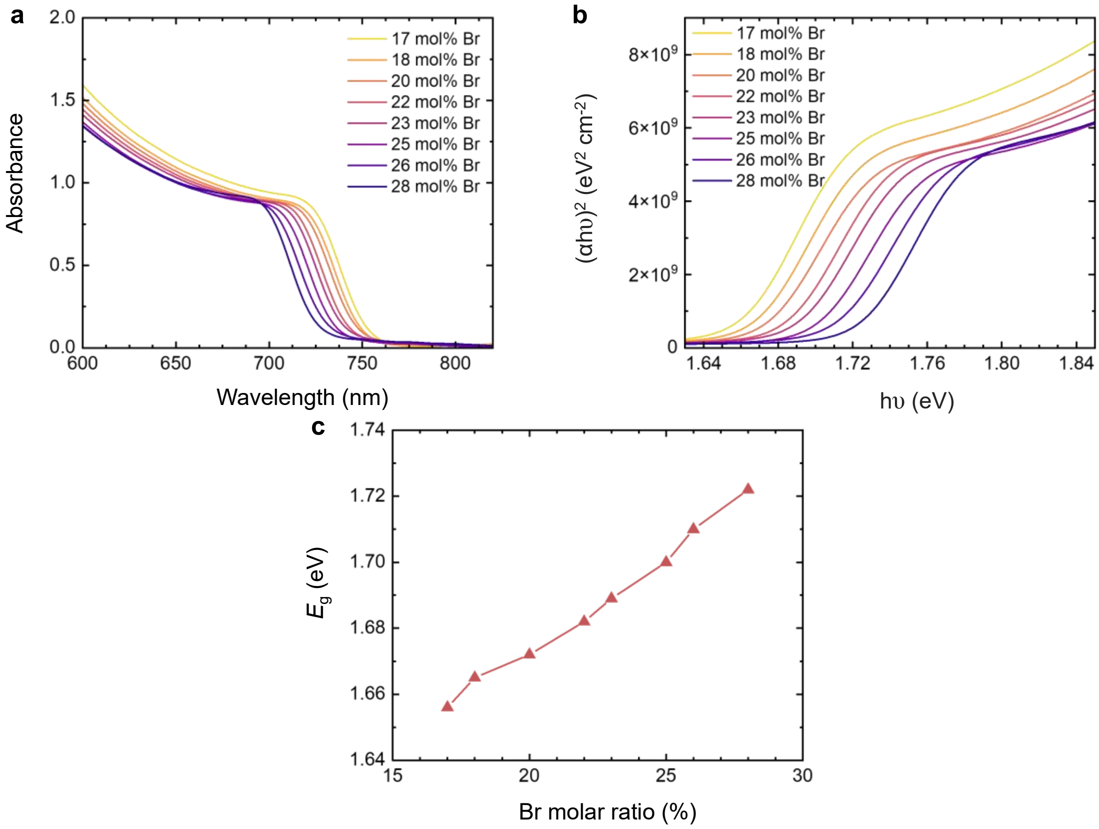

Supplementary Fig. 3. Bandgap tuning of crystal-facet-directed, seed-free co-evaporated WBG perovskites from 1.65 to 1.72 eV. a–b, UV-vis absorbance spectra (a) and the corresponding Tauc analysis (b) of crystal-facet-directed, seed-free co-evaporated perovskites with compositions of $FA_{0.83}Cs_{0.17}Pb(I_{0.95-x}Br_xCl_{0.05})_3$, with a range of Br molar ratio (x) with $17 \leq x \leq 28$ mol%. **c,** Optical bandgaps (E_g) extracted from the Tauc analysis of the corresponding perovskites with various Br molar ratios shown in (a).

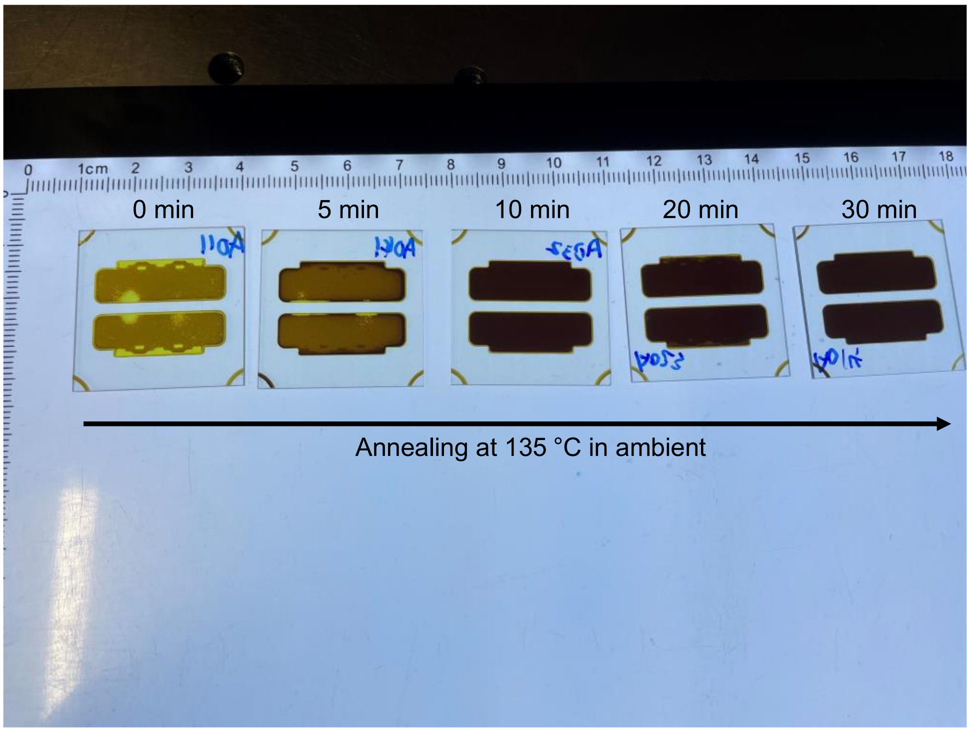

Supplementary Fig. 4. Photos of freshly prepared crystal-facet-directed, seed-free co-evaporated perovskite films of $FA_{0.83}Cs_{0.17}Pb(I_{0.75}Br_{0.20}Cl_{0.05})_3$ with various annealing times (from 0 min to 30 min) at 135 °C under ambient conditions (relative humidity in lab = 50–60%), as shown in **Fig. 1g**. The photos were taken immediately after conducting the XRD measurements on the films (~ 1.5 hour of exposure to ambient conditions).

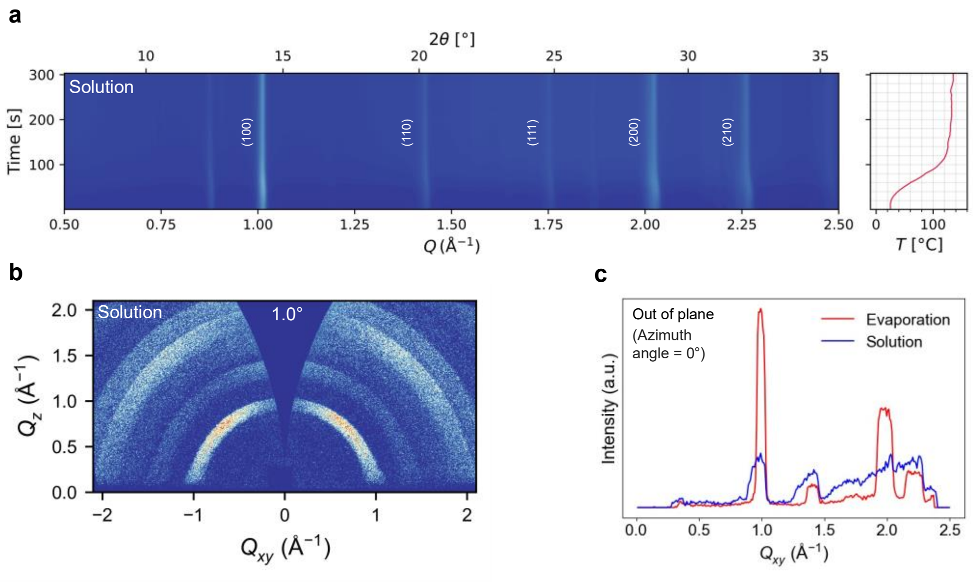

Supplementary Fig. 5. a, Time resolved GIWAXS pattern for the in situ annealing of an as-deposited solution-processed perovskite material of $FA_{0.83}Cs_{0.17}Pb(I_{0.75}Br_{0.20}Cl_{0.05})_3$ at 135 °C in air (relative humidity in lab ≈ 40–50%), which resembled the device fabrication conditions. **b,** Ex-situ 2D GIWAXS intensity mapping from an incident angle of 1.0° for an annealed solution-processed perovskite film of $FA_{0.83}Cs_{0.17}Pb(I_{0.75}Br_{0.20}Cl_{0.05})_3$ with a film thickness of 500 nm. **c,** Line cuts along the out-of-plane direction (Azimuthal angle = 0°) of the ex-situ 2D GIWAXS mappings shown in (b) and **Fig. 1i**.

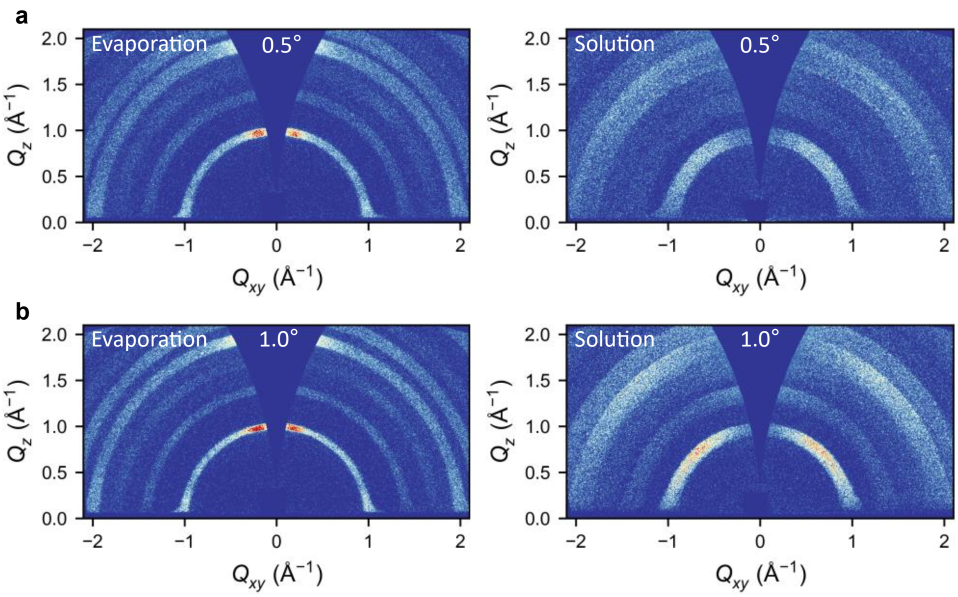

Supplementary Fig. 6. a–b, 2D GIWAXS intensity mappings from various incident angles: 0.5° (a) and 1.0 ° (b), for the evaporated and solution-processed perovskite thin films of $FA_{0.83}Cs_{0.17}Pb(I_{0.75}Br_{0.20}Cl_{0.05})_3$.

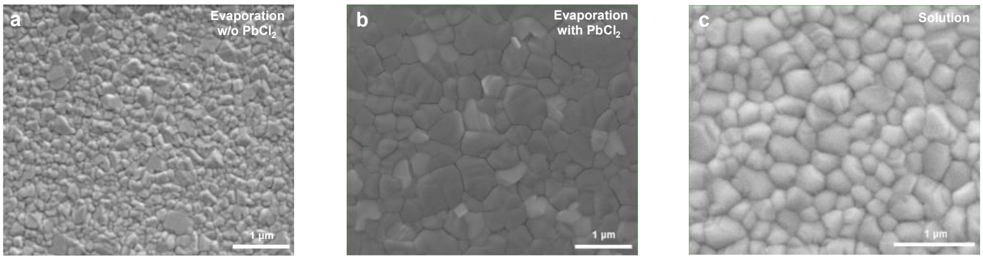

Supplementary Fig. 7. a–c, Top-view SEM images of the evaporated $FA_{0.83}Cs_{0.17}Pb(I_{0.80}Br_{0.20})_3$ (namely "Evaporation w/o $PbCl_2$", a), $FA_{0.83}Cs_{0.17}Pb(I_{0.75}Br_{0.20}Cl_{0.05})_3$ (namely "Evaporation with $PbCl_2$", b) and solution-processed $FA_{0.83}Cs_{0.17}Pb(I_{0.75}Br_{0.20}Cl_{0.05})_3$ (namely "Solution", c) perovskite thin films. Scale bars: 1 μm.

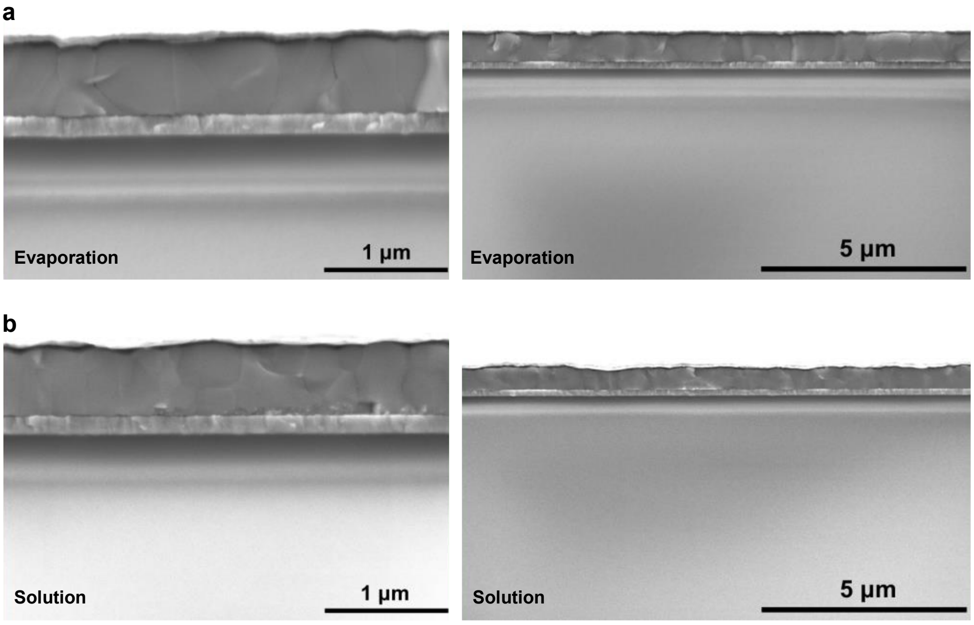

Supplementary Fig. 8. a–b, Cross-sectional SEM images of evaporated (a) and solution-processed (b) perovskite solar cells of $FA_{0.83}Cs_{0.17}Pb(I_{0.75}Br_{0.20}Cl_{0.05})_3$, with ALD SnO_2 buffer layer, same as the device architecture shown in **Fig. 1d**.

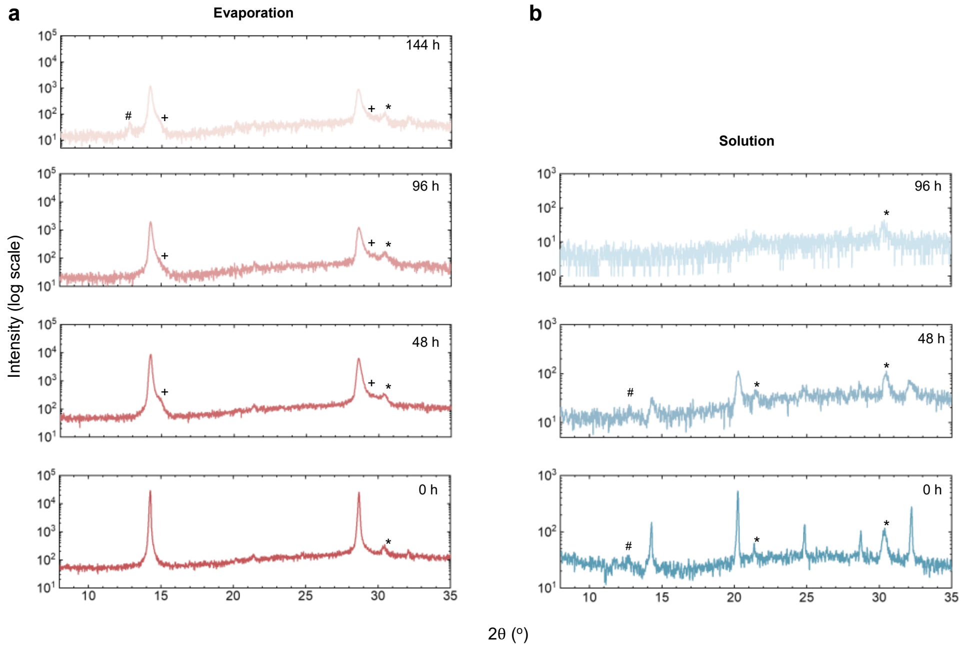

Supplementary Fig. 9. a–b, Evolution of the XRD patterns of unencapsulated evaporated (a) and solution-processed (b) perovskite films of $FA_{0.83}Cs_{0.17}Pb(I_{0.75}Br_{0.20}Cl_{0.05})_3$ as shown in **Fig. 2b**, aged under ISOS-L-2 protocol (0.76-sun, full-spectrum simulated sunlight, 75 ± 5 °C, ambient air with relative humidity in the laboratory = 50~60%). The XRD data are plotted on a log scale to show the impurities; PbI_2 (#), Br-rich light-induced segregated phases (+).[81] The peaks due to ITO are marked with *.

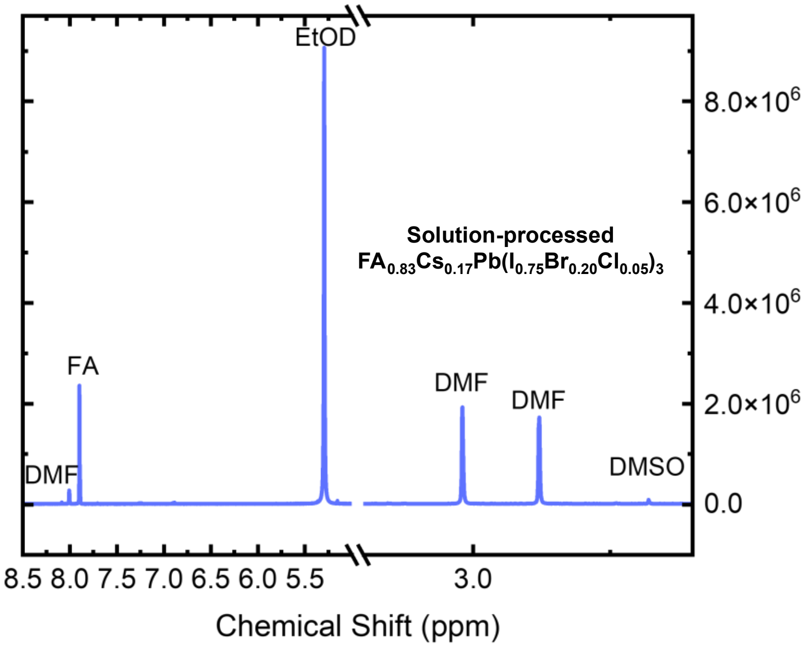

Supplementary Fig. 10. ^{1}H liquid NMR spectra of solution-processed perovskite materials. The perovskite materials were fabricated by spin coating with 4:1 v% DMF:DMSO and solvent-quenched by anisole. Spectra are obtained by dissolving material from ca. 90 cm^2 of perovskite thin film material (ca. 600 nm thickness) in deuterated ethanol (EtOD). Spectra are referenced to EtOD signal.

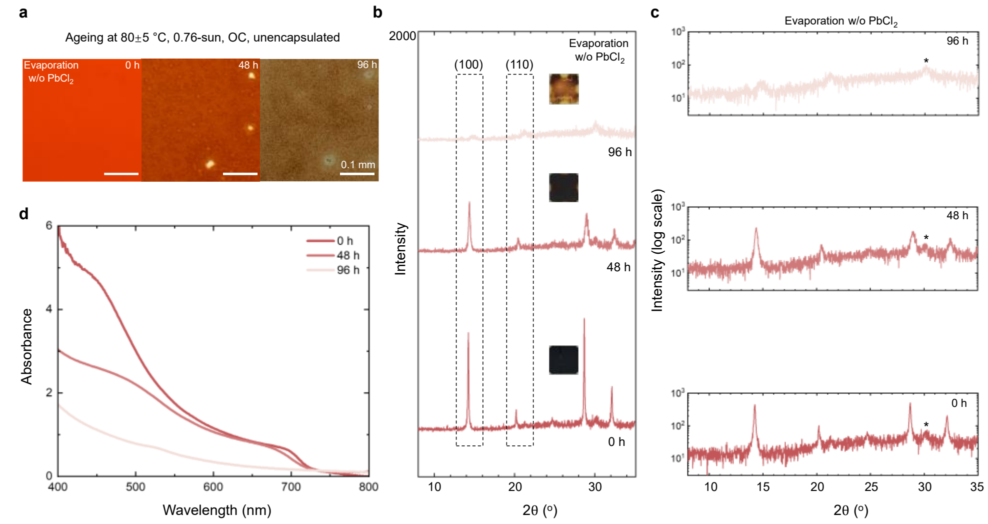

Supplementary Fig. 11. a, Optical microscopic images of an unencapsulated evaporated perovskite film using an absorber composition of $FA_{0.83}Cs_{0.17}Pb(I_{0.80}Br_{0.20})_3$, aged under ISOS-L-2 protocol (full-spectrum simulated sunlight, 75 ± 5 °C, ambient air with RH in the laboratory 50~60%). A thin layer of PMMA was deposited on the films to protect them from dust and scratches. Photos shown here were taken with an optical microscope with a 20x magnification rate. The scale bar is 0.1 mm for all the images. **b, c, d,** Evolution of the XRD patterns in absolute scale (b) and log scale (c) and the absorbance spectra (d) of the corresponding perovskite films shown in (a). Inset: photos of the perovskite films taken during ageing. The XRD patterns (b) in the log scale are shown in (c) to reveal any impurity phases formed during ageing.

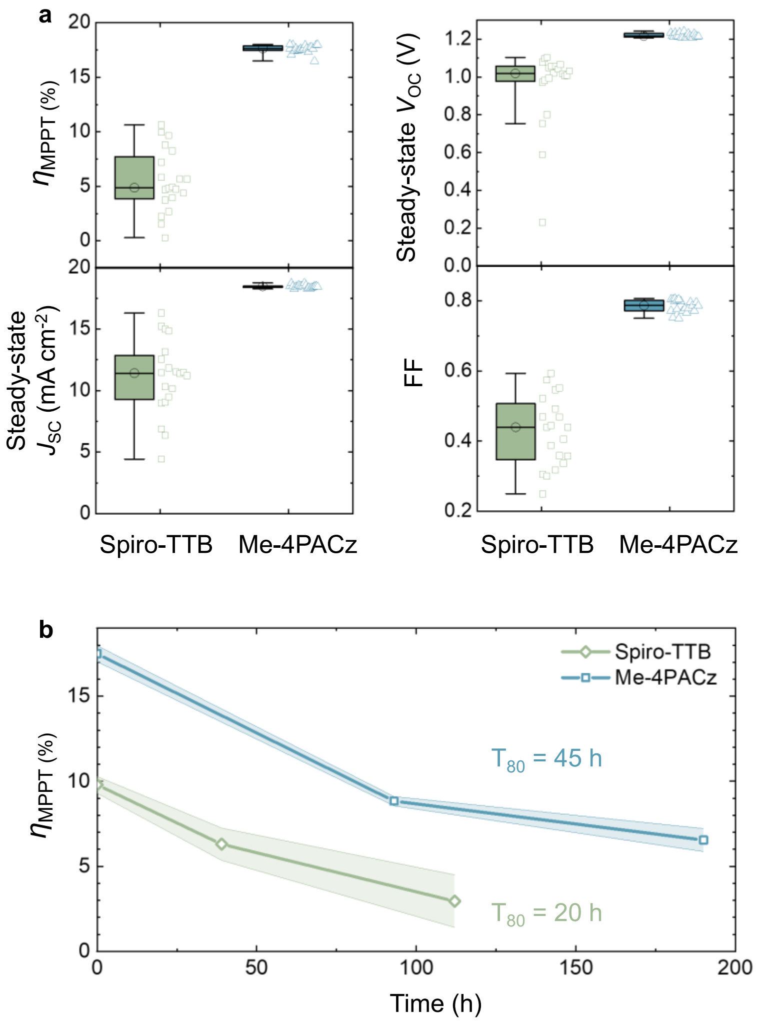

Supplementary Fig. 12. a, PV performance of solution-processed WBG PSCs, with solution-processed Spiro-TTB or Me-4PACz as the HTLs (20 cells for Spiro-TTB and 20 cells for Me-4PACz), and C_{60} and ALD SnO_2 as the ETLs. The absorber layer is of perovskite composition $FA_{0.83}Cs_{0.17}Pb(I_{0.75}Br_{0.20}Cl_{0.05})_3$. The middle line in a box plot shows the median, indicating the central tendency of the data. The box length reflects data spread, while the 'whiskers' mark the maximum and minimum values of the normal range. **b,** Evolution of the maximum power point tracking (MPPT) efficiencies (η) of encapsulated solution-processed WBG PSCs, with solution-processed Spiro-TTB or Me-4PACz as the HTLs, and C_{60} and ALD SnO_2 as the ETLs, aged under the ISOS-L-2 ageing protocol: 75 ± 5 °C, OC condition, 0.76-sun, full-spectrum simulated sunlight, relative humidity in the laboratory at 50~60%. The error bands represent the standard deviation of independent cells (n = 4 and 4 for Spiro-TTB and Me-4PACz, respectively), and the centres represent the median values. The T_{80} of the cells on spiro-TTB and Me-4PACz are 20 h and 45 h, respectively. Cr/Au electrodes were used in cells for stability test instead of Ag. In our preliminary test, solution-processed perovskites deposited on Me-4PACz shows better efficiency and stability than on solution-processed spiro-TTB. Therefore, we select Me-4PACz as the HTL for solution-processed PSCs.

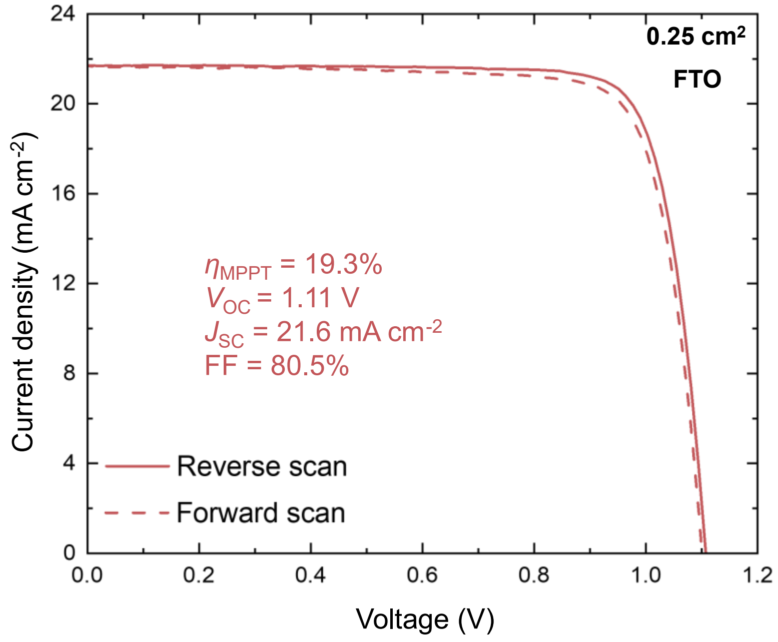

Supplementary Fig. 13. *J–V* characteristics of the representative evaporated perovskite solar cell of $FA_{0.83}Cs_{0.17}Pb(I_{0.75}Br_{0.20}Cl_{0.05})_3$ with ALD SnO_2 buffer layer, deposited on FTO substrate with anti-reflective foil applied to minimise the optical loss. The aperture size is 0.25 cm^2.

福建省计量科学研究院
FUJIAN METROLOGY INSTITUTE
（国家光伏产业计量测试中心）
National PV Industry Measurement and Testing Center

24Q3-00622

检测结果/说明：
Results of Test and additional explanation.

1. Standard Test Condition (STC)：Total Irradiance：1000 W/m^2
Temperature：25.0 °C
Spectral Distribution：AM1.5G

2.Measurement Data and I-V/P-V Curves under STC

Forward Scan

I_{sc} (mA)	V_{oc}(V)	I_{MPP}(mA)	V_{MPP}(V)	P_{MPP} (mW)	FF(%)	A (cm^2)
5.181	1.111	4.835	0.9418	4.554	79.12	0.2500

Reverse Scan

I_{sc} (mA)	V_{oc}(V)	I_{MPP}(mA)	V_{MPP}(V)	P_{MPP} (mW)	FF(%)	A (cm^2)
5.194	1.113	4.880	0.9465	4.619	79.90	0.2500

Mismatch factor:0.9940

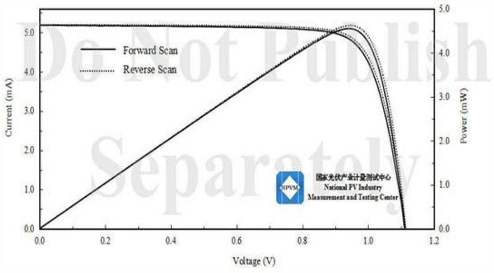

Figure 1. I-V and P-V characteristic curves of the measured sample under STC

检测报告续页专用
Continued page of test report

第 3 页/共 6 页
Page of Pages

Supplementary Fig. 14. The certificate of our representative all vacuum-deposited WBG perovskite solar cell (E_g^{PV} = 1.67 eV) of $FA_{0.83}Cs_{0.17}Pb(I_{0.75}Br_{0.20}Cl_{0.05})_3$ with SnO_2 buffer layer (*J*–*V* characterization). The report is issued by NPVM (Chinese national PV industry measurement and testing center). The aperture area is 0.25 cm^2 measured by NPVM. The report includes the optical aperture area, *J*–*V*, MPPT, and EQE measurements.

检测结果/说明：
Results of Test and additional explanation.

3.Measurement Data and Curves for MPPT under STC

η (%)	18.35
P_{MPP} (mW)	4.587
I_{MPP} (mA)	4.922
V_{MPP} (V)	0.9320

Note: Measurement data for MPPT under STC in the above table was the mean value acquired during the final 30 seconds of the 300 seconds test

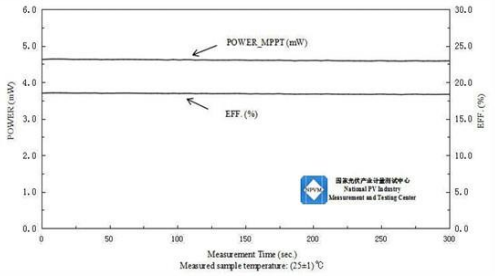

Figure 2. Measurement curves of the measured sample for MPPT

/

Supplementary Fig. 15. The certificate of our representative all vacuum-deposited perovskite solar cell (E_g^{PV} = 1.67 eV) of $FA_{0.83}Cs_{0.17}Pb(I_{0.75}Br_{0.20}Cl_{0.05})_3$ with SnO_2 buffer layer (stabilized PCE characterization). The report is issued by NPVM (Chinese national PV industry measurement and testing center). The maximum power point tracked PCE of 18.35% is determined from MPPT for 300 s and averaged between 3 continuous, repeated measurements.

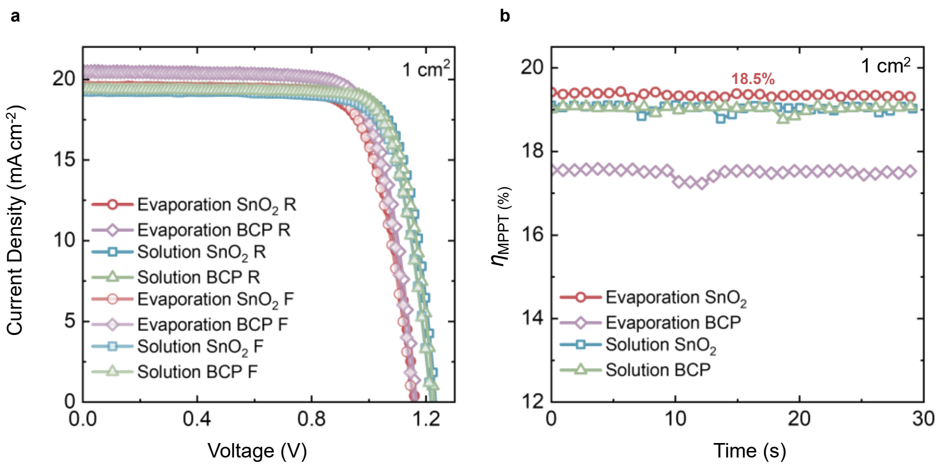

Supplementary Fig. 16. a–b, *J–V* characteristics (a) and 30 s of MPPT tracking (b) of the representative evaporated or solution-processed perovskite solar cells of $FA_{0.83}Cs_{0.17}Pb(I_{0.75}Br_{0.20}Cl_{0.05})_3$ with an aperture size of 1 cm^2, with either ALD SnO_2 or evaporated BCP buffer layer. R: reverse scan, F: forward scan. The cells shown in this figure have anti-reflective foils applied on the glass side to reduce the optical loss. Detailed PV metrics can be found in **Supplementary Table. 1**.

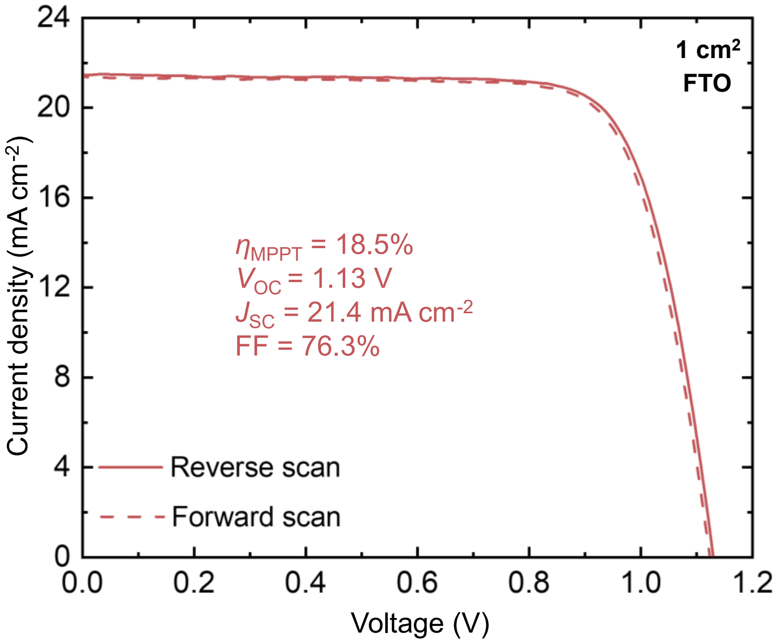

Supplementary Fig. 17. *J–V* characteristics of the representative evaporated perovskite solar cell of $FA_{0.83}Cs_{0.17}Pb(I_{0.75}Br_{0.20}Cl_{0.05})_3$ with ALD SnO_2 buffer layer, deposited on FTO substrate with anti-reflective foil applied to minimise the optical loss. The aperture size is 1 cm^2.

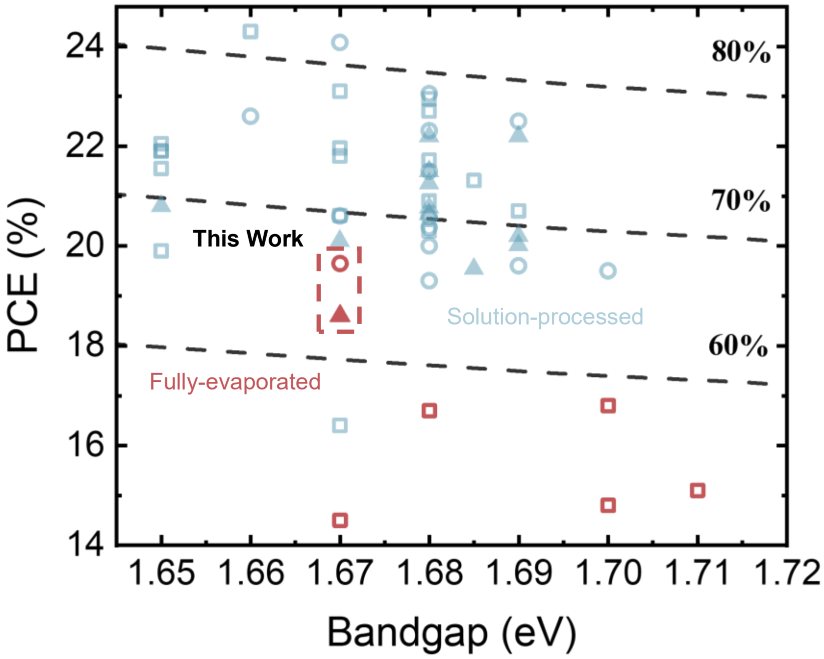

Supplementary Fig. 18. Representative PCE versus bandgap data of high-efficiency p-i-n WBG PSCs from the literature.[1,4–16,18,20–22,24,26–29,36,38,63,82–97] All data are extracted from reverse scans. Only devices with a bandgap compatible for perovskite-on-silicon tandem applications (E_g = 1.65–1.72 eV) are included in the chart due to the relevance to this work. Black dash lines: the detailed balance limit of single-junction solar cells. Literature data are represented with different symbols according to their active pixel area and device stacks: square: <0.1 cm^2; circle: 0.1–0.3 cm^2; triangle: close to 1 cm^2. Red symbols: all vacuum-deposited cells; blue symbols: cells with solution-processed absorber layers or transport layers or both. Our 0.25 cm^2 and 1 cm^2 champion all vacuum-deposited cells of 1.67 eV ($FA_{0.83}Cs_{0.17}Pb(I_{0.75}Br_{0.20}Cl_{0.05})_3$) are marked as red circle and triangle, respectively.

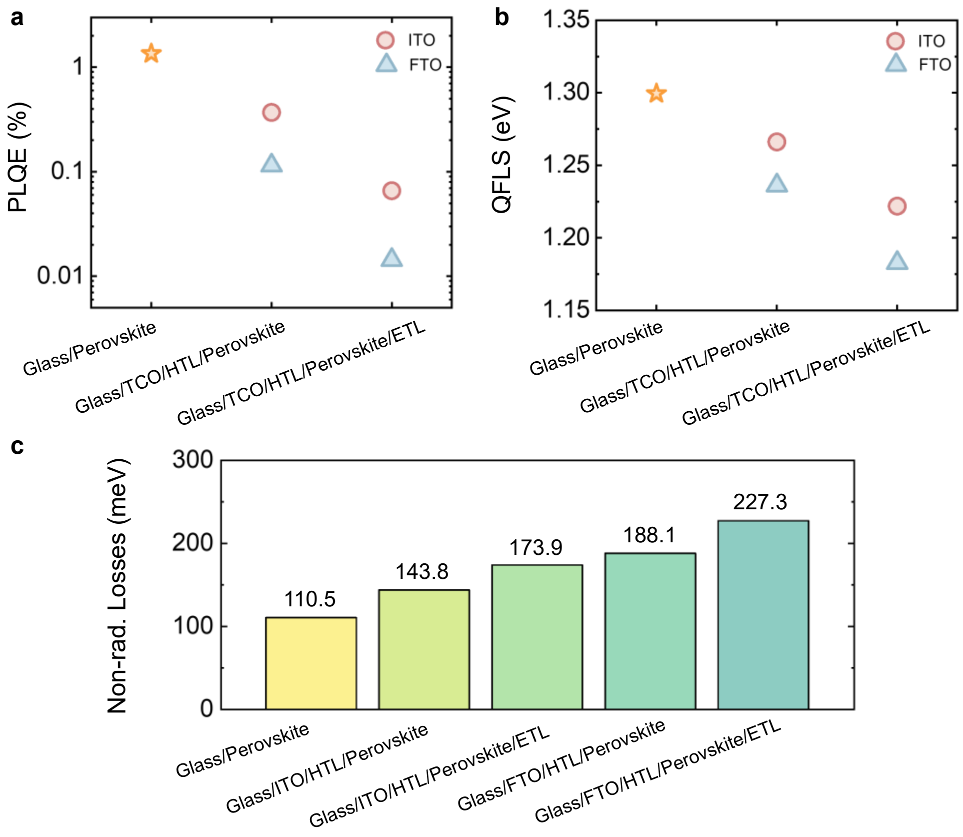

Supplementary Fig. 19. a–b, Photoluminescence quantum efficiency (PLQE) (a) and quasi-Fermi level splitting (QFLS) for neat co-evaporated perovskite thin-films on glass substrates, and various device "half-stacks": glass/TCO (ITO or FTO)/HTL/perovskite; and glass/TCO (ITO or FTO)/HTL/perovskite/ETL. **c**, Non-radiative energy losses with respect to the theoretical radiative limit of the corresponding samples in (b). The HTL and ETL in all stacks are evaporated Spiro-TTB and C_{60}.

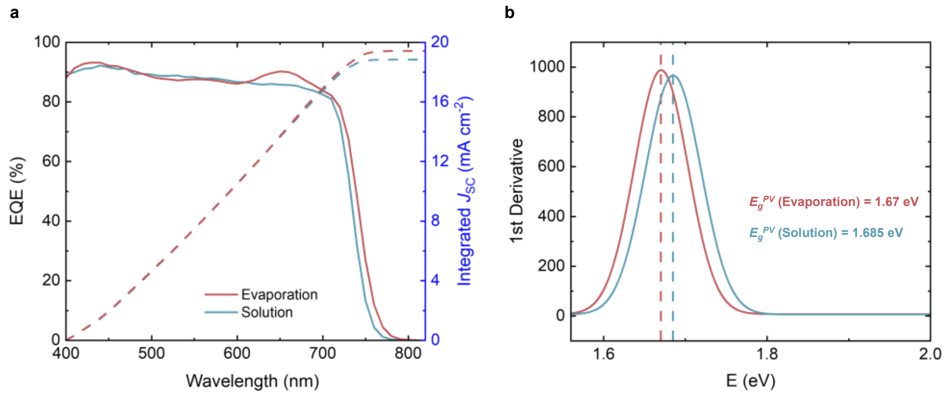

Supplementary Fig. 20. a, EQE spectra (left axis) and the corresponding integrated J_{SC} (integration of the product of EQE and AM 1.5G spectrum) (right axis) of the representative evaporated or solution-processed perovskite solar cells of $FA_{0.83}Cs_{0.17}Pb(I_{0.75}Br_{0.20}Cl_{0.05})_3$ with an aperture size of 0.25 cm^2, with ALD SnO_2 buffer layer. **b,** PV bandgap (E_g^{PV}) extracted from the inflection point of the onset of the EQE shown in (a). The slightly wider bandgap of the solution-processed PSC is likely due to a higher Cl^- ratio remained in the absorber layer as evidenced by the time-of-flight secondary ion mass spectrometry (ToF-SIMS, **Supplementary Fig. 21**).

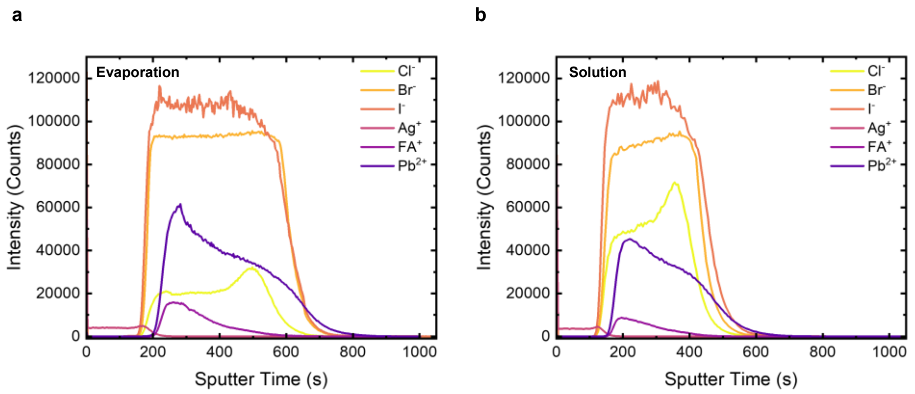

Supplementary Fig. 21. a–b, ToF-SIMS depth profiles of the evaporated (a) and solution-processed (b) perovskite solar cells of $FA_{0.83}Cs_{0.17}Pb(I_{0.75}Br_{0.20}Cl_{0.05})_3$, measured in positive and negative polarity. The device architecture of the evaporated cell is ITO/Spiro-TTB/$FA_{0.83}Cs_{0.17}Pb(I_{0.75}Br_{0.20}Cl_{0.05})_3$ /C_{60}/SnO_2/Ag. The device architecture of solution-processed cell is ITO/Me-4PACz/ $FA_{0.83}Cs_{0.17}Pb(I_{0.75}Br_{0.20}Cl_{0.05})_3$ /C_{60}/SnO_2/Ag. Except Me-4PACz which was solution-processed, all the other layers were fabricated by thermal evaporation.

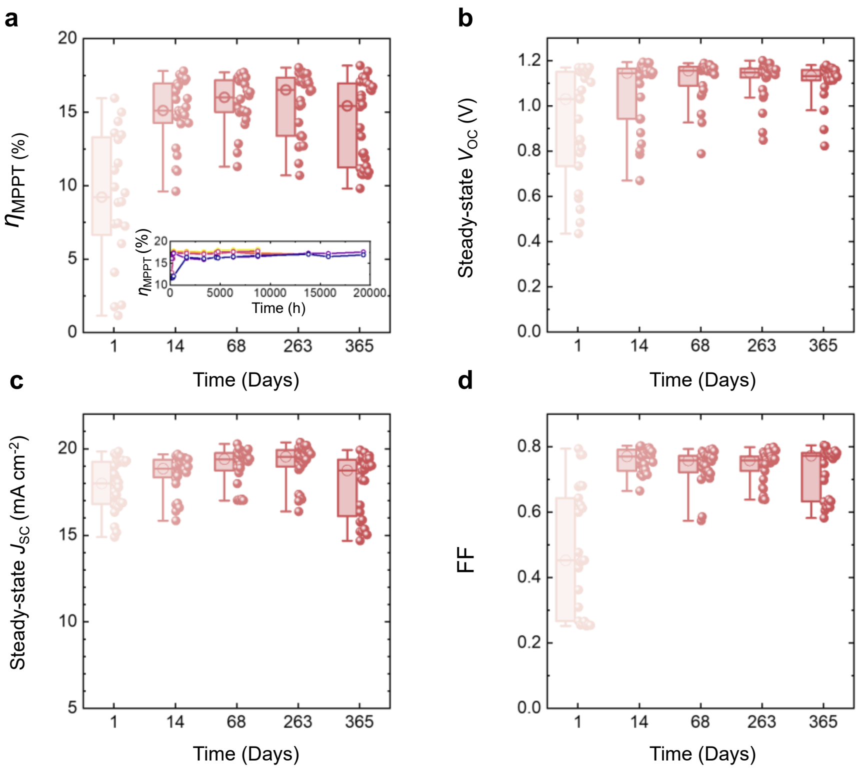

Supplementary Fig. 22. a–d, Shelf lifetime of one batch (n = 28 cells) of crystal-facet-directed, seed-free co-evaporated perovskite solar cells of $FA_{0.83}Cs_{0.17}Pb(I_{0.75}Br_{0.20}Cl_{0.05})_3$, with ALD SnO_2 buffer layer. The unencapsulated cells were stored under ISOS-D-1 protocol (ambient temperature, dark) and measured periodically in ambient condition (relative humidity = 50–60%). The PV parameters include η_{MPPT} (a), steady-state V_{OC} (b), steady-state J_{SC} (c), and FF (d). Inset: Shelf lifetime of several representative cells with T_{100} ~ 20,000 hours. The middle line in a box plot shows the median of 28 cells, indicating the central tendency of the data. The box length reflects data spread, while the 'whiskers' mark the maximum and minimum values of the normal range. These cells demonstrate long shelf lifetimes with nearly no loss in efficiency after more than 1 year, with several cells maintaining their peak performance ($\eta_{MPPT} \approx 18\%$) after nearly 20,000 hours (> 2 years) of storage in a nitrogen glovebox. We note that this "stability" test is very mild and gives no real indication of expected lifetime during operation in the field. However, since the isolated un-annealed perovskite films degrade within hours, this indicates that appropriate crystallisation can entirely overcome these short-term instabilities. A long "shelf-life" is also useful if cells or modules need to be stored for some time after manufacturing before lamination and/or encapsulation.

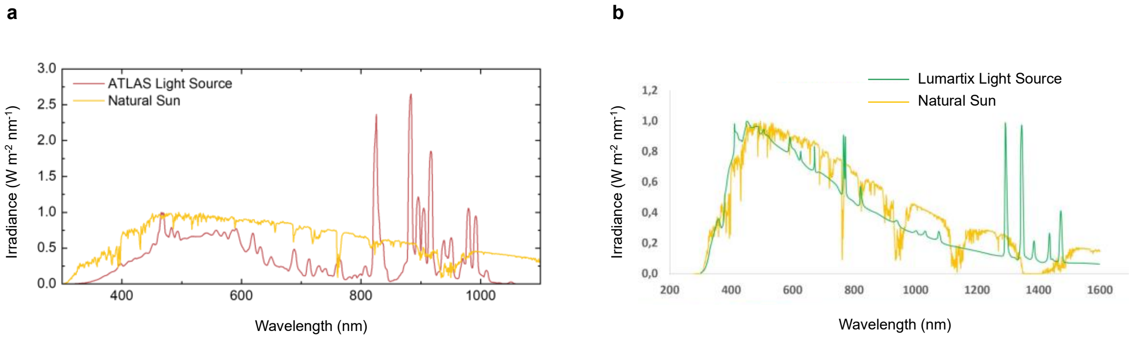

Supplementary Fig. 23. a–b, The full spectra of simulated sunlight used for ISOS-L-2 ageing study carried out at University of Oxford (a), and Hong Kong University of Science and Technology (HKUST) (b), respectively. The corresponding stability data are presented in **Fig. 3**, and **Figs. 4–5**, respectively. A comparison between the simulated solar spectra and the natural sun spectrum (AM1.5G) are shown in the figure. No UV filter was used in all the ageing tests.

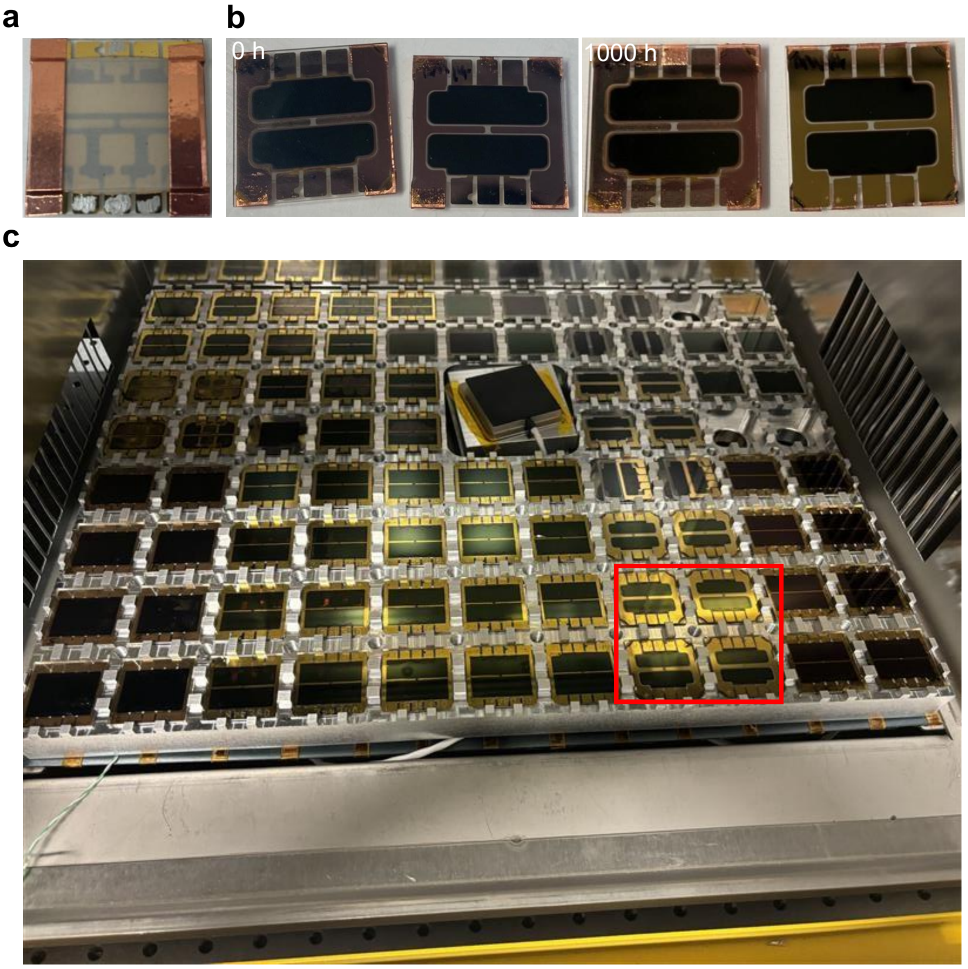

Supplementary Fig. 24. a, Photos of the front side of the evaporated cells after encapsulation with epoxy sealant. Cr/Au electrodes were used instead of Ag for stability test. **b,** Photos of the back side of the evaporated cells before and after 1000 hours ageing under ISOS-L-2 protocol. Copper tape and silver paste was applied to help maintain electrical contact over the ageing period. **c,** Photos of the encapsulated cells in the ATLAS ageing box with simulated sunlight (0.76 sun) and heating stage (75 ± 5 °C). Cells marked in red square are the crystal-facet-directed co-evaporated cells.

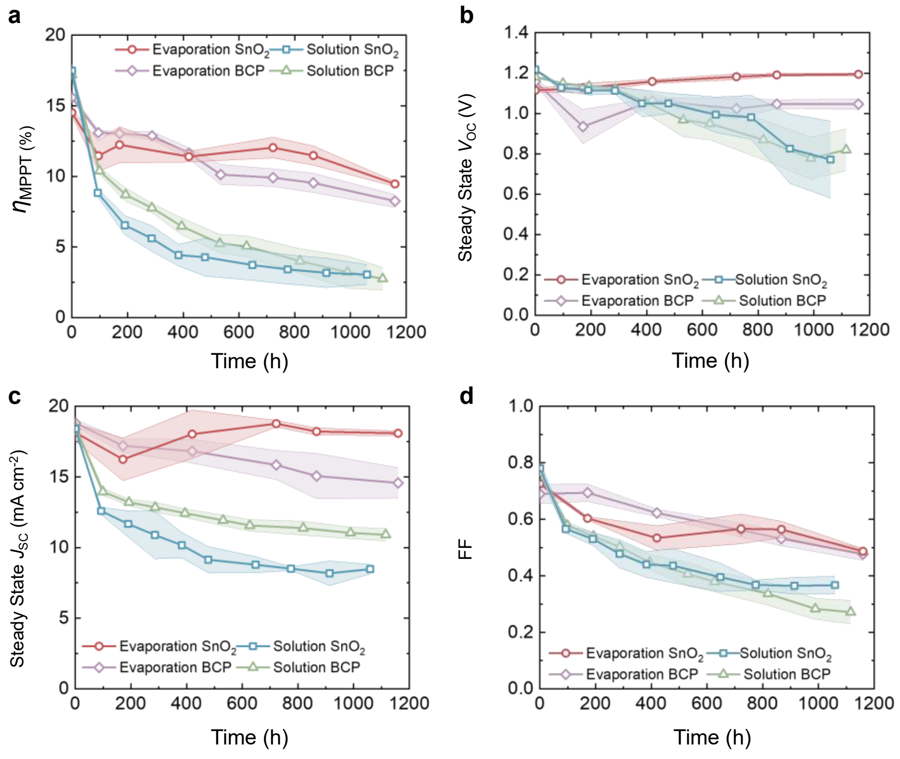

Supplementary Fig. 25. a–d, Evolution of η_{MPPT} (a), steady-state V_{OC} (b), steady-state J_{SC} (c), and FF (d), of encapsulated evaporated or solution-processed perovskite solar cells with an aperture size of 0.25 cm^2 or 1 cm^2, with either ALD SnO_2 or evaporated BCP buffer layers aged under ISOS-L-2 protocol: OC condition, full-spectrum simulated sunlight, at 75 ± 5 °C, in ambient air with relative humidity in the laboratory at 50~60%. The error bands represent the standard deviation of independent cells ($n = 5$, 6, 8, and 8 for evaporation SnO_2, evaporation BCP, solution SnO_2 and solution BCP, respectively), and the centres represent the median values.

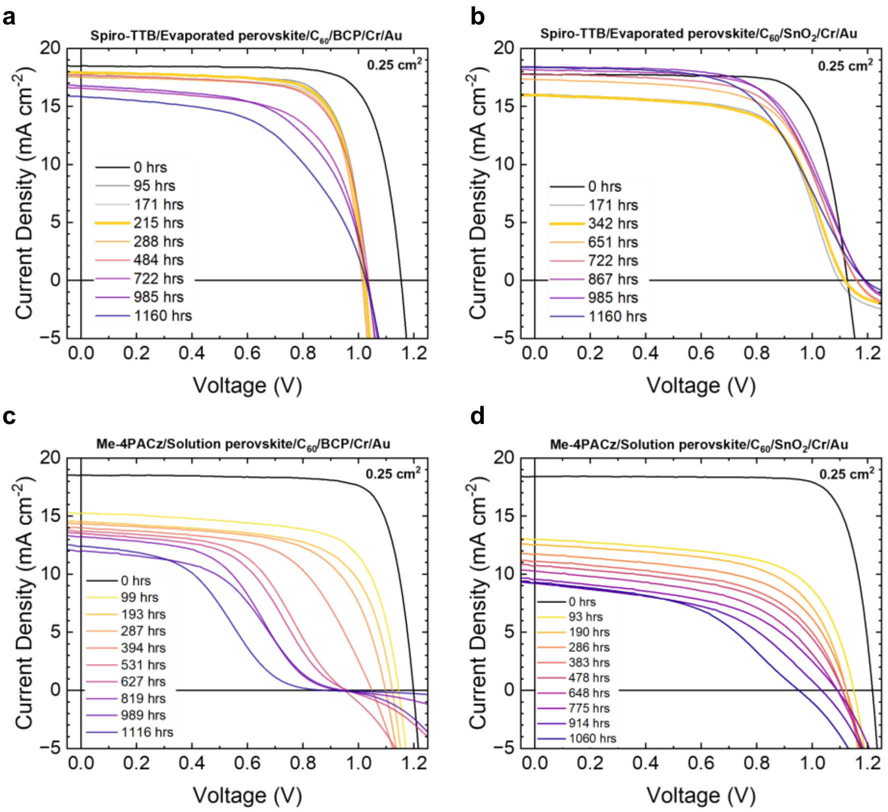

Supplementary Fig. 26. Evolution of the light *J*–*V* curves (reverse scan) for the evaporated and solution-processed champion cells with an aperture size of 0.25 cm^2 as shown in **Fig. 3d**.

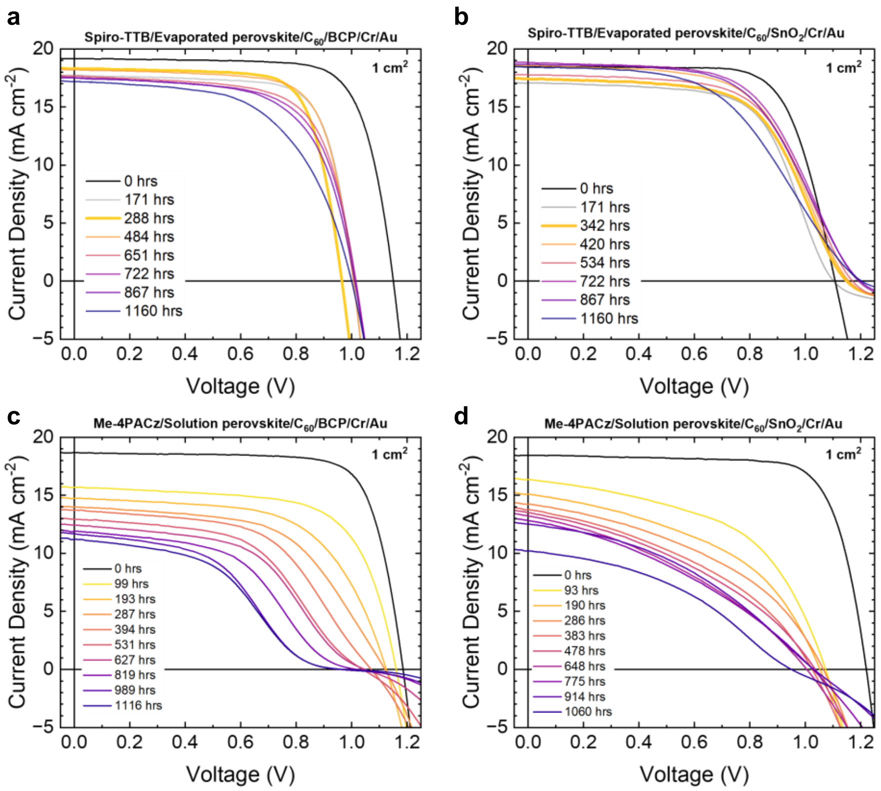

Supplementary Fig. 27. Evolution of the light *J*–*V* curves (reverse scan) for the evaporated and solution-processed champion cells with an aperture size of 1 cm^2 as shown in **Fig. 3d**.

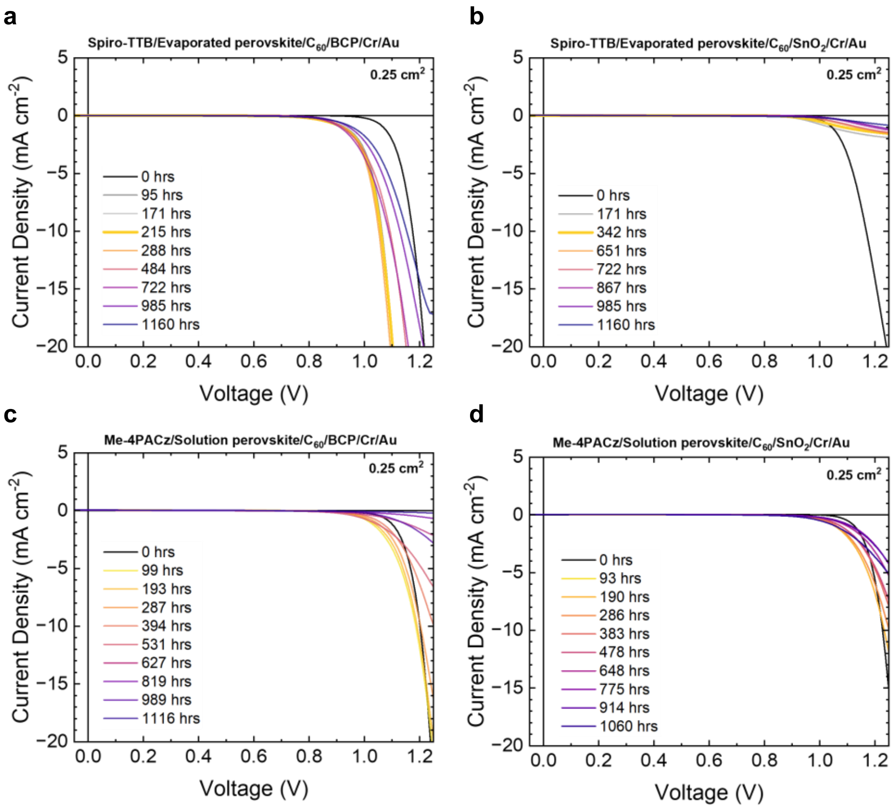

Supplementary Fig. 28. Evolution of the dark *J*–*V* curves (reverse scan) for the evaporated and solution-processed champion cells with an aperture size of 0.25 cm^2 as shown in **Fig. 3d**.

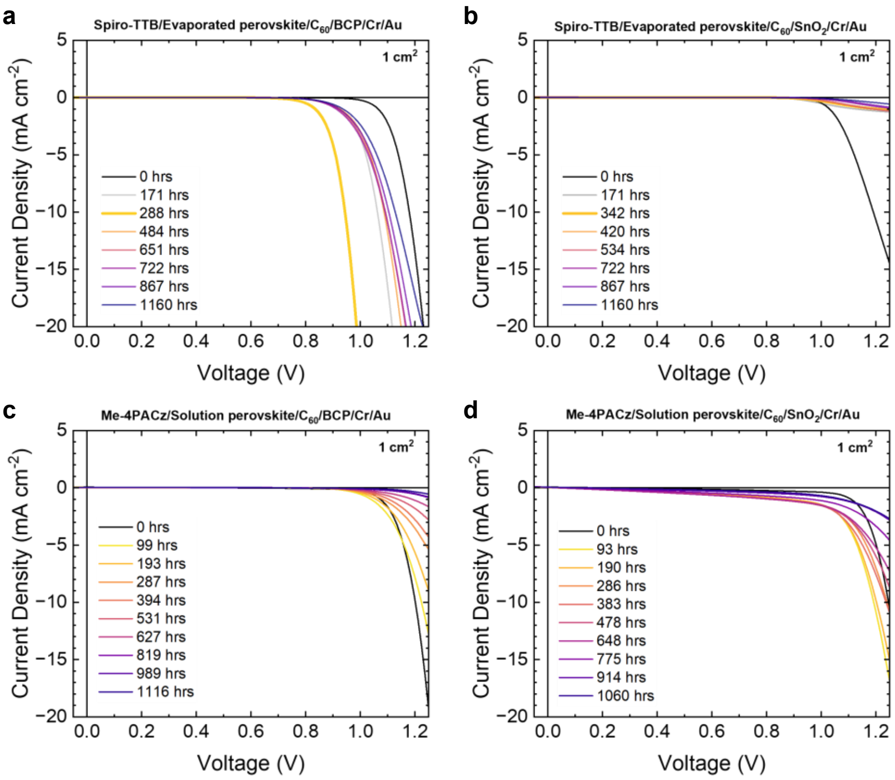

Supplementary Fig. 29. Evolution of the dark *J*–*V* curves (reverse scan) for the evaporated and solution-processed champion cells with an aperture size of 1 cm^2 as shown in **Fig. 3d**.

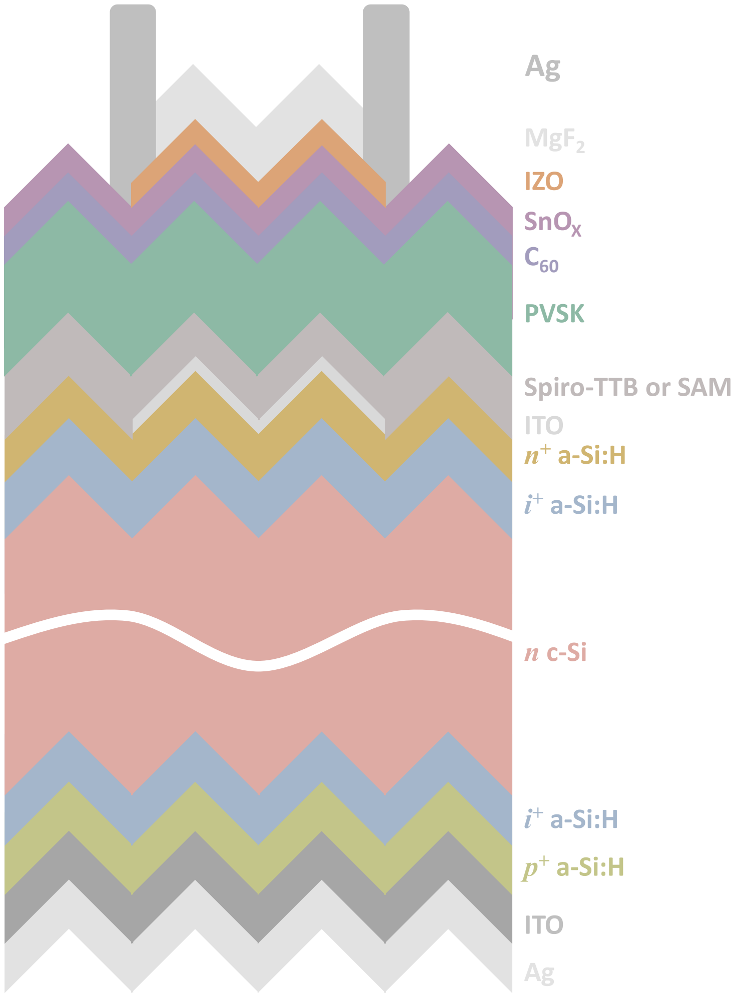

Supplementary Fig. 30. Device architecture of our evaporated perovskite-on-silicon tandem solar cell based on industrial standard Czochralski (CZ), 150-micron thick n-type silicon heterojunction cells with micron-scale textured structure.

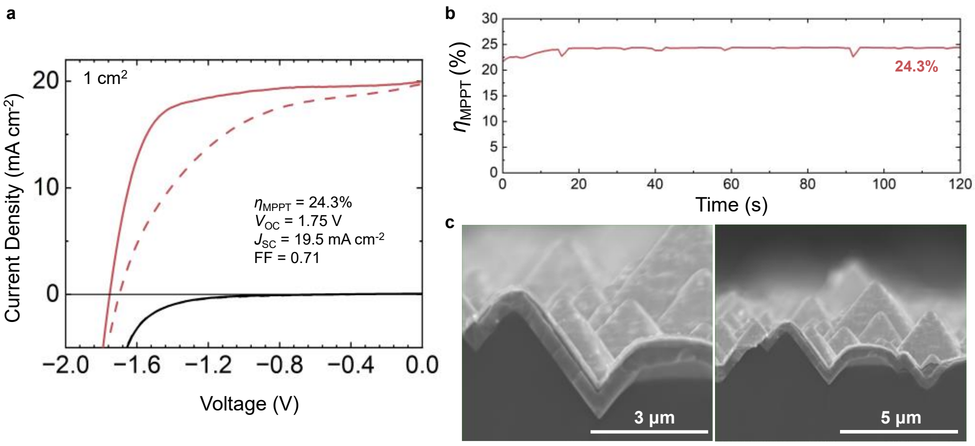

Supplementary Fig. 31. a–b, *J–V* characteristics (a) and η_{MPPT} (b) of a 1 cm^2 representative all vacuum-deposited perovskite-on-silicon tandem solar cell fabricated via crystal-facet-directed co-evaporation of $FA_{0.83}Cs_{0.17}Pb(I_{0.75}Br_{0.20}Cl_{0.05})_3$ based on a micron-textured industrial-standard HJT Si wafer. Hysteresis was likely induced by contaminated ALD TDMASn source due to a source leakage happened before the time of device fabrication. **c,** Cross-sectional SEM of the perovskite-on-silicon tandem in (a).

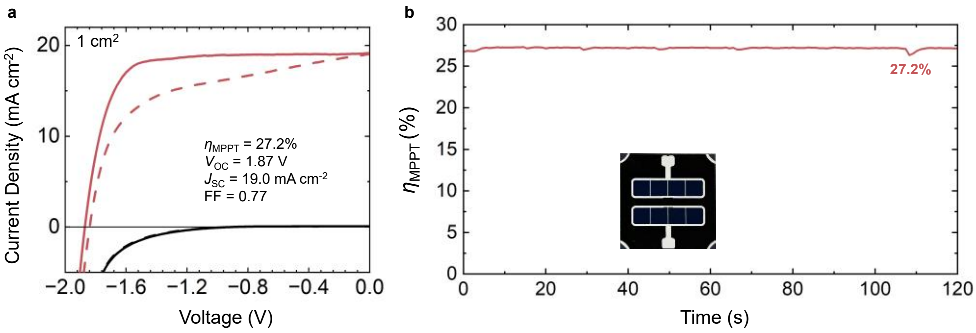

Supplementary Fig. 32. a–b, *J–V* characteristics (a) and η_{MPPT} (b) of a 1 cm^2 representative evaporated perovskite-on-silicon tandem solar cell fabricated with solution-processed SAM HTL (**Methods**) via crystal-facet-directed co-evaporation of $FA_{0.83}Cs_{0.17}Pb(I_{0.75}Br_{0.20}Cl_{0.05})_3$ based on a micron-textured industrial-standard HJT Si wafer. Hysteresis was likely induced by contaminated ALD TDMASn source due to a source leakage happened before the time of device fabrication. Inset: photo of the tandem cell with two 1-cm^2 pixels.

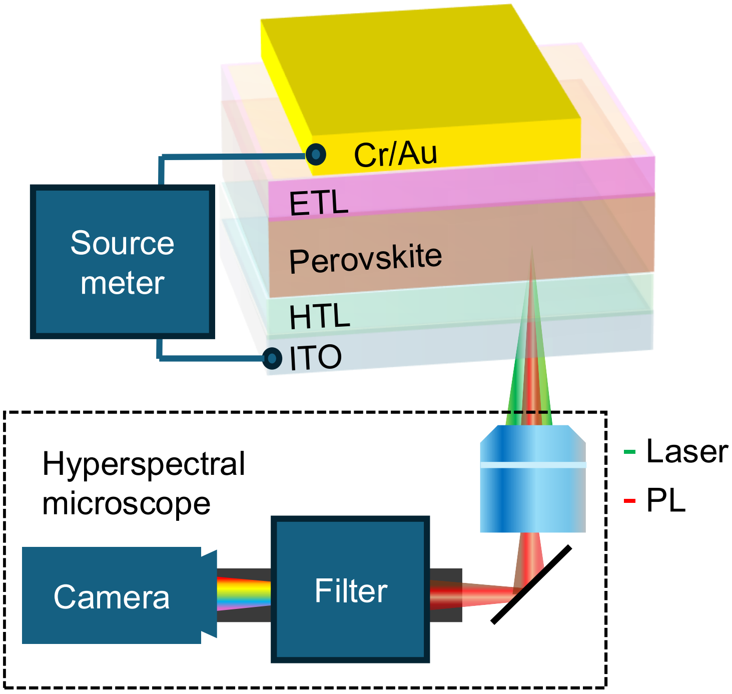

Supplementary Fig. 33. Schematic of our hyperspectral microscope setup. The samples were illuminated using a 532-nm laser (intensity = 1.71 sun) as the excitation source from the substrate side.

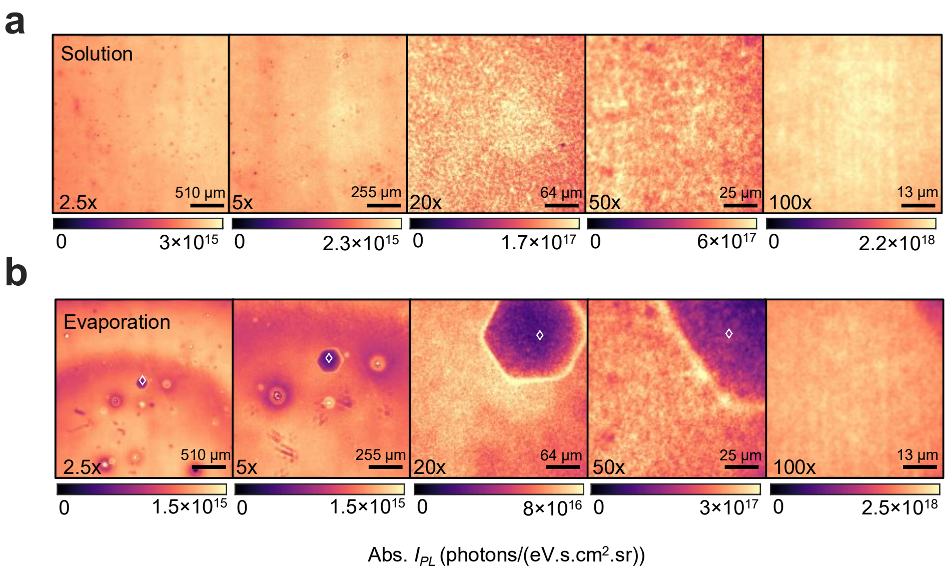

Supplementary Fig. 34. a–b, Absolute PL intensity (I_{PL}) taken for solution-processed (a) and co-evaporated (b) perovskite solar cells with different objective magnifications (2.5x (2656 µm x 2656 µm), 5x (1328 µm x 1328 µm), 20x (332 µm x 332 µm), 50x (133 µm x 133 µm), 100x (66.4 µm x 66.4 µm)) at the selected wavelength (i.e., 734 nm and 740 nm, respectively) corresponding to the λ_{mean} as identified in the spatial-spectral data, i.e., hyperspectral data cube. The dark hexagonal region marked with white triangles on the hyperspectral data cubes of evaporated perovskite was caused by beam damage during calibration.

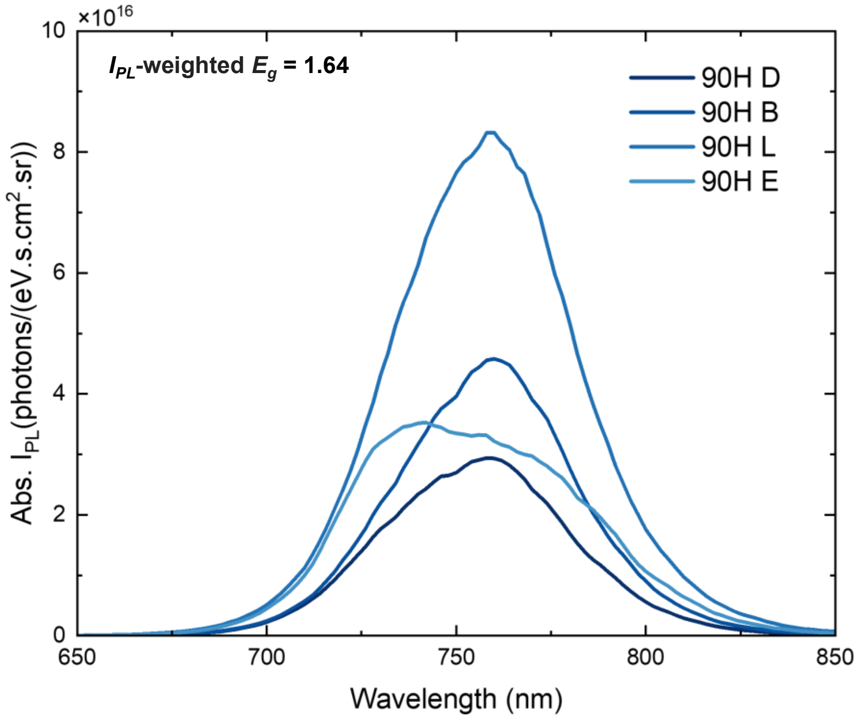

Supplementary Fig. 35. PL spectra with multiple emissive states and relative intensities at different wavelengths captured at various spots on a hyperspectral data cube for a solution-processed perovskite solar cell after 90 hours ageing under ISOS-L-2 protocol. All the spectra lead to the same I_{PL}-weighted E_g value of 1.64 eV. B: bright wrinkle area; D: dark wrinkle area; L: brighter spots on the bright wrinkles; E: hotspots with extra red-shifted PL spectra.

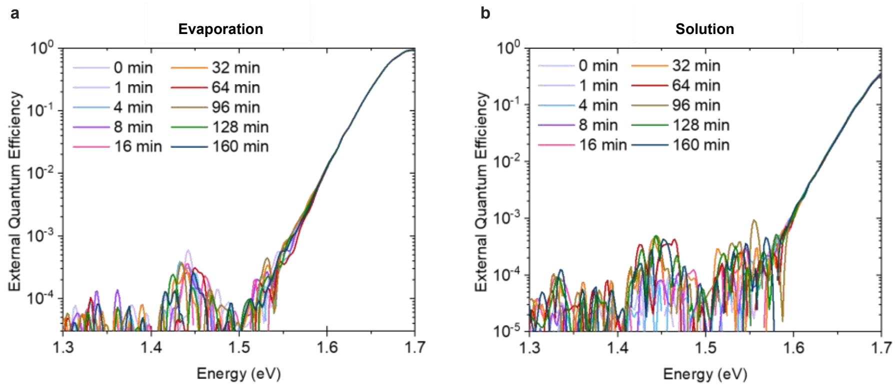

Supplementary Fig. 36. a–b, FTPS measurements. Semi-log plots of the EQE at the absorption onsets of unencapsulated evaporated (a) and solution-processed (b) perovskite solar cells with SnO_2 buffer layer and Cr/Au electrodes over 160 mins of continuous illumination under a xenon lamp in air (relative humidity in lab = 40–50%). Two measurements with different RG filters were performed. Data with energy less than 1.59 eV are filtered from measurement with the long-pass RG780 filter.

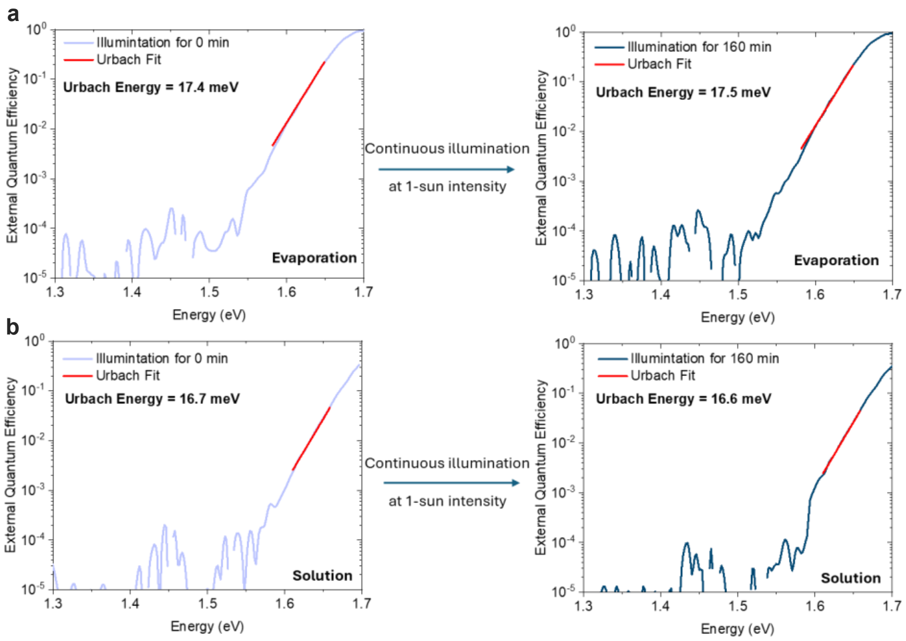

Supplementary Fig. 37. a–b, Fitted Urbach tails from a section of EQE below the band edge of the unencapsulated evaporated (a) and solution-processed (b) perovskite solar cells shown in **Supplementary Fig. 36** before and after 160 minutes of continuous illumination in air (relative humidity in lab = 40–50%).

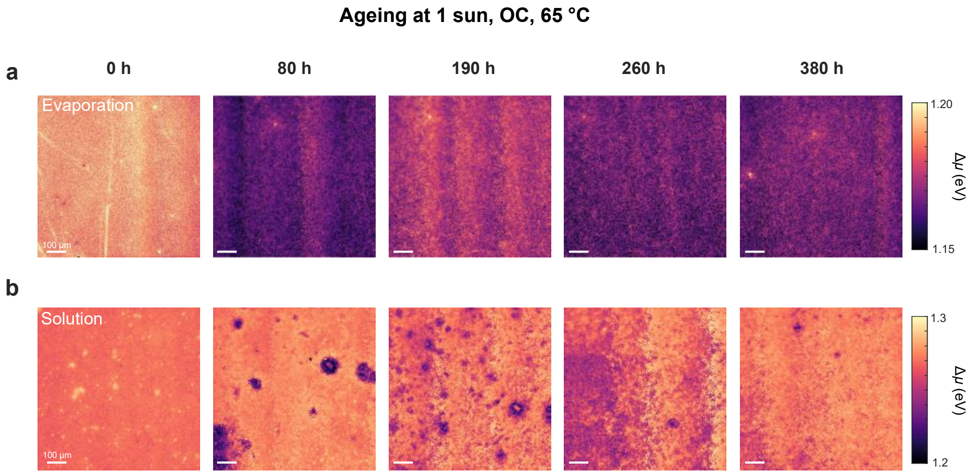

Supplementary Fig. 38. QFLS ($\Delta\mu$) mappings for the corresponding co-evaporated and solution-processed cells with ALD SnO_2 as shown in **Fig. 4** over 380 h of ageing under ISOS-L-2 conditions, calculated using the method described in **Supplementary Note 7**. Scale bar: 100 μm.

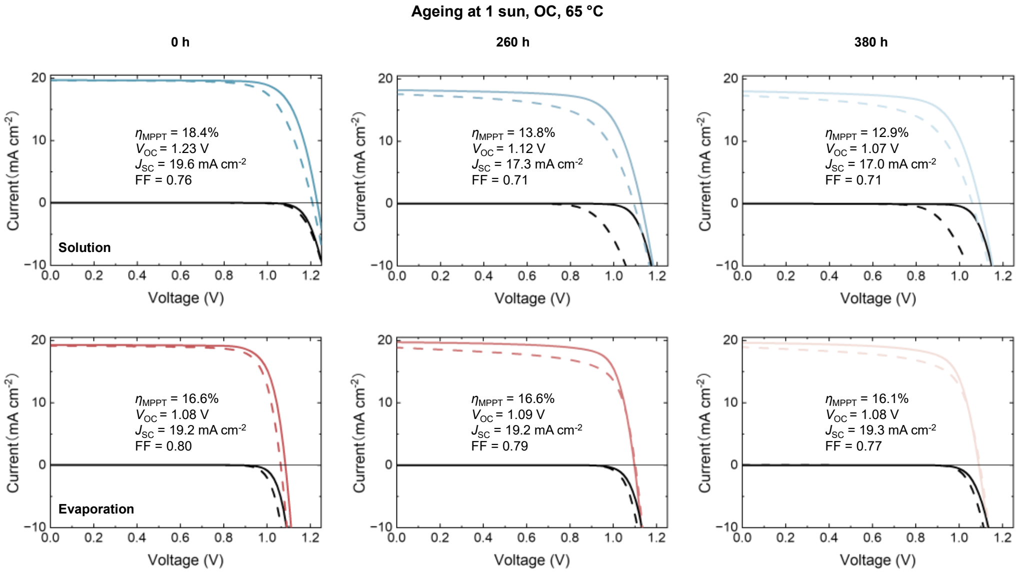

Supplementary Fig. 39. Evolution of the real *J*–*V* curves of the corresponding solution-processed and evaporated cells with ALD SnO_2 as shown in **Fig. 4** before and after 380 h of ageing under ISOS-L-2 conditions: OC, at 65 ± 5 °C, and 1 sun full-spectrum illumination, in ambient (relative humidity in lab = 80–90%).

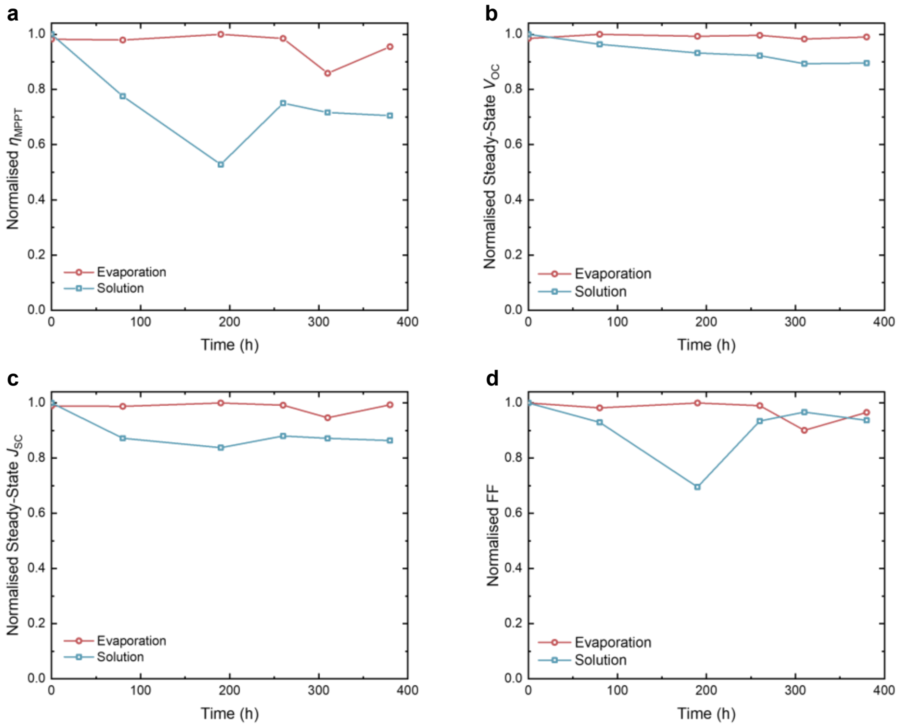

Supplementary Fig. 40. a–d, Evolution of the normalised η_{MPPT} (a), steady-state V_{OC} (b), steady-state J_{SC} (c) and FF (d) for co-evaporated and solution-processed perovskite solar cells with ALD SnO_2 as shown in **Fig. 4** over 380 hours of ageing under ISOS-L-2 protocol.

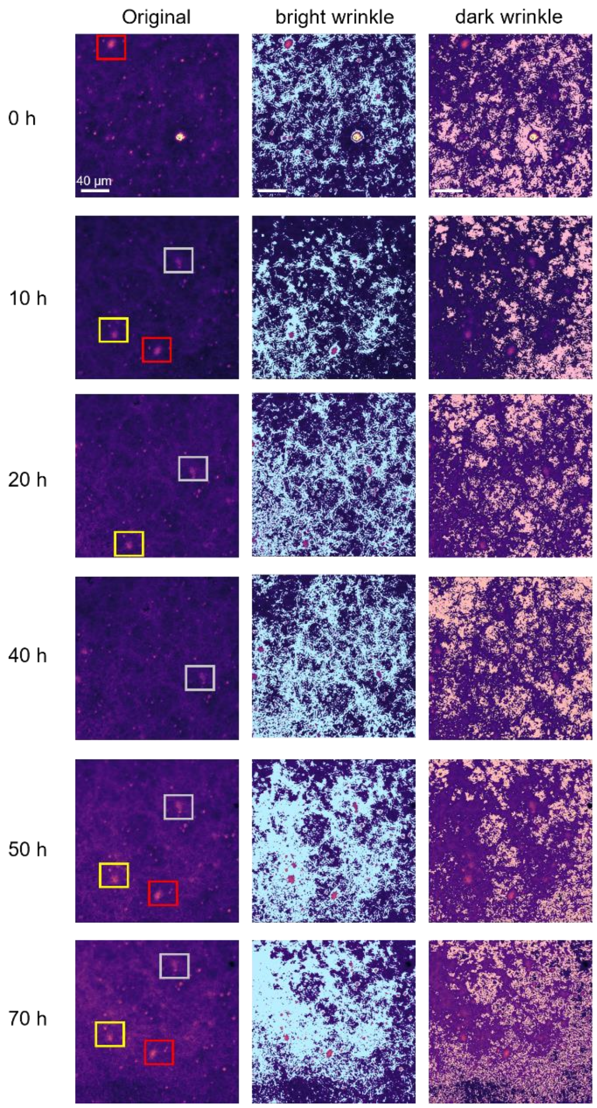

Supplementary Fig. 41. Hyperspectral data cubes for solution-processed perovskite solar cells to demonstrate the evolution of wrinkles over the initial 70 hours of ageing. The mappings of bright wrinkle area (as marked with squares) and dark winkle area are presented separately. Scale bar: 40 µm.

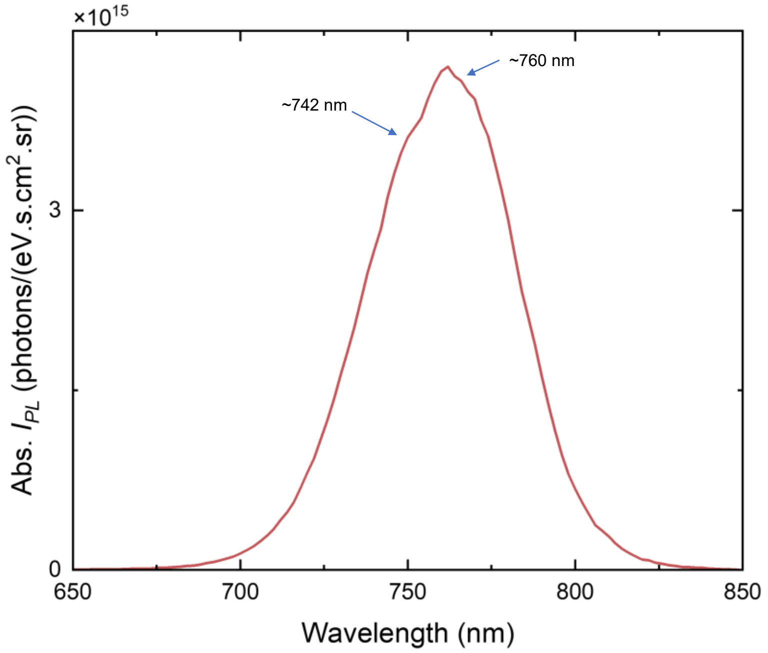

Supplementary Fig. 42. PL spectrum captured on the hyperspectral data cube for a co-evaporated cell with ALD SnO_2 over 380 hours of ageing under ISOS-L-2 protocol. The concurrent presence of a sub peak at ~742 nm and a main peak at ~760 nm show that the segregated I-rich domains on the mapping area are still radiatively connected with the mixed-halide phase.

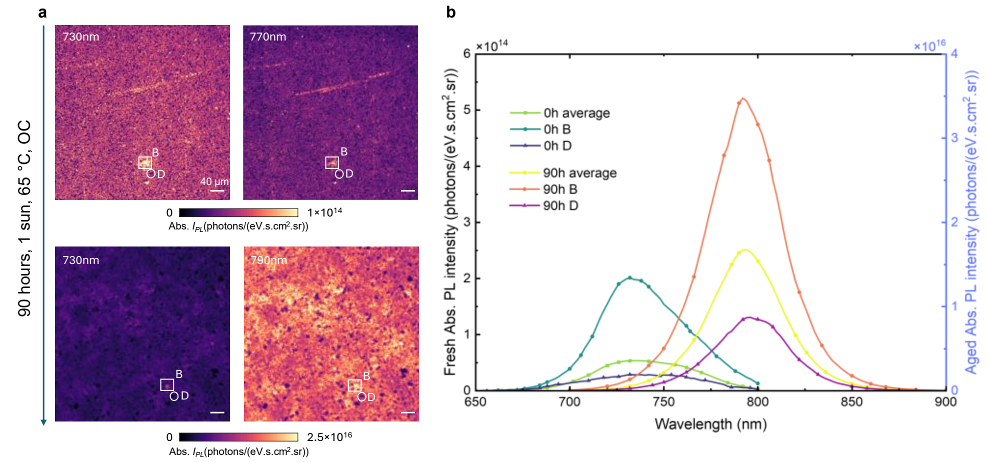

Supplementary Fig. 43. a, The hyperspectral data cubes at different selected wavelengths for co-evaporated PSCs without $PbCl_2$ before and after 90 hours of ageing under ISOS-L-2 protocol (OC, 65 ± 5 °C, and 1 sun full-spectrum illumination). Regions with different PL features on the mappings are marked with various symbols, and the corresponding PL spectra are shown separately. Square: bright spot (B); circle: dark spot (D). Scale bar: 40 µm. **b,** PL spectra of the corresponding marked regions in (a).

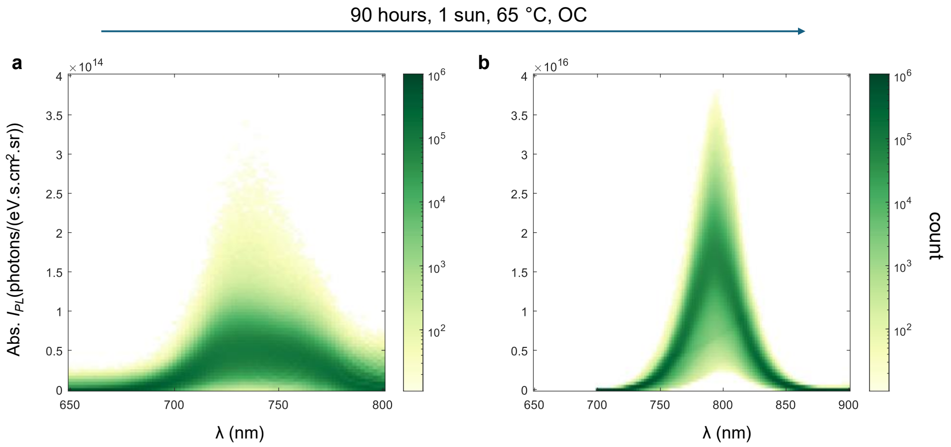

Supplementary Fig. 44. a-b, Density plots as a function of λ and absolute I_{PL} for co-evaporated cells with no $PbCl_2$ before (a) and after (b) 90 hours of ageing under ISOS-L-2 protocol as shown in **Supplementary Fig. 43**.

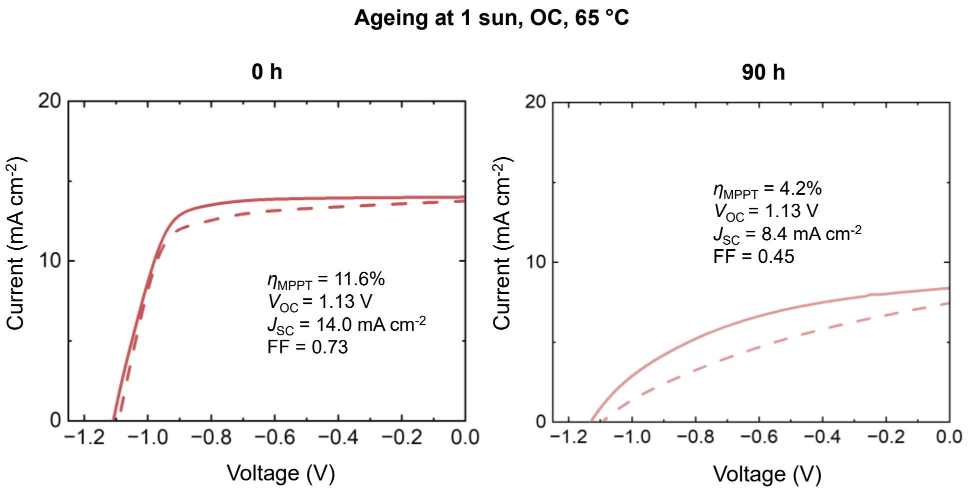

Supplementary Fig. 45. Evolution of the device *J*-*V* curves of the corresponding evaporated perovskite solar cells without $PbCl_2$ as shown in **Supplementary Fig. 43** during 90 h of ageing at OC, 65 °C, and 1 sun illumination.

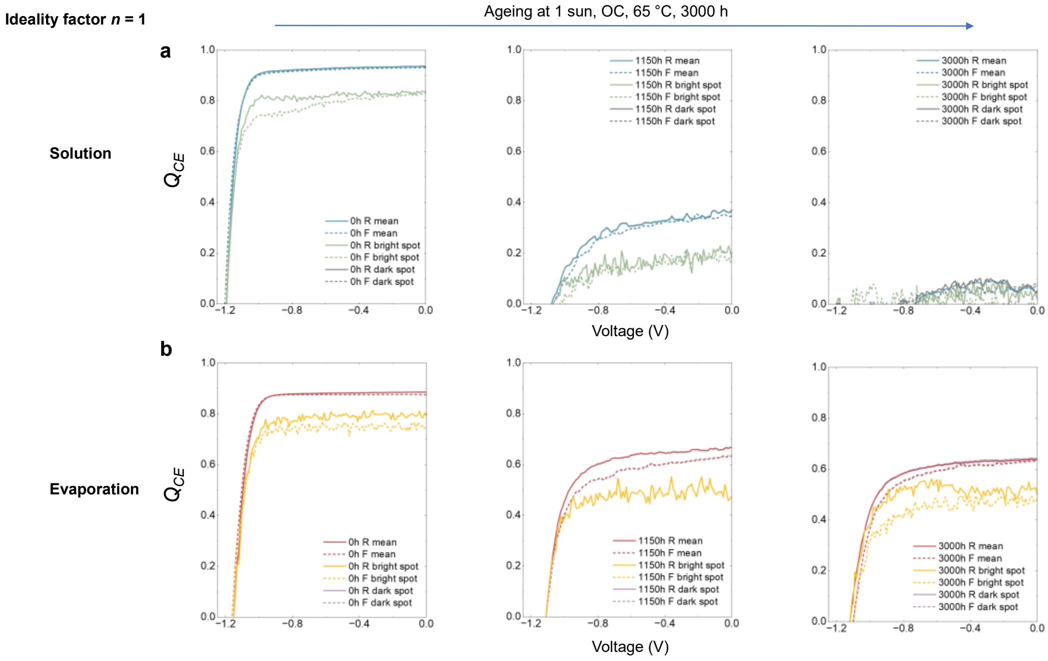

Supplementary Fig. 46. a–b, Ex-*JV* curves of the solution-processed cell (a) and evaporated cell (b) as shown in **Figs. 5a–d** and **Supplementary Figs. 54a–b** during 3000 hours of ageing under ISOS-L-2 ageing conditions: OC, at 65 ± 5 °C, and 1 sun full-spectrum illumination, in ambient (relative humidity in lab = 80–90%). The area-average, dark spot, bright spot-derived ex-*JV* curves are shown separately. The Q_{CE} was calculated when the ideality factor n was assumed as 1 at different bias voltages and various ageing times.

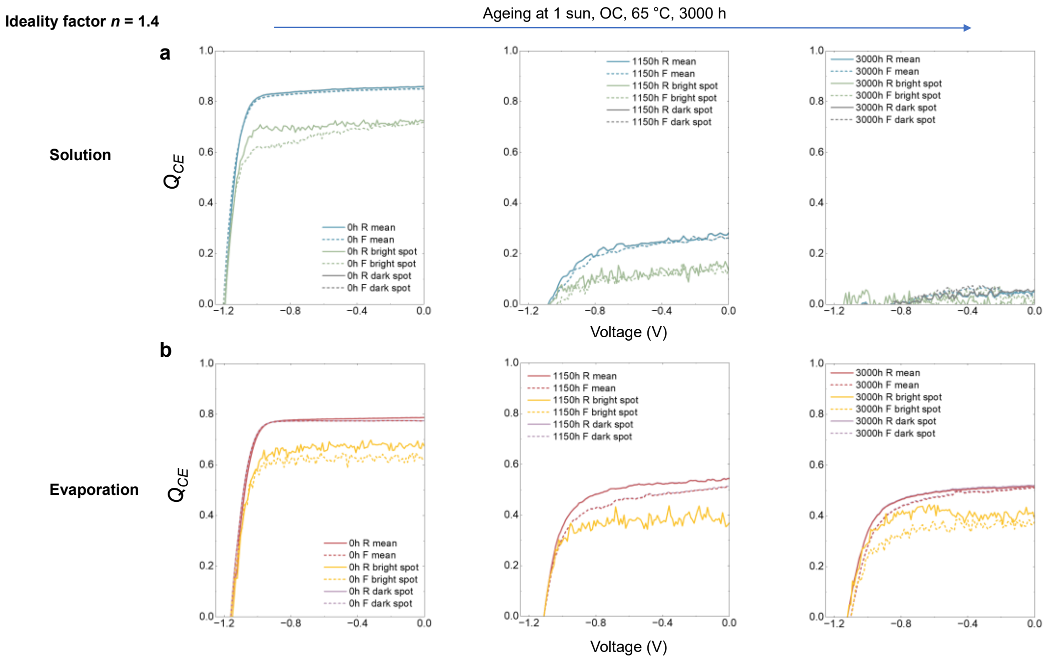

Supplementary Fig. 47. a–b, Ex-*JV* curves of the solution-processed cell (a) and evaporated cell (b) as shown in **Figs. 5a–d** and **Supplementary Figs. 54a–b** during 3000 hours of ageing under ISOS-L-2 ageing conditions: OC, at 65 ± 5 °C, and 1 sun full-spectrum illumination, in ambient (relative humidity in lab = 80–90%). The area-average, dark spot, bright spot-derived ex-*JV* curves are shown separately. The Q_{CE} was calculated when the ideality factor n was assumed as 1.4 at different bias voltages and various ageing times.

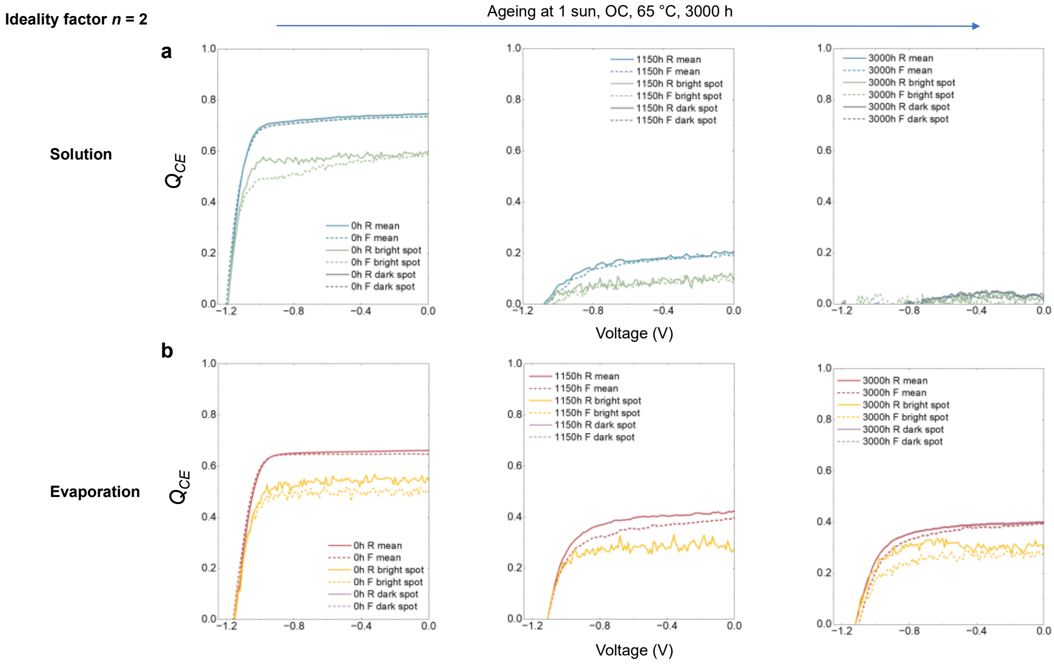

Supplementary Fig. 48. a–b, Ex-*JV* curves of the solution-processed cell (a) and evaporated cell (b) as shown in **Figs. 5a–d** and **Supplementary Figs. 54a–b** during 3000 hours of ageing under ISOS-L-2 ageing conditions: OC, at 65 ± 5 °C, and 1 sun full-spectrum illumination, in ambient (relative humidity in lab = 80–90%). The area-average, dark spot, bright spot-derived ex-*JV* curves are shown separately. The Q_{CE} was calculated when the ideality factor n was assumed as 2 at different bias voltages and various ageing times.

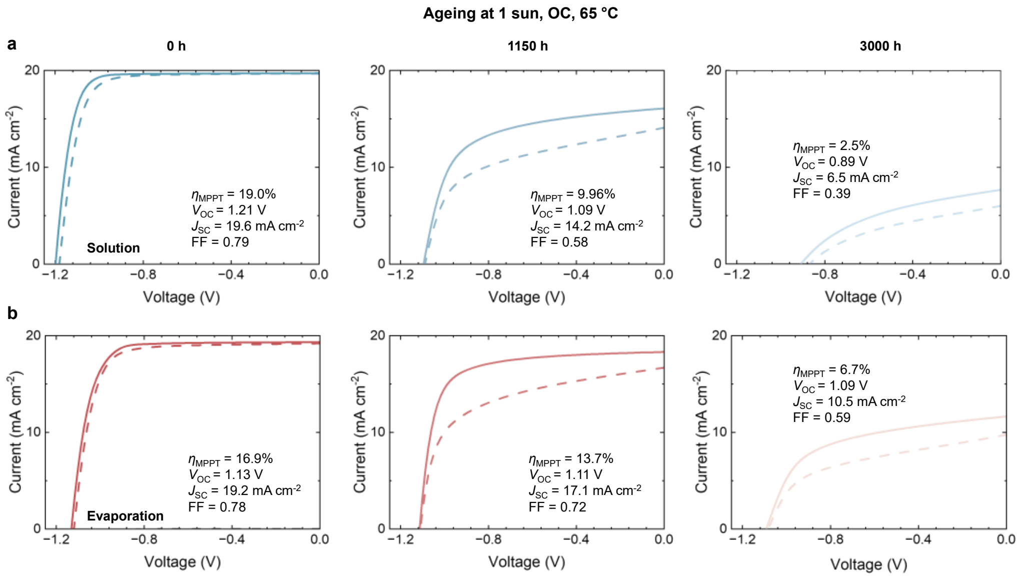

Supplementary Fig. 49. a–b, Evolution of the real *J–V* curves of the corresponding solution-processed (a) and evaporated cells (b) with ALD SnO_2 as shown in **Fig. 5** and **Supplementary Fig. 54** before and after 3000 h of ageing under ISOS-L-2 ageing conditions: OC, at 65 ± 5 °C, and 1 sun full-spectrum illumination, in ambient (relative humidity in lab = 80–90%).

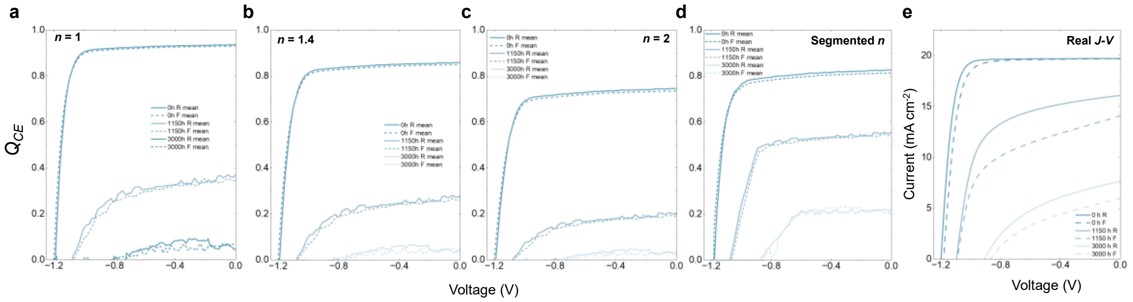

Supplementary Fig. 50. a–c, Ex-*JV* curves constructed with various ideality factors: $n = 1$ (a), 1.4 (b), or 2 (c) of the solution-processed cell as shown in **Figs. 5** and **Supplementary Fig. 54** during 3000 hours of ageing under ISOS-L-2 ageing conditions: OC, at 65 ± 5 °C, and 1 sun full-spectrum illumination, in ambient (relative humidity in lab = 80–90%). **d,** Ex-*JV* curves constructed with segmented ideality factors *n(V)* that varied with ageing times and bias conditions. The Q_{CE} were calculated using various *n(V)*. The n_{OC} and n_{SC} were set as 1.16, 1.26 for 0 h; 1.62, 1.42 for 1150 h; and 1.5, 1.42 for 3000 h, respectively. **e,** Real *J–V* curves of the corresponding solution-processed cells shown in **a–d**. We estimated 100% internal quantum efficiency (IQE) from the detailed balance limit for a 1.685-eV absorber under AM1.5G, giving J_{SC} of ~ 22.5 mA cm^{-2}.[74] This value is used as a fixed $Q_{CE} = 1$ reference in the ex-*JV* analysis. The real *J–V* curves are rescaled accordingly.

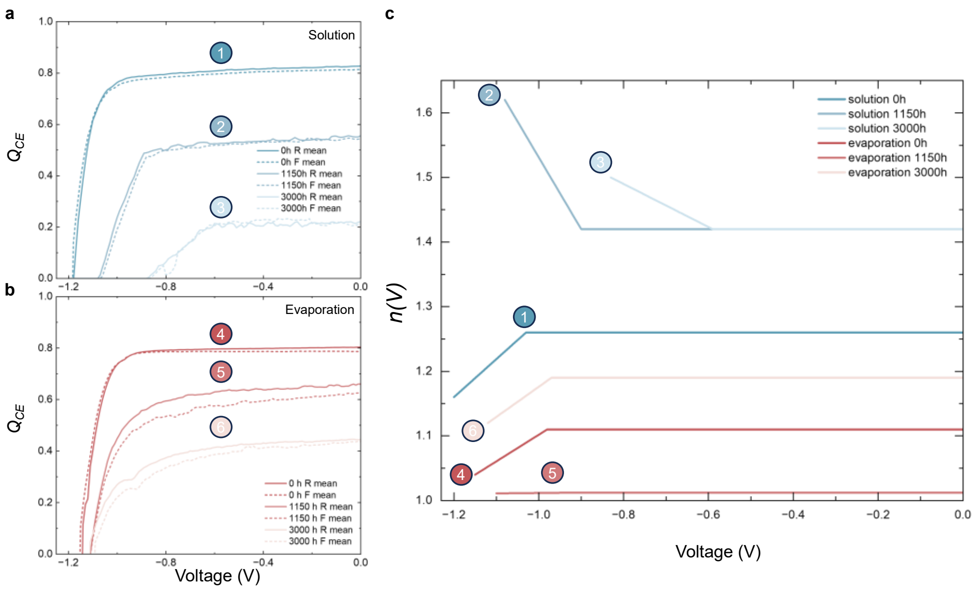

Supplementary Fig. 51. a–b, Ex-*JV* curves for solution-processed (a) and evaporated (b) cells as shown in **Figs. 5e–h** and **Supplementary Figs. 54c–d** during 3000 hours of ageing under ISOS-L-2 ageing conditions: OC, at 65 ± 5 °C, and 1 sun full-spectrum illumination, in ambient (relative humidity in lab = 80–90%). The Q_{CE} were calculated with segmented ideality factors *n(V)* that varied with ageing times and bias conditions. **c,** Plot of *n(V)* against bias voltage that were used to construct the ex-*JV* curves in (a) and (b).

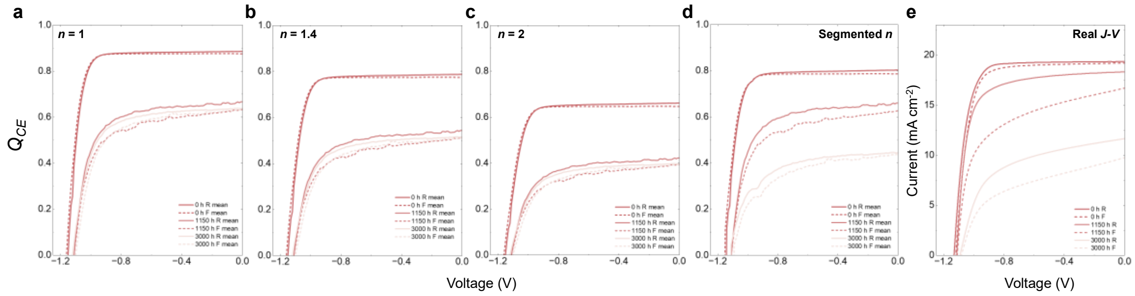

Supplementary Fig. 52. a–c, Ex-*JV* curves constructed with various ideality factors: $n = 1$ (a), 1.4 (b), or 2 (c) of the evaporated cell as shown in **Figs. 5** and **Supplementary Fig. 54** during 3000 hours of ageing under ISOS-L-2 ageing conditions: OC, at 65 ± 5 °C, and 1 sun full-spectrum illumination, in ambient (relative humidity in lab = 80–90%). **d,** Ex-*JV* curves constructed with segmented ideality factors *n(V)* that varied with ageing times and bias conditions. The Q_{CE} were calculated using various *n(V)*. The n_{OC} and n_{SC} were assumed as 1.04, 1.11 for 0 h; 1.011, 1.012 for 1150 h; and 1.12, 1.19 for 3000 h, respectively. We estimated 100% IQE from the detailed balance limit for a 1.67-eV absorber under AM1.5G, giving J_{SC} of ~ 23 mA cm^{-2}.[74] This value is used as a fixed $Q_{CE} = 1$ reference in the ex-*JV* analysis. The real *J–V* curves are rescaled accordingly.

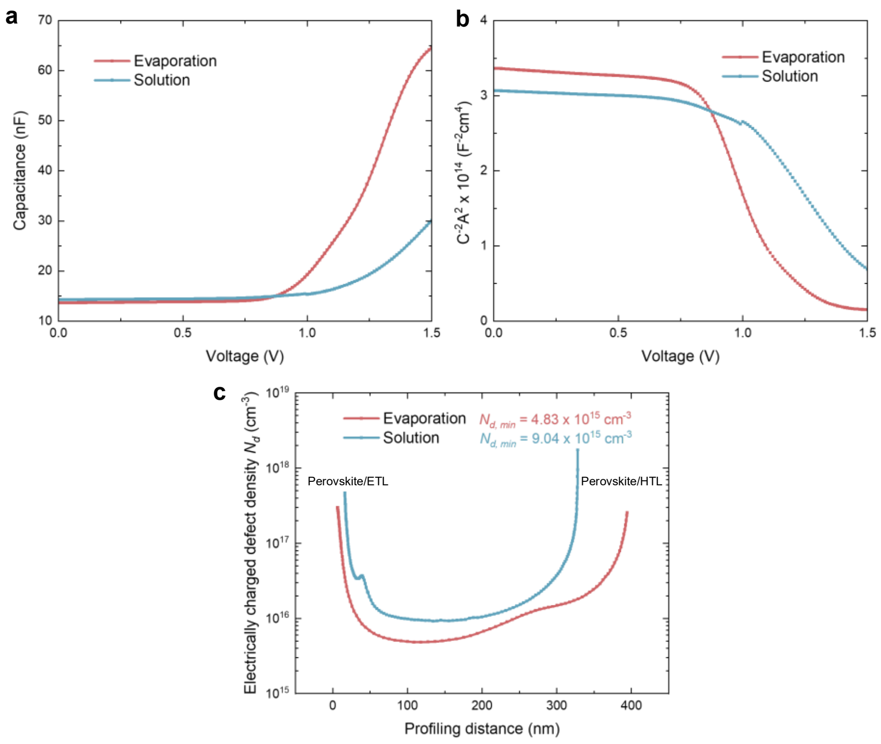

Supplementary Fig. 53. a–c, Capacitance-voltage (*C*-*V*) profiling results (a), Mott-Schottky plot (b) and electrically charged defect density profile (c) of the evaporated and solution-processed cells. The electrically charged defect density profile is calculated from Mott-Schottky analysis as a function of estimated depletion depth from the perovskite/ETL interface to the perovskite/HTL interface. $N_{d,min}$ is the minimum electrically charged defect density point in the profile which is located within the bulk. The resulting electrically charged defect density distributions revealed electrically charged defects, including trap states, primarily at the perovskite/charge transport layer interfaces, consistent with prior reports.[71–73] Crucially, the co-evaporated perovskite/HTL interface and perovskite/ETL interface exhibited defect densities approximately 10 times lower and half that of the solution-processed counterpart, respectively, indicating significantly reduced interfacial losses in the co-evaporated devices.

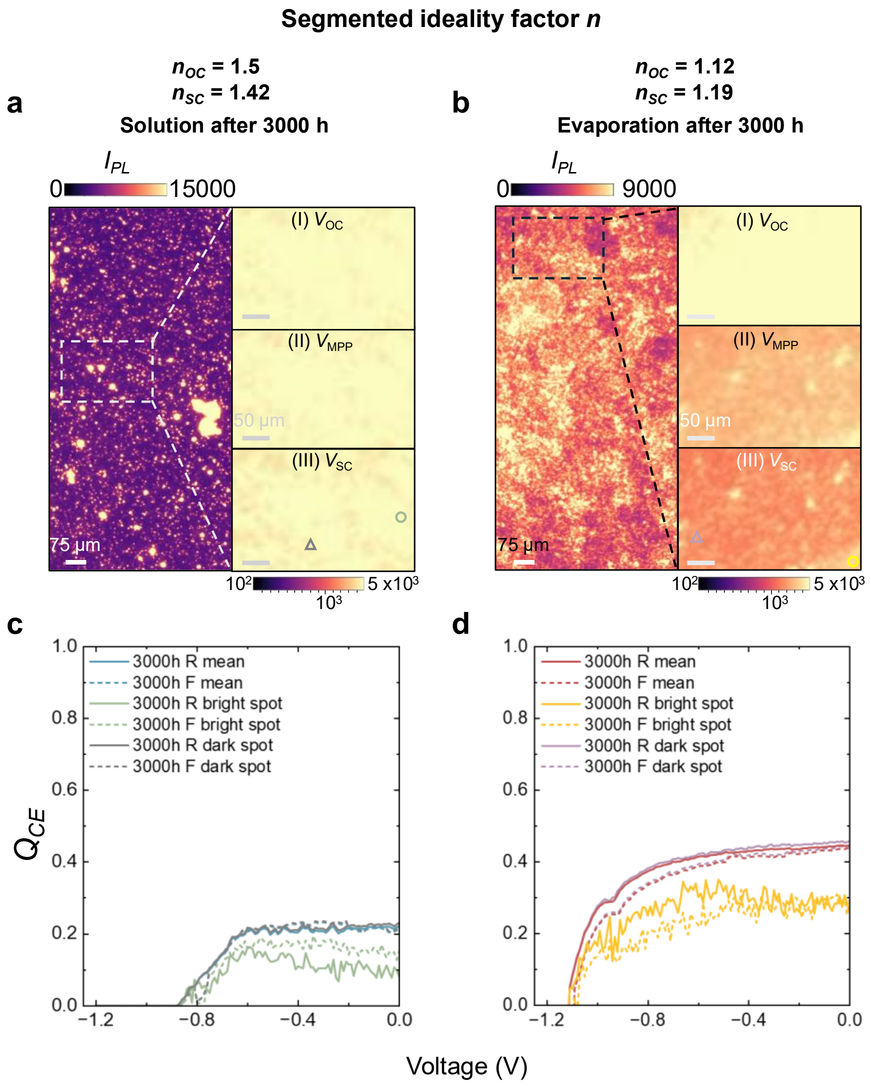

Supplementary Fig. 54. a–b, Broadband PL maps of the corresponding solution-processed (a) and evaporated (b) perovskite solar cells as shown in **Fig. 5** after 3000 hours of ageing under ISOS-L-2 ageing conditions: OC, at 65 ± 5 °C, and 1 sun full-spectrum illumination, in ambient (relative humidity in lab = 80–90%). For solution cell: green circle: bright spot with high I_{PL}; grey triangle: dark spot with low I_{PL}. For evaporated cell: yellow circle: bright spot with high I_{PL}; purple triangle: dark spot with low I_{PL}. A region is marked with dashed square to show the PL heterogeneity at V_{OC} (I), V_{MPP} (II), and V_{SC} (III) (right). The PL maps at V_{OC} (I), V_{MPP} (II) and V_{SC} (III) are plotted in log scale to reveal the heterogeneous features at low I_{PL}. Scale bars: 75 µm (left panel) and 50 µm (right panel). **c-d,** Ex-*JV* curves of the corresponding solution-processed (c) and evaporated (d) perovskite solar cells shown in (a) and (b), respectively. The area-average, dark spot, bright spot-derived ex-*JV* curves are shown separately. The area-average, dark spot, bright spot-derived ex-*JV* curves are shown separately. Ex-*JV* curves were constructed with segmented ideality factors *n(V)* that varied with ageing times and bias conditions. The Q_{CE} were calculated using various *n(V)*. The n_{OC} and n_{SC} were assumed as 1.5, 1.42 (c), 1.12, 1.19 (d), respectively.

References

1. Dong, Z. *et al.* Intermediate phase evolution for stable and oriented evaporated wide-bandgap perovskite solar cells. *Nat. Mater.* 1–8 (2025).
2. McMeekin, D. P. *et al.* Intermediate-phase engineering via dimethylammonium cation additive for stable perovskite solar cells. *Nat. Mater.* **22**, 73–83 (2023).
3. Lin, Y.-H. *et al.* A piperidinium salt stabilizes efficient metal-halide perovskite solar cells. *Science* **369**, 96–102 (2020).
4. Liu, Z. *et al.* Strained heterojunction enables high-performance, fully textured perovskite/silicon tandem solar cells. *Joule* **8**, 2834–2850 (2024).
5. Zhou, S. *et al.* Reactive passivation of wide-bandgap organic-inorganic perovskites with benzylamine. *J. Am. Chem. Soc.* **146**, 27405–27416 (2024).
6. Wang, Z. *et al.* Regulation of wide bandgap perovskite by rubidium thiocyanate for efficient silicon/perovskite tandem solar cells. *Adv. Mater.* **36**, e2407681 (2024).
7. Zhao, K. *et al.* peri-Fused polyaromatic molecular contacts for perovskite solar cells. *Nature* **632**, 301–306 (2024).
8. Chen, Y. *et al.* Nuclei engineering for even halide distribution in stable perovskite/silicon tandem solar cells. *Science* **385**, 554–560 (2024).
9. Meng, H. *et al.* Inhibition of halide oxidation and deprotonation of organic cations with dimethylammonium formate for air-processed p–i–n perovskite solar cells. *Nat. Energy* **9**, 536–547 (2024).
10. Zhu, Y. *et al.* Fluorinated pyrimidine bridged buried interface for stable and efficient wide-bandgap perovskite solar cells. *Adv. Funct. Mater.* (2024) doi:10.1002/adfm.202417310.
11. Yao, Y. *et al.* Oriented wide-bandgap perovskites for monolithic silicon-based tandems with over 1000 hours operational stability. *Nat. Commun.* **16**, 40 (2025).
12. Škorjanc, V. *et al.* Seed layers for wide-band gap coevaporated perovskite solar cells: CsCl regulates band gap and reduces process variability. *ACS Energy Lett.* **9**, 5639–5646 (2024).
13. Fang, Z. *et al.* Surface reconstruction of wide-bandgap perovskites enables efficient perovskite/silicon tandem solar cells. *Nat. Commun.* **15**, 10554 (2024).
14. Li, X. *et al.* Strain regulation of mixed-Halide perovskites enables high-performance wide-bandgap photovoltaics. *Adv. Mater.* **36**, e2401103 (2024).
15. Liu, J. *et al.* Perovskite/silicon tandem solar cells with bilayer interface passivation. *Nature* **635**, 596–603 (2024).
16. Subbiah, A. S. *et al.* Efficient blade-coated perovskite/silicon tandems via interface engineering. *Joule* (2024) doi:10.1016/j.joule.2024.09.014.
17. Zhou, X. *et al.* Regulating crystallization for pure-iodide 1.68 eV bandgap perovskite solar cells with a fill factor over 86. *ACS Nano* **19**, 11187–11196 (2025).

18. Gil-Escrig, L. *et al.* Efficient wide-bandgap mixed-cation and mixed-Halide perovskite solar cells by vacuum deposition. *ACS Energy Lett.* **6**, 827–836 (2021).
19. Yang, Y. *et al.* Activating halogen circulation enables efficient and stable wide-bandgap mixed-Halide perovskite solar cells. *Adv. Mater.* **37**, e2416513 (2025).
20. Wang, L. *et al.* Highly efficient monolithic perovskite/TOPCon silicon tandem solar cells enabled by "Halide locking." *Adv. Mater.* **37**, e2416150 (2025).
21. Ye, Q. *et al.* Suppressing charge recombination in a methylammonium-free wide-bandgap perovskite film for high-performance and stable perovskite solar cells. *Energy Environ. Sci.* **17**, 5866–5875 (2024).
22. Yang, G. *et al.* Defect engineering in wide-bandgap perovskites for efficient perovskite–silicon tandem solar cells. *Nat. Photonics* **16**, 588–594 (2022).
23. Li, J. *et al.* Homogenizing the halogen distribution via a multifunctional fluorine-containing additive toward high-performance inverted wide-bandgap perovskite solar cells. *Adv. Funct. Mater.* 2422175 (2025).
24. Huang, T. *et al.* Rational heterostructure stacking enables 23% wide-bandgap perovskite solar cells by side-reaction inhibition. *Energy Environ. Sci.* **17**, 5984–5992 (2024).
25. Al-Ashouri, A. *et al.* Monolithic perovskite/silicon tandem solar cell with >29% efficiency by enhanced hole extraction. *Science* **370**, 1300–1309 (2020).
26. Wang, R. *et al.* Custom-tailored solvent engineering for efficient wide-bandgap perovskite solar cells with a wide processing window and low V_{OC} losses. *Energy Environ. Sci.* **17**, 2662–2669 (2024).
27. Li, S. *et al.* A generic strategy to stabilize wide bandgap perovskites for efficient tandem solar cells. *Adv. Mater.* **36**, e2307701 (2024).
28. Jin, Y. *et al.* Recrystallizing sputtered NiO_x for improved hole extraction in perovskite/silicon tandem solar cells. *Adv. Energy Mater.* **15**, 2403911 (2025).
29. Luo, X. *et al.* Efficient perovskite/silicon tandem solar cells on industrially compatible textured silicon. *Adv. Mater.* **35**, e2207883 (2023).
30. Yan, S. *et al.* A templating approach to controlling the growth of coevaporated Halide perovskites. *ACS Energy Lett.* **8**, 4008–4015 (2023).
31. Lohmann, K. B. *et al.* Solvent-free method for defect reduction and improved performance of p-i-n vapor-deposited perovskite solar cells. *ACS Energy Lett.* **7**, 1903–1911 (2022).
32. Shen, X. *et al.* Chloride-based additive engineering for efficient and stable wide-bandgap perovskite solar cells. *Adv. Mater.* **35**, e2211742 (2023).
33. Xu, J. *et al.* Triple-halide wide-band gap perovskites with suppressed phase segregation for efficient tandems. *Science* **367**, 1097–1104 (2020).

34. He, J. *et al.* Synchronous elimination of excess photoinstable PbI2 and interfacial band mismatch for efficient and stable perovskite solar cells. *Angew. Chem. Int. Ed Engl.* **63**, e202315233 (2024).
35. Zhong, H. *et al.* Suppressing the crystallographic disorders induced by excess PbI2 to achieve trade-off between efficiency and stability for PbI2-rich perovskite solar cells. *Nano Energy* **105**, 108014 (2023).
36. Gil-Escrig, L. *et al.* Efficient and thermally stable wide bandgap perovskite solar cells by dual-source vacuum deposition. *Adv. Funct. Mater.* **33**, (2023).
37. Chin, X. Y. *et al.* Interface passivation for 31.25%-efficient perovskite/silicon tandem solar cells. *Science* **381**, 59–63 (2023).
38. Susic, I., Gil-Escrig, L., Palazon, F., Sessolo, M. & Bolink, H. J. Quadruple-cation wide-bandgap perovskite solar cells with enhanced thermal stability enabled by vacuum deposition. *ACS Energy Lett.* **7**, 1355–1363 (2022).
39. Zhou, Y. *et al.* Effect of solvent residue in the thin-film fabrication on perovskite solar cell performance. *ACS Appl. Mater. Interfaces* **14**, 28729–28737 (2022).
40. Mo, K. *et al.* Minimizing DMSO residues in perovskite films for efficient and long-term stable solar cells. *Adv. Energy Mater.* (2025) doi:10.1002/aenm.202404538.
41. Zhu, H. *et al.* Long-term operating stability in perovskite photovoltaics. *Nat. Rev. Mater.* **8**, 569–586 (2023).
42. Pradhan, N. Why do perovskite nanocrystals form nanocubes and how can their facets be tuned? A perspective from synthetic prospects. *ACS Energy Lett.* **6**, 92–99 (2021).
43. Ma, C., Grätzel, M. & Park, N.-G. Facet engineering for stable, efficient perovskite solar cells. *ACS Energy Lett.* **7**, 3120–3128 (2022).
44. Liu, Z. *et al.* All-perovskite tandem solar cells achieving >29% efficiency with improved (100) orientation in wide-bandgap perovskites. *Nat. Mater.* **24**, 252–259 (2025).
45. Ran, C., Xu, J., Gao, W., Huang, C. & Dou, S. Defects in metal triiodide perovskite materials towards high-performance solar cells: origin, impact, characterization, and engineering. *Chem. Soc. Rev.* **47**, 4581–4610 (2018).
46. Zheng, X. *et al.* Managing grains and interfaces via ligand anchoring enables 22.3%-efficiency inverted perovskite solar cells. *Nat. Energy* **5**, 131–140 (2020).
47. Kim, D. *et al.* Probing facet-dependent surface defects in $MAPbI_3$ perovskite single crystals. *J. Phys. Chem. C Nanomater. Interfaces* **123**, 14144–14151 (2019).
48. Zheng, G. *et al.* Manipulation of facet orientation in hybrid perovskite polycrystalline films by cation cascade. *Nat. Commun.* **9**, 2793 (2018).

49. Fesquet, L. *et al.* Modification of textured silicon wafer surface morphology for fabrication of heterojunction solar cell with open circuit voltage over 700 mV. in *2009 34th IEEE Photovoltaic Specialists Conference (PVSC)* 000754–000758 (IEEE, 2009).
50. Oliver, R. D. J. *et al.* Understanding and suppressing non-radiative losses in methylammonium-free wide-bandgap perovskite solar cells. *Energy Environ. Sci.* **15**, 714–726 (2022).
51. Stolterfoht, M. *et al.* The impact of energy alignment and interfacial recombination on the internal and external open-circuit voltage of perovskite solar cells. *Energy Environ. Sci.* **12**, 2778–2788 (2019).
52. Wang, J. *et al.* Exposing binding-favourable facets of perovskites for tandem solar cells. *Energy Environ. Sci.* **18**, 7680–7694 (2025).
53. Li, M. *et al.* Molecularly engineered self-assembled monolayers as effective hole-selective layers for organic solar cells. *ACS Appl. Energy Mater.* **7**, 1306–1312 (2024).
54. Snaith, H. J. & Hacke, P. Enabling reliability assessments of pre-commercial perovskite photovoltaics with lessons learned from industrial standards. *Nat. Energy* **3**, 459–465 (2018).
55. Boyd, C. C., Cheacharoen, R., Leijtens, T. & McGehee, M. D. Understanding degradation mechanisms and improving stability of perovskite photovoltaics. *Chem. Rev.* **119**, 3418–3451 (2019).
56. Hu, S. *et al.* Steering perovskite precursor solutions for multijunction photovoltaics. *Nature* **639**, 93–101 (2025).
57. Liu, A. *et al.* Roadmap on metal-halide perovskite semiconductors and devices. *Materials Today Electronics* **11**, 100138 (2025).
58. Jiang, Q. *et al.* Towards linking lab and field lifetimes of perovskite solar cells. *Nature* **623**, 313–318 (2023).
59. Katahara, J. K. & Hillhouse, H. W. Quasi-Fermi level splitting and sub-bandgap absorptivity from semiconductor photoluminescence. *J. Appl. Phys.* **116**, 173504 (2014).
60. Frohna, K. *et al.* The impact of interfacial quality and nanoscale performance disorder on the stability of alloyed perovskite solar cells. *Nat. Energy* **10**, 66–76 (2025).
61. Nayak, P. K., Mahesh, S., Snaith, H. J. & Cahen, D. Photovoltaic solar cell technologies: analysing the state of the art. *Nat. Rev. Mater.* **4**, 269–285 (2019).
62. Mahesh, S. *et al.* Revealing the origin of voltage loss in mixed-halide perovskite solar cells. *Energy Environ. Sci.* **13**, 258–267 (2020).
63. Lin, Y.-H. *et al.* Bandgap-universal passivation enables stable perovskite solar cells with low photovoltage loss. *Science* **384**, 767–775 (2024).
64. Dasgupta, A. *et al.* Visualizing macroscopic inhomogeneities in perovskite solar cells. *ACS Energy Lett.* **7**, 2311–2322 (2022).
65. Sze, S. M. & Ng, K. K. *Physics of Semiconductor Devices: Sze/Physics*. (Wiley-Blackwell, Chichester, England, 2006).

66. Calado, P. *et al.* Identifying dominant recombination mechanisms in perovskite solar cells by measuring the transient ideality factor. *Phys. Rev. Appl.* **11**, (2019).
67. Foster, J. M., Snaith, H. J., Leijtens, T. & Richardson, G. A model for the operation of perovskite based hybrid solar cells: Formulation, analysis, and comparison to experiment. *SIAM J. Appl. Math.* **74**, 1935–1966 (2014).
68. Wolff, C. M., Caprioglio, P., Stolterfoht, M. & Neher, D. Nonradiative recombination in perovskite solar cells: The role of interfaces. *Adv. Mater.* **31**, e1902762 (2019).
69. Stolterfoht, M. *et al.* Approaching the fill factor Shockley–Queisser limit in stable, dopant-free triple cation perovskite solar cells. *Energy Environ. Sci.* **10**, 1530–1539 (2017).
70. Caprioglio, P. *et al.* On the relation between the open-circuit voltage and quasi-Fermi level splitting in efficient perovskite solar cells. *Adv. Energy Mater.* **9**, 1901631 (2019).
71. Ni, Z. *et al.* Resolving spatial and energetic distributions of trap states in metal halide perovskite solar cells. *Science* **367**, 1352–1358 (2020).
72. Ravishankar, S., Unold, T. & Kirchartz, T. Comment on "Resolving spatial and energetic distributions of trap states in metal halide perovskite solar cells." *Science (New York, N.Y.)* vol. 371 eabd8014 (2021).
73. Ni, Z., Xu, S. & Huang, J. Response to Comment on "Resolving spatial and energetic distributions of trap states in metal halide perovskite solar cells." *Science (New York, N.Y.)* vol. 371 eabd8598 (2021).
74. Shockley, W. & Queisser, H. J. Detailed balance limit of efficiency of *p-n* junction solar cells. *J. Appl. Phys.* **32**, 510–519 (1961).
75. Abzieher, T. *et al.* Vapor phase deposition of perovskite photovoltaics: short track to commercialization? *Energy Environ. Sci.* **17**, 1645–1663 (2024).
76. Ablekim, T. *et al.* Thin-film solar cells with 19% efficiency by thermal evaporation of CdSe and CdTe. *ACS Energy Lett.* **5**, 892–896 (2020).
77. Feurer, T. *et al.* Progress in thin film CIGS photovoltaics – Research and development, manufacturing, and applications: Progress in thin film CIGS photovoltaics. *Prog. Photovolt.* **25**, 645–667 (2017).
78. Kim, S.-H. *et al.* A study on thin film uniformity in a roll-to-roll thermal evaporation system for flexible OLED applications. *Int. J. Precis. Eng. Manuf.* **18**, 1111–1117 (2017).
79. Jacobsson, T. J. *et al.* Unreacted PbI2 as a double-edged sword for enhancing the performance of perovskite solar cells. *J. Am. Chem. Soc.* **138**, 10331–10343 (2016).
80. Kim, M. *et al.* Methylammonium chloride induces intermediate phase stabilization for efficient perovskite solar cells. *Joule* **3**, 2179–2192 (2019).
81. Macpherson, S. *et al.* Local nanoscale phase impurities are degradation sites in halide perovskites. *Nature* **607**, 294–300 (2022).

82. Susic, I. *et al.* Pure iodide multication wide bandgap perovskites by vacuum deposition. *ACS Mater. Lett.* **5**, 3299–3305 (2023).
83. Susic, I. *et al.* Combinatorial vacuum-deposition of wide bandgap perovskite films and solar cells. *Adv. Mater. Interfaces* **10**, 2202271 (2023).
84. Al-Ashouri, A. *et al.* Conformal monolayer contacts with lossless interfaces for perovskite single junction and monolithic tandem solar cells. *Energy Environ. Sci.* **12**, 3356–3369 (2019).
85. Niu, X. *et al.* Anion confinement for homogeneous mixed Halide perovskite film growth by electrospray. *Adv. Mater.* **35**, e2305822 (2023).
86. Liu, J. *et al.* Efficient and stable perovskite-silicon tandem solar cells through contact displacement by MgFx. *Science* **377**, 302–306 (2022).
87. Kan, C. *et al.* Efficient and stable perovskite-silicon tandem solar cells with copper thiocyanate-embedded perovskite on textured silicon. *Nat. Photonics* **19**, 63–70 (2025).
88. Duan, C. *et al.* Scalable fabrication of wide-bandgap perovskites using green solvents for tandem solar cells. *Nat. Energy* **10**, 318–328 (2024).
89. Mariotti, S. *et al.* Interface engineering for high-performance, triple-halide perovskite-silicon tandem solar cells. *Science* **381**, 63–69 (2023).
90. Scheler, F. *et al.* Correlation of band bending and ionic losses in 1.68 eV wide band gap perovskite solar cells. *Adv. Energy Mater.* (2024) doi:10.1002/aenm.202404726.
91. Dai, X. *et al.* Efficient monolithic all-perovskite tandem solar modules with small cell-to-module derate. *Nat. Energy* **7**, 923–931 (2022).
92. Li, Z. *et al.* Stabilized hole-selective layer for high-performance inverted p-i-n perovskite solar cells. *Science* **382**, 284–289 (2023).
93. Kim, S.-W. *et al.* Reducing humidity dependency of ambient-air-processed wide-bandgap inverted perovskite solar cells. *ACS Energy Lett.* **8**, 4777–4781 (2023).
94. Zhang, Z. *et al.* Semitransparent perovskite solar cells with an evaporated ultra-thin perovskite absorber. *Adv. Funct. Mater.* (2023) doi:10.1002/adfm.202307471.
95. Liu, Z. *et al.* Reducing perovskite/C60 interface losses via sequential interface engineering for efficient perovskite/silicon tandem solar cell. *Adv. Mater.* **36**, e2308370 (2024).
96. Su, G. *et al.* Crystallization regulation and defect passivation for efficient inverted wide-bandgap perovskite solar cells with over 21% efficiency. *Adv. Energy Mater.* **14**, (2024).
97. Huo, X. *et al.* Unraveling the relationship between the phenethylammonium-induced 2D phase on the perovskite surface and inverted wide bandgap perovskite solar cell performance. *Energy Environ. Sci.* **17**, 8658–8669 (2024).
