# Supplementary material for: Crystal-facet-directed all-vacuum-deposited perovskite solar cells
Source: Nat Mater. 2026 Feb 23;25(6):999–1010. doi: 10.1038/s41563-026-02494-w (PMC13236600; doi:10.1038/s41563-026-02494-w)
Supplement: Supplementary file 2 — Reporting Summary [file 41563_2026_2494_MOESM2_ESM.pdf]

nature research

Corresponding author(s): Henry Snaith & Yen-Hung Lin

☐ Initial submission ☐ Revised version ☑ Final submission

Solar Cells Reporting Summary

Nature Portfolio wishes to improve the reproducibility of the work that we publish. This form is intended for publication with all accepted papers reporting the characterization of photovoltaic devices and provides structure for consistency and transparency in reporting. Some list items might not apply to an individual manuscript, but all fields must be completed for clarity.

For further information on Nature Research policies, including our data availability policy, see Authors & Referees.

Please check the following details are reported in the manuscript, and provide a brief description or explanation where applicable.

Area of the tested solar cells — ☑ Yes ☐ No

Report the area of the tested solar cells.

Aperture areas used in the study are 1 cm² (Fig. 3,S1,S12,S16-17,S22,S25, S27, S29, S31, S32) and 0.25 cm² (Fig 1e,3,S1,S12,S13-15,S20,S22, S25

Method used to determine the device area — ☑ Yes ☐ No

Provide a description of the method and state where this information can be found in the text.

Defined by the shadow mask.

2. Current-voltage characterization

Current density-voltage (J-V) plots in both forward and backward direction — ☑ Yes ☐ No

Fig. 1e, 3a, S13-17, S26-29, S31-32, S39, S45, S49.

Voltage scan conditions — ☑ Yes ☐ No

Provide a description of the measurement conditions (e.g. scan direction, speed, dwell times).

The scan rate was approximately 0.1-0.3 V/s for both scan directions.

Test environment — ☑ Yes ☐ No

Provide a description of the test conditions (e.g. characterization temperature, atmosphere, humidity).

Solar cells were measured in ambient air (relative humidity in the lab = 40 -

Protocol for preconditioning of the device before its characterization — ☑ Yes ☐ No

Provide a description of the protocol.

No preconditioning protocol was used.

Stability of the J-V characteristic — ☑ Yes ☐ No

Provide a description of the method used. The stability of the J-V characteristic can be verified with time evolution of the maximum power point or with the photocurrent at maximum power point; see ref. 5 for details.

Stability of the J-V characteristics were performed by tracking the maximum power point (Fig. 3b,3c,3d,3g,S1,S12-17,S22,S25-29,S31-32,S39-40,S45, S49) or stabilised steady state (S1,S12-17,S22,S25-29,S31-32,S39-40, S45,S49)

Description of the unusual behaviour observed during the characterization — ☑ Yes ☐ No

Provide a description of hysteresis or any other unusual behaviour observed during the characterization.

Negligible hysteresis in single junction cells; slight hysteresis in perovskite-on-silicon tandem cells.

Related experimental data — ☑ Yes ☐ No

Provide a description of the related experimental data.

Fig. 1e,3a,S12-17,S31-32,S39,S45,S49.

External quantum efficiency (EQE) or incident photons to current efficiency (IPCE) — ☑ Yes ☐ No

Provide a description of the technique used.

EQE shown in Fig.S20 was measured as detailed in Methods.

A comparison between the integrated response under the standard reference spectrum and the response measure under the simulator — ☑ Yes ☐ No

The integrated short-circuit current from the standard AM1.5G spectrum matches the Jsc measured from the J-V measurements within 5%.

For tandem solar cells, the bias illumination and bias voltage used for each subcell

☐ Yes
☑ No

Provide a description of the measurement conditions.

5. Calibration

Light source and reference cell or sensor used for the characterization

☑ Yes
☐ No

Provide a description of the light source and reference cell or sensor.

AM1.5G solar irridiance was generated by a Wavelabs SINUS-220 solar simulator and calibrated with WPVS reference cell (monocrystalline silicon solar cell, provided and calibrated by Fraunhofer ISE) matched its 1-sun certified value.

Confirmation that the reference cell was calibrated and certified

☑ Yes
☐ No

Identify the independent certification laboratory.

he solar simulator was calibrated with WPVS reference cell (monocrystalline ilicon solar cell, provided and certified by Fraunhofer ISE) matched its 1-sun

Calculation of spectral mismatch between the reference cell and the devices under test

☐ Yes
☑ No

Provide a value of the spectral mismatch and/or a description of how it has been taken into account in the measurements.

Estimated mismatch factor is less than 1 and was hence not applied.

6. Mask/aperture

Size of the mask/aperture used during testing

☑ Yes
☐ No

Report the size of the mask/aperature.

Mask size is 0.25 cm2 and 1 cm^2.

Variation of the measured short-circuit current density with the mask/aperture area

☑ Yes
☐ No

Report the difference in the short-circuit current density values measured with the mask and aperture area.

All short-circuit current were measured with masks on.

7. Performance certification

Identity of the independent certification laboratory that confirmed the photovoltaic performance

☑ Yes
☐ No

Identify the independent certification laboratory.

NPVM (Chinese national PV industry measurement and testing center)

A copy of any certificate(s)

☑ Yes
☐ No

Certificate copies should be provided in the Supplementary information. Please state the supplementary item number.

Fig.S14-15

8. Statistics

Number of solar cells tested

☑ Yes
☐ No

Report how many solar cells have been tested, specifying the number of individual substrates.

Over 250 devices were tested.

Statistical analysis of the device performance

☑ Yes
☐ No

ig. 3c, S1,S12, S22, S25. Number of cells tested were stated in the figure caption.

9. Long-term stability analysis

Type of analysis, bias conditions and environmental conditions

☑ Yes
☐ No

Provide a description of the type of analysis, bias conditions and environmental conditions (e.g. illumination type, temperature, atmosphere humidity, encapsulation method, preconditioning temperature, bias) for each long-term stability analysis carried out; see ref. 7 and 8 for details.

1. ISOS-L-2: Cells were encapsulated with epoxy glue and cover glass, and aged under open-circuit, full-spectrum simulated sunlight (0.76 sun), 75 ± 5 , in ambient air with 50–60% RH in lab (Fig. 3c-d, Fig.S25-29).
2. ISOS-L-2: Cells were encapsulated with epoxy glue and cover glass, and aged under open-circuit, full-spectrum simulated sunlight (1 sun), 65 ± 5 , in ambient air with 80–90% RH in lab (Fig. 4-5, Fig.S38-54).
3. Outdoor stability test: perovskite-on-silicon tandem cells were laminated between two sheets of glass with a polyolefin foil and a butyl rubber edge sealant, and aged at maximum power point tracking in Italy for 8 months. Variation in temperature and illumination are in main text (Fig. 3f,g).

1. Shrotriya, V. *et al*. Accurate measurement and characterization of organic solar cells. *Adv. Funct. Mater.* **16**, 2016–2023 (2006).
2. Dennler, G. *et al*. The value of values. *Mat. Today* **10**, 56 (2007).
3. Cravino, A., Schilinsky, P. & Brabec, C. J. Characterization of organic solar cells: the importance of device layout. *Adv. Funct. Mater.* **17**, 3906–3910 (2007).
4. Reese, M. O. *et al*. Consensus stability testing protocols for organic photovoltaic materials and devices. *Sol. Energ. Mat. Sol. C* **95**, 1253–1267 (2011).
5. Snaith H. .J. The perils of solar cell efficiency measurements. *Nat. Photon*. **6**, 337–340 (2012).
6. Luber, E. J. & Buriak, J. M. Reporting performance in organic photovoltaic devices. *ACS Nano* **7**, 4708–4714 (2013).
7. Snaith, H. J. *et al*. Anomalous hysteresis in perovskite solar cells. *J. Phys. Chem. Lett.* **5**, 1511–1515 (2014).
8. Grätzel M. The light and shade of perovskite solar cells. *Nat. Mat*. **13**, 838–842 (2014).
9. Zimmermann E. *et al*. Erroneous efficiency reports harm organic solar cell research. *Nat. Photon*. **8**, 669–672 (2014).
10. Beard M.C., Luther J.M. & Nozik A.J. The promise and challenge of nanostructured solar cells. *Nat. Nanotech*. **9**, 951–954 (2014).
11. Timmreck, R. *et al*. Characterization of tandem organic solar cells. *Nat. Photon.* **9**, 478–479 (2015).

A number of international committees develop industry standards on the characterization of photovoltaic technologies (for example ASTM-E44 and IEC-TC 82), which can provide guidance for academic research.

This checklist template is licensed under a Creative Commons Attribution 4.0 International License, which permits use, sharing, adaptation, distribution and reproduction in any medium or format, as long as you give appropriate credit to the original author(s) and the source, provide a link to the Creative Commons license, and indicate if changes were made. The images or other third party material in this article are included in the article's Creative Commons license, unless indicated otherwise in a credit line to the material. If material is not included in the article's Creative Commons license and your intended use is not permitted by statutory regulation or exceeds the permitted use, you will need to obtain permission directly from the copyright holder. To view a copy of this license, visit http://creativecommons.org/licenses/by/4.0/
