# Supplementary figures and images for: Crystal-facet-directed all-vacuum-deposited perovskite solar cells
Source: Nat Mater. 2026 Feb 23;25(6):999–1010. doi: 10.1038/s41563-026-02494-w (PMC13236600; doi:10.1038/s41563-026-02494-w)

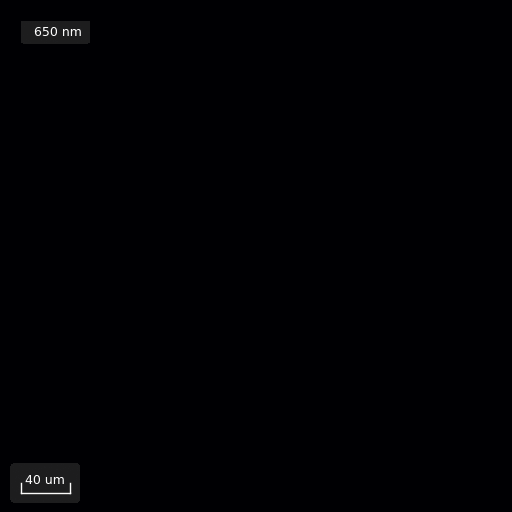

Supplement: Supplementary file 3 — Hyperspectral data cube of an all-vacuum-deposited PSC before ageing under ISOS-L-2 protocol. [file 41563_2026_2494_MOESM3_ESM.gif]

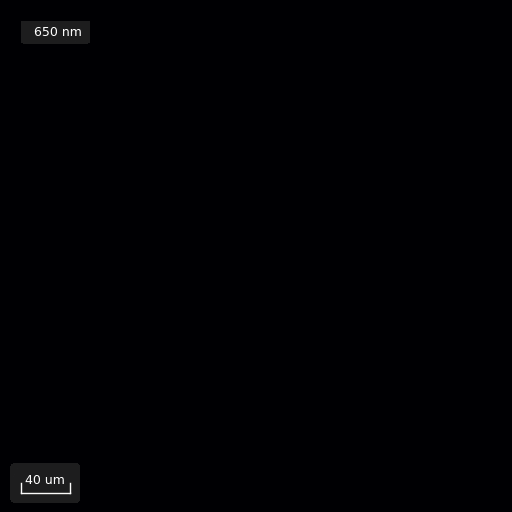

Supplement: Supplementary file 4 — Hyperspectral data cube of an all-vacuum-deposited PSC after 380 h of ageing under ISOS-L-2 protocol. [file 41563_2026_2494_MOESM4_ESM.gif]

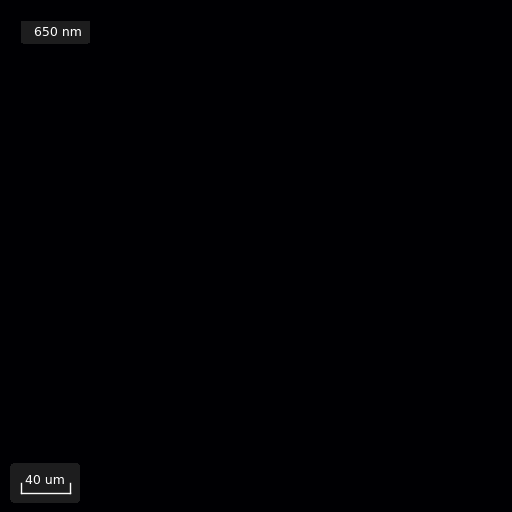

Supplement: Supplementary file 5 — Hyperspectral data cube of a solution-processed PSC before ageing under ISOS-L-2 protocol. [file 41563_2026_2494_MOESM5_ESM.gif]

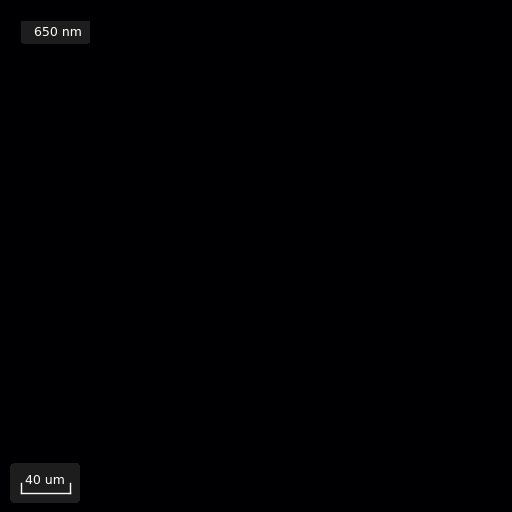

Supplement: Supplementary file 6 — Hyperspectral data cube of a solution-processed PSC after 380 h of ageing under ISOS-L-2 protocol. [file 41563_2026_2494_MOESM6_ESM.gif]
